# If Dung Beetles (Scarabaeidae: Scarabaeinae) Arose in Association with Dinosaurs, Did They Also Suffer a Mass Co-Extinction at the K-Pg Boundary?

**DOI:** 10.1371/journal.pone.0153570

**Published:** 2016-05-04

**Authors:** Nicole L. Gunter, Tom A. Weir, Adam Slipinksi, Ladislav Bocak, Stephen L. Cameron

**Affiliations:** 1 Department of Zoology, Faculty of Science, Palacky University, Olomouc, Czech Republic; 2 Australian National Insect Collection, National Research Collections Australia, Commonwealth Scientific and Industrial Research Organisation, Canberra, ACT, Australia; 3 Earth, Environmental and Biological Sciences School, Science and Engineering Faculty, Queensland University of Technology, Brisbane, QLD, Australia; Onderstepoort Veterinary Institute, SOUTH AFRICA

## Abstract

The evolutionary success of beetles and numerous other terrestrial insects is generally attributed to co-radiation with flowering plants but most studies have focused on herbivorous or pollinating insects. Non-herbivores represent a significant proportion of beetle diversity yet potential factors that influence their diversification have been largely unexamined. In the present study, we examine the factors driving diversification within the Scarabaeidae, a speciose beetle family with a range of both herbivorous and non-herbivorous ecologies. In particular, it has been long debated whether the key event in the evolution of dung beetles (Scarabaeidae: Scarabaeinae) was an adaptation to feeding on dinosaur or mammalian dung. Here we present molecular evidence to show that the origin of dung beetles occurred in the middle of the Cretaceous, likely in association with dinosaur dung, but more surprisingly the timing is consistent with the rise of the angiosperms. We hypothesize that the switch in dinosaur diet to incorporate more nutritious and less fibrous angiosperm foliage provided a palatable dung source that ultimately created a new niche for diversification. Given the well-accepted mass extinction of non-avian dinosaurs at the Cretaceous-Paleogene boundary, we examine a potential co-extinction of dung beetles due to the loss of an important evolutionary resource, i.e., dinosaur dung. The biogeography of dung beetles is also examined to explore the previously proposed “out of Africa” hypothesis. Given the inferred age of Scarabaeinae as originating in the Lower Cretaceous, the major radiation of dung feeders prior to the Cenomanian, and the early divergence of both African and Gondwanan lineages, we hypothesise that that faunal exchange between Africa and Gondwanaland occurred during the earliest evolution of the Scarabaeinae. Therefore we propose that both Gondwanan vicariance and dispersal of African lineages is responsible for present day distribution of scarabaeine dung beetles and provide examples.

## Introduction

Recent explanations for the extraordinary diversity of insects, both at the species- and higher taxonomic levels, have largely centered on the role of co-diversification with flowering plants (angiosperms) [1]. Significantly higher speciation rates within beetle groups associated with angiosperms compared to gymnosperm associated taxa [2] and correlated timing of crown-group radiations of insect families with those of angiosperms [3–6] have been taken as strong evidence for the dependence of major insect radiations on angiosperm evolution. The majority of studies in this area, however, concern taxa with tight ecological associations with plants (i.e. herbivores or pollinators) and to date no model of the influence of angiosperm evolution on non-herbivorous insects has ever been proposed. Beetles are considered to be the most successful animal lineage in the world, occupying almost all terrestrial habitats, with the ~360–400,000 described species representing ~40% of known insect diversity [7]. While numerous hyperdiverse lineages of herbivorous beetles exist, such as weevils (Curculionidae), leaf beetles (Chrysomelidae) and leaf chafers (Scarabaeidae: Melolonthinae), so do numerous hyperdiverse saprophagous and predatory lineages including rove beetles (Staphylinidae), ground beetles (Carabidae) and darkling beetles (Tenebrionidae). Even though non-herbivores represent ~40% of beetle genera [2], potential evolutionary drivers have largely gone unexamined (c.f. Hunt et al. [8]), but given that many saprophagous and predatory beetles do not have specialist diets (host or prey species) coevolution paradigms cannot easily be applied [9].

Scarab beetles (Scarabaeidae) are ideal for testing how lineages with different feeding biologies have radiated in response to major evolutionary events, as the family is composed of phytophagous and saprophagous lineages, with generalist and specialist adaptations to these ecologies. Boasting almost 30,000 species and 1,600 genera, Scarabaeidae is one of the largest beetle families but it is the subfamilies that form the clear division in predominate feeding ecology [10]. The phytophages represent ~70% of the diversity and includes the richest subfamily Melolonthinae (11,000 spp.), that are predominantly leaf feeders, as well as the Rutelinae (4,000 spp.), Cetoniinae (3,300 spp.) and Dynastinae (1,500 spp.) which are more specialized feeders of fruit, flowers, pollen, sap, wood (including dead wood), and tubers, as well as leaves [10]. The saprophagous group contains the Aphodiinae (3,300 spp.) that are more generalized detritivores feeding on dead or decaying matter including leaf litter, fallen logs and flowers, rotting fruits, mushrooms, dung and in some cases carcasses, and the Scarabaeinae (5,000 spp.) that are predominantly specialist dung feeders [10]. Given the sheer diversity of species, it is unsurprising that exceptions to these general feeding biologies exist within lineages, however such specialist taxa represent a small proportion of diversity [10] with many of these feeding ecologies representing derived traits (e.g. pollen feeding Hopliini (Melolonthinae)[11] or necrophagy in Scarabaeinae [12]), thus making scarab beetles ideal for examining broad evolutionary trends.

The Scarabaeoidea first appear in the fossil record in the Middle Jurassic. *Alloioscarabaeus cheni* is a remarkably well preserved fossil from the Jiulongshan formation and is assigned to the superfamily due to the presence of antennae with lamellate clubs and dentate protibia with a terminal apical spur but due to unique wing development cannot be assigned to an extant family [13]. The Jiulongshan formation, Daohugou Village, Inner Mongolia China is considered to represent late Middle Jurassic biota (~165 Ma) based on radiometric dating and biostratigraphic analyses [14–17]. The age of *Alloioscarabaeus* provides a minimum age for the superfamily, however, early diversification of the Scarabaeoidea remains unclear due to gaps in the fossil record and the lack of other high quality fossils. Molecular studies have yet to yield consistent dates for the divergence of Scarabaeoidea, suggesting either a Lower Jurassic (~191.4Ma [8], ~174.3–190.9Ma [18]) or Lower Cretaceous (~141.11 Ma [9]) origin. However, the latter estimate is younger than the age of *Allioscarabaeus* even when considering the 95% confidence interval (161.0–116.87Ma) [9] and may be underestimated. Further molecular evidence suggesting a Lower Jurassic origin of Scarabaeoidea can be taken from the phylogeny of stag beetles (Lucanidae), one of the earliest branching scarabaeoid families. The origin of the stag beetle crown group is estimated as ~160Ma, which is in line with a lower Jurassic origin of the superfamily [19].

To date, only one study has examined the evolution of scarab beetles (Scarabaeidae) using molecular divergence dating methods, and suggested that the timing of diversification tracks the sequential rise of angiosperms and mammals [18]. The study found that the phytophagous lineage diverged ~ 108.9–128.1Ma (mean age of crown group tested using 6 alternative calibrations) while the major melolonthine tribes (leaf feeders) diverged in the Upper Cretaceous and the more specialized angiosperm feeding subfamilies diverged in the Paleogene. Due to these results, the diversification of plant feeding scarabs was attributed to the rise of angiosperms and a temporal lag [18]. Within the saprophagous lineage, Ahrens et al. [18] estimated that the mean crown group ages of Aphodiinae and Scarabaeinae were 65.5–124.7 Ma and 86.6–100.2MA respectively, however no estimate of the origin of this saprophagous clade is explicitly given. Ahrens et al. [18] examine the origin of dung feeding in both lineages, and given that the timing was estimated at 72.6–85.5Ma for the Scarabaeinae and 43.4–78.1Ma for the Aphodiinae, they concluded that it was unlikely that dung feeding scarabs initially fed on dinosaur dung and was therefore associated with the rise of mammals.

The study of Ahrens et al. [18] provided the first insights in to the evolutionary history of scarab beetles, however, their 146 species, 4 gene data set could not recover a monophyletic Scarabaeidae. These results are in conflict with the results of a 2 gene, 282 taxa phylogeny of the Superorder Staphyliniformia which recovered a weakly supported sister relationship between the phytophagous and saprophagous scarab lineages [20]. Despite conflicting evidence that the Scarabaeidae represents a single lineage, their monophyly is supported by numerous apomorphies including larval and adult morphological characters [10]. As such we re-examine the phylogeny of scarab beetles to provide an independent test of the relationships and their divergence dates. We examine the effect of fossil calibration using two different fossil sets and the influence of maximum bound on estimated ages, allowing for a more rigorous test of the impact of potential co-radiations with angiosperms, dinosaurs and mammals on scarab beetle diversification.

## Materials and Methods

### Taxon Sampling

In total, 125 species from the superfamily Scarabaeoidea were collected from Australia and preserved in 96% undenatured ethanol. All necessary permits were obtained for the described study, which complied with all relevant regulations. Field permits were issued from the following State government institution: Department of Parks and Wildlife, Western Australia; Department of National Parks, Sports and Racing, and Department of Environment and Heritage Protection, Queensland; Department of Primary Industries; Office of Environment and Heritage, New South Wales; Department of Environment, Water and Natural Resources, South Australia, and Parks & Wildlife Service, Tasmania. No endangered or protected species were collected during this study. All voucher specimens are deposited at the Australian National Insect Collection, CSIRO, Canberra. Taxon sampling was relatively proportional to the diversity of the Australian scarabaeoid fauna. Additional scarabaeoid sequences representing the global fauna were downloaded from GenBank. Only 7 of 12 families recognized by Bouchard et al. [21] were included, however, the families absent from the study do not occur in Australia [22] so could not be collected during this study, and no sequences were available on GenBank at the time of analysis. The total data set comprised 450 taxa and included 45 non-scarabaeoid polyphagans and adephagans beetles as outgroups. A list of taxa used in analyses is provided in Table 1.

**Table 1:** Taxon and GenBank accession numbers within the phylogenetic analysis.

Superfamily	Family	Subfamily	Tribe	Genus	lab code/ Taxonomy ID	28s	COI	16s	Collection Locality
Bostrichoidea	Bostrichidae	-	-	***Tristaria sp*.**	COL1607	KF802095	KF801929	KF801767	Mt Lindesay NP WA
Bostrichoidea	Bostrichidae	Bostrichinae	-	***Xylotillus sp*.**	COL1328	KF802081	KF801915	KF801752	Goodlands WA
Bostrichoidea	Bostrichidae	Lyctinae	-	***Lyctodon sp*.**	COL375	KF802122	KF801958	KF801794	Tregole NP QLD
Bostrichoidea	Bostrichidae	Lyctinae	-	***Lyctus sp*.**	COL373	KF802120	KF801956	KF801792	Chesterton Range NP QLD
Bostrichoidea	Bostrichidae	Lyctinae	-	***Trogoxylon sp*.**	COL374	KF802121	KF801957	KF801793	Tregole NP QLD
Caraboidea	Carabidae	-	Apotomini	***Apotomus sp*.**	COL222	KF802109	KF801945	KF801781	Charleville QLD
Caraboidea	Carabidae	-	Bembidiini	***Bembidion sp*.**	COL223	KF802110	KF801946	KF801782	Tregole NP QLD
Caraboidea	Carabidae	-	Carabini	***Calosoma sp*.**	COL224	KF802111	KF801947	KF801783	Tregole NP QLD
Caraboidea	Carabidae	-	Harpalini	***Cenogmus sp*.**	COL225	KF802112	KF801948	KF801784	Charleville QLD
Caraboidea	Carabidae	-	Lebiini	***Anomotarus sp*.**	COL221	KF802108	KF801944	KF801780	Charleville QLD
Caraboidea	Carabidae	-	Psydrini	***Amblytelus sp*.**	COL220	KF802107	KF801943	KF801779	Tregole NP QLD
Caraboidea	Dytiscidae	Agabinae	Agabini	***Platynectes sp*.**	COL109	KF802060	KF801894	KF801731	Charleville QLD
Caraboidea	Dytiscidae	Copelatinae	Copelatini	***Copelatus sp*.**	COL112	KF802061	KF801895	KF801732	Cooktown QLD
Caraboidea	Dytiscidae	Dytiscinae	Hydaticini	***Hydaticus sp*.**	COL689	KF802144	KF801980	KF801816	Kuranda QLD
Caraboidea	Dytiscidae	Hydroporinae	Bidessini	***Limbodessus sp*.**	COL690	KF802145	KF801981	-	Black Mountain Rd QLD
Elateroidea	Elateridae	Agrypninae	-	***Heteroderes sp*.**	COL096	KF802045	KF801882	KF801714	Orkadilla SF QLD
Elateroidea	Elateridae	Agrypninae	-	***Paracalais sp*.**	COL076	KF802026	KF801863	KF801695	Tregole NP QLD
Elateroidea	Elateridae	Agrypninae	-	***Pseudotetralobus sp*.**	COL075	KF802025	KF801862	KF801694	Tregole NP QLD
Hydrophiloidea	Histeridae	-	-	***Acritus sp*.**	COL1314	KF802080	KF801914	KF801751	Mt Bartle Frere QLD
Hydrophiloidea	Histeridae	-	-	***Aeletes sp*.**	COL1312	KF802078	KF801912	KF801749	Mt Bartle Frere QLD
Hydrophiloidea	Histeridae	-	-	***Bacaniomorphus sp*.**	COL1313	KF802079	KF801913	KF801750	Mt Hypipamee NP QLD
Hydrophiloidea	Histeridae	-	-	***Notosaprinus sp*.**	COL505	KF802128	KF801964	KF801800	Cania Gorge QLD
Hydrophiloidea	Histeridae	-	-	***Saprinus sp*.**	COL507	KF802129	KF801965	KF801801	Wellington Dam WA
Hydrophiloidea	Histeridae	-	-	***Teretriosoma sp*.**	COL508	KF802130	KF801966	KF801802	Cainbable Quarry QLD
Hydrophiloidea	Hydrophilidae	Acidocerinae	-	***Chasmogenus sp*.**	COL991	KF802165	KF802002	KF801836	Kuranda QLD
Hydrophiloidea	Hydrophilidae	Chaetarthrinae	Anacaenini	***Anacaena sp*.**	COL801	KF802153	KF801990	KF801825	Mt Lewis QLD
Hydrophiloidea	Hydrophilidae	Coelostomatini	-	***Dactylosternum marginale***	COL992	KF802166	KF802003	KF801837	Mt Hypipamee NP QLD
Hydrophiloidea	Hydrophilidae	Enochrinae	-	***Enochrus sp*.**	COL994	KF802168	KF802005	KF801839	New England NP NSW
Hydrophiloidea	Hydrophilidae	Rygmodinae	-	***Pseudohydrobius sp*.**	COL803	KF802155	KF801992	KF801827	Mt Lewis QLD
Hydrophiloidea	Hydrophilidae	Rygmodinae	-	***Rygmostralia sp*.**	COL800	KF802152	KF801989	KF801824	Australia
Hydrophiloidea	Hydrophilidae	Sphaeridiinae	Megasternini	***Pilocnema sp*.**	COL802	KF802154	KF801991	KF801826	Lake Barrine QLD
Hydrophiloidea	Hydrophilidae	Sphaeridiinae	Megasternini	***Pseudoosternum sp*.**	COL804	KF802156	KF801993	KF801828	Baldy MY QLD
Hydrophiloidea	Hydrophilidae	Sphaeridiinae	Omicrini	***Microgioton sp*.**	COL993	KF802167	KF802004	KF801838	Mt Hypipamee NP QLD
Scarabaeoidea	Bolboceratidae	-	-	***Australobolbus sp*.**	COL036	KF802009	KF801843	-	Lamington NP, QLD
Scarabaeoidea	Bolboceratidae	-	-	***Blackbolbus sp*.**	COL1307	KF802076	KF801910	KF801747	Coolcalaya WA
Scarabaeoidea	Bolboceratidae	-	-	***Elephastomus sp*.**	COL193	KF802103	KF801938	KF801776	Berry Beach NSW
Scarabaeoidea	Bolboceratidae	Cnodalonini	-	***Gilletinus sp*.**	COL996	KF802169	KF802006	KF801840	Lamington NP, QLD
Scarabaeoidea	Geotrupidae	Geotrupinae	-	***Geotrupes sp*.**	COL098	KF802047	KF801884	KF801716	Burnie TAS
Scarabaeoidea	Glyphyridae	-	-	*Athypna carceli*	465181	EU084143	EU084039.1	EF487848	-
Scarabaeoidea	Glyphyridae	-	-	*Eulasia sp*. *BMNH 671327*	465236	EU084144	EU084039.1	EF487983	-
Scarabaeoidea	Hybosoridae	Ceratocanthinae	-	***Cyphopisthes sp*.**	COL1011	KF802049	KF801885	KF801718	Barakula SF QLD
Scarabaeoidea	Hybosoridae	Ceratocanthinae	-	***Pterorthochaetes sp*.**	COL1601	KF802094	KF801928	KF801766	East Claudie River QLD
Scarabaeoidea	Hybosoridae	Hyborsorinae	-	*Hybosorus illigeri*	351689	KJ845126	DQ222020	DQ202616	-
Scarabaeoidea	Hybosoridae	Hyborsorinae	-	***Phaeochrous sp*.**	COL046	KF802016	KF801853	KF801685	Ravenshoe QLD
Scarabaeoidea	Hybosoridae	Hyborsorinae	-	*Phaeochrous sp*. *BM678421*	396636	DQ524806	DQ524577.1	DQ681048	-
Scarabaeoidea	Hybosoridae	Hyborsorinae	-	*Phaeochrous sp*. *BM678424*	396637	DQ524809	DQ524578.1	DQ681047	-
Scarabaeoidea	Hybosoridae	Liparochrinae	-	***Liparochrus sp*.**	COL696	KF802146	KF801982	KF801817	Port Hinchinbrook QLD
Scarabaeoidea	Hydraenidae	Hydraeninae	Hydraenini	***Hydraena sp*.**	COL195	-	KF801940	KF801778	Tregole NP QLD
Scarabaeoidea	Hydraenidae	Ochthebiinae	Ochthebiini	***Gymnochthebius sp*.**	COL194	KF802104	KF801939	KF801777	Tregole NP QLD
Scarabaeoidea	Lucanidae	Lampriminae	-	***Lamprima sp*.**	COL544	KF802131	KF801967	KF801803	Sawyers Gully Rd QLD
Scarabaeoidea	Lucanidae	Lucaninae	-	***Cacostomus sp*.**	COL638	KF802141	KF801977	KF801813	Garradunga QLD
Scarabaeoidea	Lucanidae	Lucaninae	-	***Figulus sp*.**	COL1329	KF802082	KF801916	KF801753	Flinders Ranges NP SA
Scarabaeoidea	Lucanidae	Lucaninae	-	***Lissotes sp*.**	COL1656	KF802098	KF801932	KF801770	Strahan TAS
Scarabaeoidea	Lucanidae	Lucaninae	-	*Platycerus kawadai*	509899	AB426917	AB609410	AB490403	-
Scarabaeoidea	Lucanidae	Lucaninae	-	*Prismognathus angularis*	231743	AB426939	AB427049	AB178299	-
Scarabaeoidea	Lucanidae	Lucaninae	-	***Prosopocoilus sp*. *A***	COL1046	KF802051	KF801886	KF801722	Garradunga QLD
Scarabaeoidea	Lucanidae	Lucaninae	-	***Prosopocoilus sp*. *A***	COL894	KF802162	KF801999	KF801833	Garradunga QLD
Scarabaeoidea	Lucanidae	Lucaninae	-	***Prosopocoilus sp*. *B***	COL1583	KF802087	KF801921	KF801758	East Claudie River QLD
Scarabaeoidea	Lucanidae	Lucaninae	-	***Serrognathus sp*.**	COL639	KF802142	KF801978	KF801814	Garradunga QLD
Scarabaeoidea	Lucanidae	Syndesinae	-	*Ceruchus lignarius*	273953	AB426938	AB427048	AB178311	-
Scarabaeoidea	Passalidae	-	-	***Gonatas sp*.**	COL1585	-	KF801923	KF801759	East Claudie River QLD
Scarabaeoidea	Passalidae	Aolacocyclinae	-	***Aulacocyclus sp*.**	COL545	KF802132	KF801968	KF801804	Tregole NP QLD
Scarabaeoidea	Passalidae	Passalinae	-	***Mastachilus sp*.**	COL1045	KF802050	KF801922	KF801721	Garradunga QLD
Scarabaeoidea	Scarabaeidae	Aclopinae	Phaenognathini	***Phaenognatha sp*.**	COL1591	KF802093	-	KF801765	Iron Range NP QLD
Scarabaeoidea	Scarabaeidae	Aphodiinae	Aphodiini	***Acrossidius sp*.**	COL097	KF802046	KF801883	KF801715	Australia
Scarabaeoidea	Scarabaeidae	Aphodiinae	Aphodiini	***Aphodius sp*.**	COL095	KF802044	KF801881	KF801713	Charleville QLD
Scarabaeoidea	Scarabaeidae	Aphodiinae	Aphodiini	*Harmogaster exarata*	207387	AY132468	AY132385	-	-
Scarabaeoidea	Scarabaeidae	Aphodiinae	Aphodiini	*Neoemadiellus humerosanguineum*	767849	GQ342030	GQ342151	GQ341870	-
Scarabaeoidea	Scarabaeidae	Aphodiinae	Aphodiini	***Podotenus sp*.**	COL067	KF802018	KF801855	KF801687	Lamington NP, QLD
Scarabaeoidea	Scarabaeidae	Aphodiinae	Eupariini	*Afrodiastictus sp*. *BMNH703538*	464763	EF656729	EF656778	EF656686	-
Scarabaeoidea	Scarabaeidae	Aphodiinae	Eupariini	***Airapus sp*.**	COL637	KF802140	KF801976	KF801812	Clohesy River QLD
Scarabaeoidea	Scarabaeidae	Aphodiinae	Eupariini	*Australammoecius occidentalis*	207377	EF656732	EF656781	EF487822	-
Scarabaeoidea	Scarabaeidae	Aphodiinae	Eupariini	***Australammoecius sp*.**	COL1574	KF802085	KF801919	KF801756	East Claudie River QLD
Scarabaeoidea	Scarabaeidae	Aphodiinae	Proctophanini	***Australaphodius sp*.**	COL1572	KF802083	KF801917	KF801754	Avon Valley NP WA
Scarabaeoidea	Scarabaeidae	Aphodiinae	Proctophanini	***Harmogaster sp*.**	COL1573	KF802084	KF801918	KF801755	Lake Urawa Nat Res NSW
Scarabaeoidea	Scarabaeidae	Aphodiinae	Psammodiini	***Aphodopsammobius sp*.**	COL1280	KF802069	KF801902	KF801740	Paroo-Darling NP NSW
Scarabaeoidea	Scarabaeidae	Aphodiinae	Rhyparini	***Rhyparus sp*.**	COL1308	KF802077	KF801911	KF801748	Garradunga QLD
Scarabaeoidea	Scarabaeidae	Cetoniinae	Cetoniini	*Chiloloba acuta*	383520	DQ524778	DQ524540	DQ680981	-
Scarabaeoidea	Scarabaeidae	Cetoniinae	Cetoniini	***Glycyphana sp*.**	COL072	KF802022	KF801859	KF801691	Morven QLD
Scarabaeoidea	Scarabaeidae	Cetoniinae	Cetoniini	*Heterocnemis graeca*	465253	EU084147.1	EU084042.1	EF487942.1	-
Scarabaeoidea	Scarabaeidae	Cetoniinae	Cetoniini	***Protaetia sp*.**	COL192	KF802102	KF801937	KF801775	Mt Crosby QLD
Scarabaeoidea	Scarabaeidae	Cetoniinae	Goliathinini	*Heterorrhina micans*	383522	DQ524738	DQ524507	DQ681041	-
Scarabaeoidea	Scarabaeidae	Cetoniinae	Schizorhinini	***Bisallardiana sp*.**	COL083	KF802033	KF801870	KF801702	Australia
Scarabaeoidea	Scarabaeidae	Cetoniinae	Schizorhinini	***Chlorobapta sp*.**	COL1757	KF802101	KF801935	KF801773	Mt Remarkable NP SA
Scarabaeoidea	Scarabaeidae	Cetoniinae	Schizorhinini	***Chondropyga sp*.**	COL088	KF802038	KF801875	KF801707	Mt Canobolas NSW
Scarabaeoidea	Scarabaeidae	Cetoniinae	Schizorhinini	***Dilochrosis sp*.**	COL1053	KF802056	KF801891	KF801727	[ex culture WA]
Scarabaeoidea	Scarabaeidae	Cetoniinae	Schizorhinini	***Eupoecila sp*.**	COL082	KF802032	KF801869	KF801701	[MOR 2 8.12.08]
Scarabaeoidea	Scarabaeidae	Cetoniinae	Schizorhinini	***Hemipharis sp*.**	COL1586	KF802088	KF801924	KF801760	Georgetown QLD
Scarabaeoidea	Scarabaeidae	Cetoniinae	Schizorhinini	***Ischiopsopha sp*.**	COL1049	KF802052	KF801887	KF801723	Garradunga QLD
Scarabaeoidea	Scarabaeidae	Cetoniinae	Schizorhinini	***Lomaptera sp*.**	COL1755	KF802099	KF801933	KF801771	East Claudie River QLD
Scarabaeoidea	Scarabaeidae	Cetoniinae	Schizorhinini	***Lyraphora sp*.**	COL1055	KF802058	KF801893	KF801729	Garradunga QLD
Scarabaeoidea	Scarabaeidae	Cetoniinae	Schizorhinini	***Lyraphora sp*.**	COL092	KF802042	KF801879	KF801711	Magnetic Island, QLD
Scarabaeoidea	Scarabaeidae	Cetoniinae	Schizorhinini	***Neorrhina sp*.**	COL091	KF802041	KF801878	KF801710	Comway Range NP QLD
Scarabaeoidea	Scarabaeidae	Cetoniinae	Schizorhinini	***Pseudoclithria sp*.**	COL1756	KF802100	KF801934	KF801772	Marble Range SA
Scarabaeoidea	Scarabaeidae	Cetoniinae	Schizorhinini	***Trichaulax sp*.**	COL1050	KF802053	KF801888	KF801724	Georgetown QLD
Scarabaeoidea	Scarabaeidae	Cetoniinae	Stenotarsiini	***Mycterophallus sp*.**	COL630	KF802134	KF801970	KF801806	Garradunga QLD
Scarabaeoidea	Scarabaeidae	Cetoniinae	Valgini	***Microvalgus sp*.**	COL1006	KF802048	-	KF801717	Morton NP NSW
Scarabaeoidea	Scarabaeidae	Cetoniinae	Valgini	*Microvalgus sp*. *BMNH 671321*	465292	EU084294	EU084141	EF487984	-
Scarabaeoidea	Scarabaeidae	Dynastinae	Cyclocephalini	*Cyclocephala sp*. *BMNH 670887*	465219	JN969246	JN969202	EF487979	-
Scarabaeoidea	Scarabaeidae	Dynastinae	Dynastini	***Xylotrupes sp*.**	COL090	KF802040	KF801877	KF801709	Finch Hatton QLD
Scarabaeoidea	Scarabaeidae	Dynastinae	Oryctoderini	***Onychionyx sp*.**	COL1587	KF802089	KF801925	KF801761	East Claudie River QLD
Scarabaeoidea	Scarabaeidae	Dynastinae	Oryctoderini	***Oryctoderus sp*.**	COL1588	KF802090	KF801926	KF801762	East Claudie River QLD
Scarabaeoidea	Scarabaeidae	Dynastinae	Pentodontini	*Alissonotum binodulum*	383518	DQ524782	DQ524544	DQ680957	-
Scarabaeoidea	Scarabaeidae	Dynastinae	Pentodontini	*Alissonotum simile*	383519	DQ524709	DQ524481	DQ681016	-
Scarabaeoidea	Scarabaeidae	Dynastinae	Pentodontini	***Carneodon sp*.**	COL893	KF802161	KF801998	KF801832	Narrabri NSW
Scarabaeoidea	Scarabaeidae	Dynastinae	Pentodontini	***Cheiroplatys sp*.**	COL1051	KF802054	KF801889	KF801725	Garradunga QLD
Scarabaeoidea	Scarabaeidae	Dynastinae	Pentodontini	*Heteronchyus lioderes*	383500	DQ524753	DQ524558	DQ680955	
Scarabaeoidea	Scarabaeidae	Dynastinae	Pentodontini	***Heteronyx sp*.**	COL043	-	KF801850	KF801683	Borenore NSW
Scarabaeoidea	Scarabaeidae	Dynastinae	Pentodontini	***Metanastes sp*.**	COL034	KF802007	KF801841	-	Lamington NP, QLD
Scarabaeoidea	Scarabaeidae	Dynastinae	Pentodontini	***Neocorynophyllus sp*.**	COL633	KF802137	KF801973	KF801809	Mt Bartle Frere QLD
Scarabaeoidea	Scarabaeidae	Dynastinae	Pentodontini	***Novapus sp*.**	COL071	KF802021	KF801858	KF801690	Tregole NP QLD
Scarabaeoidea	Scarabaeidae	Dynastinae	Pentodontini	*Pentodon idiota*	465151	EU084151	EU084045.1	EF487918	-
Scarabaeoidea	Scarabaeidae	Dynastinae	Pentodontini	*Phyllognathus dionysius*	465331	EU084152	EF487737	EF487944	-
Scarabaeoidea	Scarabaeidae	Dynastinae	Pentodontini	*Pimelopus dubius*	465333	JN969249	EF487738	EF487960	-
Scarabaeoidea	Scarabaeidae	Dynastinae	Pentodontini	***Semanopterus sp*. *A***	COL035	KF802008	KF801842	-	Lamington NP, QLD
Scarabaeoidea	Scarabaeidae	Dynastinae	Pentodontini	***Semanopterus sp*. *B***	COL1306	KF802075	KF801909	KF801746	Mallee Cliffs NP NSW
Scarabaeoidea	Scarabaeidae	Dynastinae	Pentodontini	***Trissodon sp*.**	COL1277	KF802067	KF801900	KF801738	Walpole WA
Scarabaeoidea	Scarabaeidae	Dynastinae	Phileurini	***Cryptodus sp*.**	COL070	KF802020	KF801857	KF801689	Tregole NP QLD
Scarabaeoidea	Scarabaeidae	Dynastinae	Phileurini	***Eophileurus sp*.**	COL1054	KF802057	KF801892	KF801728	Garradunga QLD
Scarabaeoidea	Scarabaeidae	Melolonthinae	-	*Melolonthinae sp*. *BMNH 671352*	465028	EU084232	EF487751	EF487961	-
Scarabaeoidea	Scarabaeidae	Melolonthinae	Ablaberini	*Idaeserica sp*. *BMNH 671445*	465264	EU084204	EU084074	EF487963	-
Scarabaeoidea	Scarabaeidae	Melolonthinae	Automoliini	***Automolius sp*.**	COL1283	KF802072	KF801905	KF801743	Albany to Denmark WA
Scarabaeoidea	Scarabaeidae	Melolonthinae	Automoliini	*Automolus humilis*	465184	EU084170	EF487745	EF487883	-
Scarabaeoidea	Scarabaeidae	Melolonthinae	Chasmatopterini	*Chasmatopterus sp*. *BMNH 694788*	465193	EU084173	EF487988	EF487747	-
Scarabaeoidea	Scarabaeidae	Melolonthinae	Colymbomorphini	***Chariochilus sp*.**	COL632	KF802136	KF801972	KF801808	Garradunga QLD
Scarabaeoidea	Scarabaeidae	Melolonthinae	Colymbomorphini	***Colymbomorpha sp*.**	COL089	KF802039	KF801876	KF801708	Australia [WA S41]
Scarabaeoidea	Scarabaeidae	Melolonthinae	Colymbomorphini	***Xylonichus sp*.**	COL066	KF802017	KF801854	KF801686	Brindabellas ACT
Scarabaeoidea	Scarabaeidae	Melolonthinae	Diphucephalini	***Diphucephala sp*.**	COL081	KF802031	KF801868	KF801700	Australia [TAB08 29.11.09]
Scarabaeoidea	Scarabaeidae	Melolonthinae	Diplotaxini	*Ceratogonia bicornuta*	465190	EU084172	EU084056	EF487849	-
Scarabaeoidea	Scarabaeidae	Melolonthinae	Enariini	*Apiencya sp*. *BMNH 671495*	465173	EU084164	EU084053	EF487939	-
Scarabaeoidea	Scarabaeidae	Melolonthinae	Enariini	*Cherbezatina strigosa*	465198	EU084174	EU084057	EF487934	-
Scarabaeoidea	Scarabaeidae	Melolonthinae	Enariini	*Varencya sp*. *BMNH 671503*	465356	EU084287	EU084136	EF487982	-
Scarabaeoidea	Scarabaeidae	Melolonthinae	Heteronycini	***Heteronychus sp*.**	COL087	KF802037	KF801874	KF801706	Daintree QLD
Scarabaeoidea	Scarabaeidae	Melolonthinae	Heteronycini	***Neoheteronyx sp*.**	COL042	-	KF801849	-	Lamington NP, QLD
Scarabaeoidea	Scarabaeidae	Melolonthinae	Hopliini	*Apomorphochelus sp*. *BMNH 671482*	465175	EU084154	EF487740	EF487996	-
Scarabaeoidea	Scarabaeidae	Melolonthinae	Hopliini	*Ceratochelus sp*. *SA1*	980922	HQ599140	HQ599098	HQ711580	-
Scarabaeoidea	Scarabaeidae	Melolonthinae	Hopliini	*Clania sp*. *'macgregori' DA-2011*	1033759	HQ599143	HQ599100	HQ711557	-
Scarabaeoidea	Scarabaeidae	Melolonthinae	Hopliini	*Congella cf*. *valida DA-2011*	980926	HQ599146	HQ599102	HQ711601	-
Scarabaeoidea	Scarabaeidae	Melolonthinae	Hopliini	*Congella sp*. *'tesselatula' DA-2011*	1033750	HQ599145	HQ599101	HQ711599	-
Scarabaeoidea	Scarabaeidae	Melolonthinae	Hopliini	*Cylinchus sp*. *1 DA-2011*	980928	HQ599147.1	HQ599103.1	HQ711582.1	-
Scarabaeoidea	Scarabaeidae	Melolonthinae	Hopliini	*Dolichoplia sp*. *'longula' DA-2011*	1033761	HQ599150	HQ599105	HQ711559	-
Scarabaeoidea	Scarabaeidae	Melolonthinae	Hopliini	*Echyra umbrina*	465227	EU084155.1	EU084046.1	EF487837.1	-
Scarabaeoidea	Scarabaeidae	Melolonthinae	Hopliini	*Eriesthis cf*. *'rhodesiana' DA-2011*	1033754	HQ599154.1	HQ599108.1	HQ711581.1	-
Scarabaeoidea	Scarabaeidae	Melolonthinae	Hopliini	*Eriesthis semihirta*	980900	HQ599153.1	HQ599107.1	HQ711593.1	-
Scarabaeoidea	Scarabaeidae	Melolonthinae	Hopliini	*Eriesthis vestita*	980901	HQ599155.1	HQ599109.1	HQ711587.1	-
Scarabaeoidea	Scarabaeidae	Melolonthinae	Hopliini	*Gymnoloma sp*. *'nigra' DA-2011*	1033763	HQ599157.1	HQ599111.1	HQ711576.1	-
Scarabaeoidea	Scarabaeidae	Melolonthinae	Hopliini	*Heterochelus defector*	980904	HQ599158.1	HQ599112.1	HQ711573.1	-
Scarabaeoidea	Scarabaeidae	Melolonthinae	Hopliini	*Heterochelus sp*. *SA2*	980933	HQ599162.1	HQ599116.1	HQ711578.1	-
Scarabaeoidea	Scarabaeidae	Melolonthinae	Hopliini	*Hoplia farinosa*	980884	HQ599165.1	HQ599119.1	HQ711562.1	-
Scarabaeoidea	Scarabaeidae	Melolonthinae	Hopliini	*Hoplia korbi*	980885	HQ599166.1	HQ599120.1	HQ711608.1	-
Scarabaeoidea	Scarabaeidae	Melolonthinae	Hopliini	*Hoplia philanthus*	190499	HQ599167.1	HQ599121.1	HQ711597.1	-
Scarabaeoidea	Scarabaeidae	Melolonthinae	Hopliini	*Hopliini sp*. *DA-2011*	980935	HQ599170.1	HQ599122.1	HQ711585.1	-
Scarabaeoidea	Scarabaeidae	Melolonthinae	Hopliini	*Lepithrix cf*. *'forsteri' DA-2011*	1033767	HQ599170.1	HQ599122.1	HQ711585.1	-
Scarabaeoidea	Scarabaeidae	Melolonthinae	Hopliini	*Michaeloplia montana*	465289	EU084157.1	EU084048.1	EF487836.1	-
Scarabaeoidea	Scarabaeidae	Melolonthinae	Hopliini	*Microplus cf*. *nemoralis DA-2011*	980940	HQ599173.1	HQ599125.1	HQ711595.1	-
Scarabaeoidea	Scarabaeidae	Melolonthinae	Hopliini	*Monochelus cf*. *jucundus spSA1*	980941	HQ599175.1	HQ599127.1	HQ711565.1	-
Scarabaeoidea	Scarabaeidae	Melolonthinae	Hopliini	*Monochelus sp*. *'laetus' DA-2011*	1033751	HQ599174.1	HQ599126.1	HQ711577.1	-
Scarabaeoidea	Scarabaeidae	Melolonthinae	Hopliini	*Omocrates sp*. *2 DA-2011*	980944	HQ599177.1	HQ599128.1	HQ711567.1	-
Scarabaeoidea	Scarabaeidae	Melolonthinae	Hopliini	*Pachycnema calviniana*	980914	HQ599174.1	HQ599126.1	HQ711577.1	-
Scarabaeoidea	Scarabaeidae	Melolonthinae	Hopliini	*Paramorphochelus agricola*	465325	EU084158.1	EU084049.1	EF487923.1	-
Scarabaeoidea	Scarabaeidae	Melolonthinae	Liparetrini	***Colobostoma sp*.**	COL074	KF802024	KF801861	KF801693	Orkadilla SF QLD
Scarabaeoidea	Scarabaeidae	Melolonthinae	Liparetrini	*Colpochila sp*. *BMNH 670928*	465204	EU084176.1	EU084059.1	EF487981.1	-
Scarabaeoidea	Scarabaeidae	Melolonthinae	Liparetrini	***Dikellites sp*.**	COL1305	KF802074	KF801908	KF801745	Useless Loop RD WA
Scarabaeoidea	Scarabaeidae	Melolonthinae	Liparetrini	***genus near Engyopsina***	COL631	KF802135	KF801971	KF801807	Garradunga QLD
Scarabaeoidea	Scarabaeidae	Melolonthinae	Liparetrini	***Harpechys sp*.**	COL093	KF802043	KF801880	KF801712	Orkadilla SF QLD
Scarabaeoidea	Scarabaeidae	Melolonthinae	Liparetrini	***Liparetrus sp*.**	COL084	KF802034	KF801871	KF801703	Australia [MOB HN2 2.12.09]
Scarabaeoidea	Scarabaeidae	Melolonthinae	Liparetrini	*Liparetrus sp*. *BMNH 671322*	465282	EU084210.1	EU084077.1	EF487964.1	-
Scarabaeoidea	Scarabaeidae	Melolonthinae	Liparetrini	***Neso sp*.**	COL1653	KF802097	KF801931	KF801769	Pascoe River Xing QLD
Scarabaeoidea	Scarabaeidae	Melolonthinae	Liparetrini	***Odontria sp*.**	COL1856	-	KF801936	KF801774	Lincoln NEW ZEALAND
Scarabaeoidea	Scarabaeidae	Melolonthinae	Liparetrini	***Paronyx sp*.**	COL885	KF802159	KF801996	-	Thaiki Creek QLD
Scarabaeoidea	Scarabaeidae	Melolonthinae	Liparetrini	***Teluroides sp*.**	COL1281	KF802070	KF801903	KF801741	Avon Valley NP WA
Scarabaeoidea	Scarabaeidae	Melolonthinae	Macrodactylini	*Diphycerus sp*. *BMNH 677855*	396625	DQ524745.1	-	DQ680982.1	-
Scarabaeoidea	Scarabaeidae	Melolonthinae	Macrodactylini	*Isonychus sp*. *Arg2*	980890	HQ599181.1	HQ599132.1	HQ711606.1	-
Scarabaeoidea	Scarabaeidae	Melolonthinae	Macrodactylini	*Liogenys sp*. *Arg1*	980937	HQ599136.1	HQ599095.1	HQ711598.1	-
Scarabaeoidea	Scarabaeidae	Melolonthinae	Macrodactylini	*Plectris sp*. *PER1*	980946	HQ599183.1	HQ599134.1	HQ711591.1	-
Scarabaeoidea	Scarabaeidae	Melolonthinae	Maechidiini	***Epholcis sp*. *A***	COL1056	KF802059	-	KF801730	Tinaroo Waters QLD
Scarabaeoidea	Scarabaeidae	Melolonthinae	Maechidiini	***Epholcis sp*. *B***	COL718	KF802147	KF801983	KF801818	Barakula SF QLD
Scarabaeoidea	Scarabaeidae	Melolonthinae	Maechidiini	***Maechidius sp*.**	COL038	KF802011	KF801845	-	Springbrook QLD
Scarabaeoidea	Scarabaeidae	Melolonthinae	Maechidiini	*Maechidius sp*. *BMNH 670841*	465287	EU084211.1	EF487765.1	EF487974.1	-
Scarabaeoidea	Scarabaeidae	Melolonthinae	Melolonthini	*Cyphonoxia kircheri*	207353	AY132477.1	AY132393.1	-	-
Scarabaeoidea	Scarabaeidae	Melolonthinae	Melolonthini	*Dasylepida ishigakiensis*	454919	-	AB332100.1	AB332108.1	-
Scarabaeoidea	Scarabaeidae	Melolonthinae	Melolonthini	***Dermolepida sp*.**	COL1052	KF802055	KF801890	KF801726	Garradunga QLD
Scarabaeoidea	Scarabaeidae	Melolonthinae	Melolonthini	*Dichecephala ovata*	465223	EU084182.1	EF487750.1	EF487843.1	-
Scarabaeoidea	Scarabaeidae	Melolonthinae	Melolonthini	*Empecta sicardi*	465229	EU084184.1	EU084063.1	EF487958.1	-
Scarabaeoidea	Scarabaeidae	Melolonthinae	Melolonthini	*Enaria boissayei*	465231	EU084183.1	EU084062.1	EF487908.1	-
Scarabaeoidea	Scarabaeidae	Melolonthinae	Melolonthini	*Euthora sp*. *BMNH 671497*	465240	EU084188.1	EU084065.1	EF487877.1	-
Scarabaeoidea	Scarabaeidae	Melolonthinae	Melolonthini	*Eutrichesis pilosicollis*	465242	EU084187.1	EU084064.1	EF487935.1	-
Scarabaeoidea	Scarabaeidae	Melolonthinae	Melolonthini	*Haplidia transversa*	465248	EU084190.1	EU084066.1	EF487920.1	-
Scarabaeoidea	Scarabaeidae	Melolonthinae	Melolonthini	*Holotrichia diomphalia*	33394	-	HM180628.1	JX112789.1	-
Scarabaeoidea	Scarabaeidae	Melolonthinae	Melolonthini	*Hoplochelus piliger*	465257	EU084195.1	EU084069.1	EF487969.1	-
Scarabaeoidea	Scarabaeidae	Melolonthinae	Melolonthini	*Idionychus excisus*	383523	DQ524587.1	DQ524373.1	DQ680903.1	-
Scarabaeoidea	Scarabaeidae	Melolonthinae	Melolonthini	*Lepidiota albistigma*	383503	DQ524590.1	DQ524367.1	DQ680878.1	-
Scarabaeoidea	Scarabaeidae	Melolonthinae	Melolonthini	***Lepidiota sp*.**	COL629	KF802133	KF801969	KF801805	Garradunga QLD
Scarabaeoidea	Scarabaeidae	Melolonthinae	Melolonthini	*Lepidiota stradbrokensis*	465128	EU084209.1	EF487763.1	EF487881.1	-
Scarabaeoidea	Scarabaeidae	Melolonthinae	Melolonthini	*Melolonthinae sp*. *BMNH 671352*	465028	EU084209.1	EF487763.1	EF487881.1	-
Scarabaeoidea	Scarabaeidae	Melolonthinae	Melolonthini	*Schizonycha sp*. *SchyzRent*	207290	AY132498.1	AY132405.1	-	-
Scarabaeoidea	Scarabaeidae	Melolonthinae	Melolonthini	*Sophrops sp*. *5 DA 2006*	383525	DQ524748.1	DQ524516.1	DQ680963.1	-
Scarabaeoidea	Scarabaeidae	Melolonthinae	Pachyderini	*Buettikeria echinosa*	465188	EU084171.1	EF487746.1	EF487789.1	-
Scarabaeoidea	Scarabaeidae	Melolonthinae	Pachypodini	*Pachypus candidae*	465321	EU084258.1	EF487764.1	EF487819.1	-
Scarabaeoidea	Scarabaeidae	Melolonthinae	Rhizotrogini	*Amphimallon solstitiale*	360071	EU084161.1	EF487741.1	EF487948.1	-
Scarabaeoidea	Scarabaeidae	Melolonthinae	Scitalini	***Gnaphalopoda sp*.**	COL073	KF802023	KF801860	KF801692	Charleville QLD
Scarabaeoidea	Scarabaeidae	Melolonthinae	Scitalini	***Homolotropus sp*.**	COL848	KF802158	KF801995	KF801830	Tinaroo Falls QLD
Scarabaeoidea	Scarabaeidae	Melolonthinae	Scitalini	***Ophropyx sp*.**	COL086	KF802036	KF801873	KF801705	Stanthorpe QLD
Scarabaeoidea	Scarabaeidae	Melolonthinae	Scitalini	***Scitala sp*.**	COL077	KF802027	KF801864	KF801696	Queanbeyan NSW
Scarabaeoidea	Scarabaeidae	Melolonthinae	Scitalini	*Scitalia aureorufa*	365804	EU084261.1	EF521878.1	EF487986.1	-
Scarabaeoidea	Scarabaeidae	Melolonthinae	Scitalini	***Sericesthis sp*.**	COL039	KF802012	KF801846	-	Cainbable Quarry QLD
Scarabaeoidea	Scarabaeidae	Melolonthinae	Scitalini	***Sericesthis sp*.**	COL085	KF802035	KF801872	KF801704	Stanthorpe QLD
Scarabaeoidea	Scarabaeidae	Melolonthinae	Sericini	*Ablaberoides sp*. *BMNH 671485*	465165	EU084159.1	EU084050.1	EF487851.1	-
Scarabaeoidea	Scarabaeidae	Melolonthinae	Sericini	*Allokotarsa clypeata*	465167	EU084160.1	EF487739.1	EF487945.1	-
Scarabaeoidea	Scarabaeidae	Melolonthinae	Sericini	***Ancylonyx sp*.**	COL634	KF802138	KF801974	KF801810	Garradunga QLD
Scarabaeoidea	Scarabaeidae	Melolonthinae	Sericini	*Anomalophylla sp*. *BMNH 678368*	465171	EU084162.1	EU084051.1	EF487993.1	-
Scarabaeoidea	Scarabaeidae	Melolonthinae	Sericini	*Anomalophylla tristicula*	465170	EU084163.1	EU084052.1	EF487949.1	-
Scarabaeoidea	Scarabaeidae	Melolonthinae	Sericini	*Astaena sp*. *BMNH671333*	465177	-	EF487742.1	EF487966.1	-
Scarabaeoidea	Scarabaeidae	Melolonthinae	Sericini	*Chrysoserica stebnickae*	465202	EU084175.1	EU084058.1	EF487859.1	-
Scarabaeoidea	Scarabaeidae	Melolonthinae	Sericini	*Comaserica crinita*	465206	EU084198.1	EF487758.1	EF487946.1	-
Scarabaeoidea	Scarabaeidae	Melolonthinae	Sericini	*Comaserica sp*. *BMNH 671397*	465208	EU084177.1	EF487748.1	EF487852.1	-
Scarabaeoidea	Scarabaeidae	Melolonthinae	Sericini	*Gynaecoserica variipennis*	465246	EU084189.1	EF487752.1	EF487968.1	-
Scarabaeoidea	Scarabaeidae	Melolonthinae	Sericini	*Hellaserica elongata*	465251	EU084191.1	EF487753.1	EF487885.1	-
Scarabaeoidea	Scarabaeidae	Melolonthinae	Sericini	*Hymenoplia fulvipennis*	478133	EU084197.1	FJ847246.1	FJ956719.1	-
Scarabaeoidea	Scarabaeidae	Melolonthinae	Sericini	*Hymenoplia lineolata*	465259	EU084196.1	EF487759.1	EF487803.1	-
Scarabaeoidea	Scarabaeidae	Melolonthinae	Sericini	*Hyposerica sp*. *BMNH 671398*	465262	EU084202.1	EF487756.1	EF487845.1	-
Scarabaeoidea	Scarabaeidae	Melolonthinae	Sericini	*Lamproserica sp*. *BMNH 670867*	465273	EU084206.1	EU084075.1	EF487887.1	-
Scarabaeoidea	Scarabaeidae	Melolonthinae	Sericini	*Lamproserica sp*. *BMNH 671446*	465274	EU084207.1	EF487761.1	EF487927.1	-
Scarabaeoidea	Scarabaeidae	Melolonthinae	Sericini	*Lepiserica sp*. *BMNH 671451*	465279	JN969252.1	EF487762.1	EF487924.1	-
Scarabaeoidea	Scarabaeidae	Melolonthinae	Sericini	*Maladera affinis*	383504	DQ524804.1	DQ524575.1	DQ681005.1	-
Scarabaeoidea	Scarabaeidae	Melolonthinae	Sericini	*Maladera basalis*	465129	EU084212.1	EU084078.1	EF487889.1	-
Scarabaeoidea	Scarabaeidae	Melolonthinae	Sericini	*Maladera burmeisteri*	465130	EU084213.1	EU084079.1	EF487890.1	-
Scarabaeoidea	Scarabaeidae	Melolonthinae	Sericini	*Maladera cardoni*	383505	DQ524810.1	DQ524567.1	DQ681011.1	-
Scarabaeoidea	Scarabaeidae	Melolonthinae	Sericini	***Neophyllotocus sp*.**	COL1304	KF802073	KF801907	KF801744	Kalbarri NP WA
Scarabaeoidea	Scarabaeidae	Melolonthinae	Sericini	*Omaloplia nigromarginata*	465148	EU084255.1	EF487770.1	EF487791.1	-
Scarabaeoidea	Scarabaeidae	Melolonthinae	Sericini	*Omaloplia ruricola*	465149	EU084256.1	EF487771.1	EF487790.1	-
Scarabaeoidea	Scarabaeidae	Melolonthinae	Sericini	***Phyllotocus sp*.**	COL080	KF802030	KF801867	KF801699	Australia [LAN032 24.11.09]
Scarabaeoidea	Scarabaeidae	Melolonthinae	Sericini	***Sphaeroscelis sp*.**	COL1282	KF802071	KF801904	KF801742	Augusta WA
Scarabaeoidea	Scarabaeidae	Melolonthinae	Sericini	*Trochalus sp*. *BMNH 670862*	465354	EU084279.1	EF487778.1	EF487990.1	-
Scarabaeoidea	Scarabaeidae	Melolonthinae	Sericoidini	***Hadrops sp*.**	COL926	KF802163	KF802000	KF801834	Garradunga QLD
Scarabaeoidea	Scarabaeidae	Melolonthinae	Sericoidini	***Telura sp*.**	COL442	KF802123	KF801959	KF801795	Geeveston TAS
Scarabaeoidea	Scarabaeidae	Rutelinae	Adoretini	*Adoretus lasiopygus*	383479	DQ524794.1	DQ524555.1	DQ680980.1	-
Scarabaeoidea	Scarabaeidae	Rutelinae	Adoretini	*Adoretus sp*. *BM677766*	396603	DQ524444.1	DQ524671.1	DQ680986.1	-
Scarabaeoidea	Scarabaeidae	Rutelinae	Adoretini	*Adoretus sp*. *BM677767*	396604	DQ524672.1	DQ524445.1	DQ680964.1	-
Scarabaeoidea	Scarabaeidae	Rutelinae	Adoretini	*Adoretus versutus*	383484	DQ524793.1	DQ524554.1	DQ680953.1	-
Scarabaeoidea	Scarabaeidae	Rutelinae	Adoretini	*Prodoretus truncatus*	465336	EU084292.1	EU084139.1	EF487915.1	-
Scarabaeoidea	Scarabaeidae	Rutelinae	Adoretini	*Trigonostomum mucoreum*	465343	EU084293.1	EU084140.1	EF487916.1	-
Scarabaeoidea	Scarabaeidae	Rutelinae	Anomalini	*Anomala albopilosa*	452287	-	AB330768.1	AB330390.1	-
Scarabaeoidea	Scarabaeidae	Rutelinae	Anomalini	*Anomala bengalensis*	383485	DQ524741.1	DQ680971.1	DQ524510.1	-
Scarabaeoidea	Scarabaeidae	Rutelinae	Anomalini	*Anomala biharensis*	383486	DQ524751.1	DQ524519.1	DQ680975.1	-
Scarabaeoidea	Scarabaeidae	Rutelinae	Anomalini	*Anomala bilobata*	383487	DQ524785.1	DQ524547.1	DQ680977.1	-
Scarabaeoidea	Scarabaeidae	Rutelinae	Anomalini	*Anomala polita*	383491	DQ524742.1	DQ524511.1	DQ680972.1	-
Scarabaeoidea	Scarabaeidae	Rutelinae	Anomalini	*Anomala praenitens*	383492	DQ524792.1	DQ524553.1	DQ681043.1	-
Scarabaeoidea	Scarabaeidae	Rutelinae	Anomalini	*Anomala variegata*	383498	DQ524760.1	DQ524524.1	DQ680938.1	-
Scarabaeoidea	Scarabaeidae	Rutelinae	Anomalini	*Blithopertha sp*. *BMNH 671502*	465186	EU084289.1	EU084137.1	EF487957.1	-
Scarabaeoidea	Scarabaeidae	Rutelinae	Anomalini	*Isoplia lasiosoma*	980908	HQ599172.1	HQ599124.1	HQ711583.1	-
Scarabaeoidea	Scarabaeidae	Rutelinae	Anomalini	*Mimela siliguria*	383524	DQ524724.1	DQ524498.1	DQ680959.1	-
Scarabaeoidea	Scarabaeidae	Rutelinae	Anomalini	*Popillia japonica*	7064	GU226581.1	EF487781.1	EF487886.1	-
Scarabaeoidea	Scarabaeidae	Rutelinae	Anoplognathini	***Anoplognathus sp*.**	COL079	KF802029	KF801866	KF801698	Australia [LAN 59 27.11.09]
Scarabaeoidea	Scarabaeidae	Rutelinae	Anoplognathini	***Anoplostethus sp*. *B***	COL892	KF802160	KF801997	KF801831	Garradunga QLD
Scarabaeoidea	Scarabaeidae	Rutelinae	Anoplognathini	***Calloodes sp*.**	COL1589	KF802091	-	KF801763	East Claudie River QLD
Scarabaeoidea	Scarabaeidae	Rutelinae	Anoplognathini	***Mimadoretus sp*.**	COL719	KF802148	KF801984	KF801819	Garradunga QLD
Scarabaeoidea	Scarabaeidae	Rutelinae	Anoplognathini	***Paraschizognathus sp*.**	COL847	KF802157	KF801994	KF801829	Mt Lewis QLD
Scarabaeoidea	Scarabaeidae	Rutelinae	Anoplognathini	***Repsimus sp*. *A***	COL078	KF802028	KF801865	KF801697	Australia [LAN 31 24.11.09]
Scarabaeoidea	Scarabaeidae	Rutelinae	Anoplognathini	***Repsimus sp*. *B***	COL1590	KF802092	KF801927	KF801764	East Claudie River QLD
Scarabaeoidea	Scarabaeidae	Rutelinae	Rutelini	***Parastasia sp*. *A***	COL1577	KF802086	KF801920	KF801757	East Claudie River QLD
Scarabaeoidea	Scarabaeidae	Rutelinae	Rutelini	***Parastasia sp*. *B***	COL1652	KF802096	KF801930	KF801768	Iron Range NP QLD
Scarabaeoidea	Scarabaeidae	Scarabaeinae	Ateichini	*Ateuchus chrysopyge*	206728	AY131692.1	AY131866.1	AY131502.1	-
Scarabaeoidea	Scarabaeidae	Scarabaeinae	Ateichini	*Ateuchus ecuadorense*	206729	EF656692.1	EF656650.1	EF656741.1	-
Scarabaeoidea	Scarabaeidae	Scarabaeinae	Ateichini	*Ateuchus sp*. *dgi1*	206692	AY131691.1	-	AY131501.1	-
Scarabaeoidea	Scarabaeidae	Scarabaeinae	Ateichini	*Canthidium thalassinum*	206808	AY131697.1	AY131870.1	AY131508.1	-
Scarabaeoidea	Scarabaeidae	Scarabaeinae	Canthonini	***Amphistomus sp*.**	COL037	KF802010	KF801844	-	Lamington NP, QLD
Scarabaeoidea	Scarabaeidae	Scarabaeinae	Canthonini	*Anachalcos convexus*	206794	AY131628.1	AY131809.1	AY131437.1	-
Scarabaeoidea	Scarabaeidae	Scarabaeinae	Canthonini	*Anachalcos suturalis*	206795	AY131629.1	AY131810.1	AY131438.1	-
Scarabaeoidea	Scarabaeidae	Scarabaeinae	Canthonini	*Anonthobium tibiale*	206799	AY131630.1	AY131811.1	AY131439.1	-
Scarabaeoidea	Scarabaeidae	Scarabaeinae	Canthonini	*Apotolamprus cyanescens*	860797	GQ342048.1	GQ342111.1	GQ341886.1	-
Scarabaeoidea	Scarabaeidae	Scarabaeinae	Canthonini	*Apotolamprus darainaensis*	767839	GQ342033.1	GQ342112.1	GQ341872.1	-
Scarabaeoidea	Scarabaeidae	Scarabaeinae	Canthonini	***Aptenocanthon hopsoni***	COL2017	KF802105	KF801941	-	Dooragan NP NSW
Scarabaeoidea	Scarabaeidae	Scarabaeinae	Canthonini	*Aptenocanthon sp*. *dgi1*	206574	AY131631.1	AY131812.1	AY131440.1	-
Scarabaeoidea	Scarabaeidae	Scarabaeinae	Canthonini	***Aptenocanthon winyar***	COL1132	KF802063	KF801897	KF801734	Majors Mt QLD
Scarabaeoidea	Scarabaeidae	Scarabaeinae	Canthonini	*Boletoscapter cornutus*	206803	AY131632.1	AY131813.1	AY131441.1	-
Scarabaeoidea	Scarabaeidae	Scarabaeinae	Canthonini	*Canthon lamprimus*	464772	EF656690.1	EF656739.1	EF656648.1	-
Scarabaeoidea	Scarabaeidae	Scarabaeinae	Canthonini	*Canthon luteicollis*	206812	AY131635.1	AY131815.1	AY131444.1	-
Scarabaeoidea	Scarabaeidae	Scarabaeinae	Canthonini	*Canthon viridis*	206814	AY131637.1	AY131817.1	AY131446.1	-
Scarabaeoidea	Scarabaeidae	Scarabaeinae	Canthonini	*Canthonosoma castelnaui*	206816	AY131638.1	AY131818.1	AY131447.1	-
Scarabaeoidea	Scarabaeidae	Scarabaeinae	Canthonini	*Cephalodesmius quadridens*	206824	AY131639.1	AY131819.1	AY131449.1	-
Scarabaeoidea	Scarabaeidae	Scarabaeinae	Canthonini	*Circellium bacchus*	205291	AY131640.1	AY131820.1	AF499690.1	-
Scarabaeoidea	Scarabaeidae	Scarabaeinae	Canthonini	*Deltochilum pseudoparile*	206843	AY131646.1	AY131826.1	AY131455.1	-
Scarabaeoidea	Scarabaeidae	Scarabaeinae	Canthonini	*Dicranocara deschodti*	392729	EF656714.1	EF656763.1	EF656672.1	-
Scarabaeoidea	Scarabaeidae	Scarabaeinae	Canthonini	***Diorygopyx incomptus***	COL2399	KF802119	KF801955	KF801791	Binnaburra QLD
Scarabaeoidea	Scarabaeidae	Scarabaeinae	Canthonini	***Diorygopyx niger***	COL2020	KF802106	KF801942	-	Dorrigo NP NSW
Scarabaeoidea	Scarabaeidae	Scarabaeinae	Canthonini	*Diorygopyx simpliciclunis*	206856	AY131647.1	AY131827.1	AY847521.1	-
Scarabaeoidea	Scarabaeidae	Scarabaeinae	Canthonini	***Diorygopyx simpliciclunis***	COL2396	KF802118	KF801954	KF801790	Tullawallel Track QLD
Scarabaeoidea	Scarabaeidae	Scarabaeinae	Canthonini	***Diorygopyx sp*.**	COL044	KF802014	KF801852	KF801684	Lamington NP, QLD
Scarabaeoidea	Scarabaeidae	Scarabaeinae	Canthonini	*Dwesasilvasedis medinae*	763525	GQ289762.1	GQ289990.1	GQ289711.1	-
Scarabaeoidea	Scarabaeidae	Scarabaeinae	Canthonini	*Epactoides frontalis*	369517	EU030547.1	EU030591.1	EU030503.1	-
Scarabaeoidea	Scarabaeidae	Scarabaeinae	Canthonini	*Epirinus aeneus*	206741	AY131649.1	AY131829.1	AY131458.1	-
Scarabaeoidea	Scarabaeidae	Scarabaeinae	Canthonini	*Eudinopus dytiscoides*	206863	AY131652.1	AY131832.1	AY131461.1	-
Scarabaeoidea	Scarabaeidae	Scarabaeinae	Canthonini	*Hammondantus psammophilus*	763515	GQ289796.1	GQ290017.1	GQ289743.1	-
Scarabaeoidea	Scarabaeidae	Scarabaeinae	Canthonini	*Hansreia affinis*	206878	AY131653.1	AY131833.1	AY131462.1	-
Scarabaeoidea	Scarabaeidae	Scarabaeinae	Canthonini	*Ignambia fasciculata*	206886	AY131654.1	AY131834.1	AY131463.1	-
Scarabaeoidea	Scarabaeidae	Scarabaeinae	Canthonini	***Lepanus australis***	COL773	KF802149	KF801986	KF801821	Landsborough QLD
Scarabaeoidea	Scarabaeidae	Scarabaeinae	Canthonini	***Lepanus globulus***	COL774	KF802150	KF801987	KF801822	Lake Barrine QLD
Scarabaeoidea	Scarabaeidae	Scarabaeinae	Canthonini	***Lepanus occidentalis***	COL1126	KF802062	KF801896	KF801733	Warren NP WA
Scarabaeoidea	Scarabaeidae	Scarabaeinae	Canthonini	***Lepanus palumensis***	COL948	KF802164	KF802001	KF801835	Paluma QLD
Scarabaeoidea	Scarabaeidae	Scarabaeinae	Canthonini	***Lepanus parapisioniae***	COL775	KF802151	KF801988	KF801823	Lake Barrine QLD
Scarabaeoidea	Scarabaeidae	Scarabaeinae	Canthonini	*Megathoposoma candezei*	206892	AY131657.1	AY131836.1	AY131465.1	-
Scarabaeoidea	Scarabaeidae	Scarabaeinae	Canthonini	***Monoplistes sp*.**	COL041	KF802013	KF801848	-	Lamington NP, QLD
Scarabaeoidea	Scarabaeidae	Scarabaeinae	Canthonini	*Monoplistes sp*. *dgi1*	206576	AY131658.1	AY131837.1	AY131466.1	-
Scarabaeoidea	Scarabaeidae	Scarabaeinae	Canthonini	*Odontoloma pusillum*	206750	AY131661.1	AY131839.1	AY131468.1	-
Scarabaeoidea	Scarabaeidae	Scarabaeinae	Canthonini	*Odontoloma sp 1*	859877	GQ342104.1	GQ342153.1	-	-
Scarabaeoidea	Scarabaeidae	Scarabaeinae	Canthonini	*Onthobium cooki*	206755	AY131662.1	AY131840.1	AY131470.1	-
Scarabaeoidea	Scarabaeidae	Scarabaeinae	Canthonini	*Onthobium sp*. *dgi1*	206716	AY131662.1	AY131840.1	AY131470.1	-
Scarabaeoidea	Scarabaeidae	Scarabaeinae	Canthonini	*Panelus sp*. *dgi1*	206718	AY131664.1	AY131842.1	AY131472.1	-
Scarabaeoidea	Scarabaeidae	Scarabaeinae	Canthonini	*Paronthobium simplex*	206908	AY131665.1	AY131843.1	AY131473.1	-
Scarabaeoidea	Scarabaeidae	Scarabaeinae	Canthonini	***Pseudignambia sp*.**	COL045	KF802015	KF801851	-	Mt Haigh QLD
Scarabaeoidea	Scarabaeidae	Scarabaeinae	Canthonini	*Pseudignambia sp*. *Dgi1*	206580	AY131667.1	AY131845.1	AY131475.1	-
Scarabaeoidea	Scarabaeidae	Scarabaeinae	Canthonini	*Pseudonthobium fracticolloides*	206782	AY131669.1	AY131847.1	AY131477.1	-
Scarabaeoidea	Scarabaeidae	Scarabaeinae	Canthonini	***Sauvagesinella becki***	COL1220	KF802066	KF801899	KF801737	Dwellingup WA
Scarabaeoidea	Scarabaeidae	Scarabaeinae	Canthonini	***Sauvagesinella monstrosa***	COL1210	KF802065	KF801898	KF801736	Walpole River WA
Scarabaeoidea	Scarabaeidae	Scarabaeinae	Canthonini	***Sauvagesinella palustris***	COL1204	KF802064	KF801906	KF801735	Cheynes Beach WA
Scarabaeoidea	Scarabaeidae	Scarabaeinae	Canthonini	*Temnoplectron finnigani*	206929	AY131675.1	AY144790.1	AY131483.1	-
Scarabaeoidea	Scarabaeidae	Scarabaeinae	Canthonini	*Temnoplectron politulum*	206930	AY131676.1	AY144786.1	AY131484.1	-
Scarabaeoidea	Scarabaeidae	scarabaeinae	Canthonini	***Temnoplectron sp*.**	COL636	KF802139	KF801975	KF801811	Mt Bartle Frere QLD
Scarabaeoidea	Scarabaeidae	Scarabaeinae	Coprini	*Catharsius calaharicus*	206818	AY131677.1	AY131852.1	AY131485.1	-
Scarabaeoidea	Scarabaeidae	Scarabaeinae	Coprini	*Catharsius molossus*	206819	AY131678.1	AY131853.1	AY131486.1	-
Scarabaeoidea	Scarabaeidae	Scarabaeinae	Coprini	*Catharsius philus*	206820	AY131679.1	AY131854.1	AY131487.1	-
Scarabaeoidea	Scarabaeidae	Scarabaeinae	Coprini	*Catharsius sesostris*	206821	AY131680.1	AY131855.1	AY131488.1	-
Scarabaeoidea	Scarabaeidae	Scarabaeinae	Coprini	*Copris aeneus*	206731	AY131687.1	AY131863.1	AY131496.1	-
Scarabaeoidea	Scarabaeidae	Scarabaeinae	Coprini	*Copris agnus*	206732	AY131687.1	AY131863.1	AY131496.1	-
Scarabaeoidea	Scarabaeidae	Scarabaeinae	Coprini	*Copris amyntor*	206733	AY131687.1	AY131863.1	AY131496.1	-
Scarabaeoidea	Scarabaeidae	Scarabaeinae	Coprini	*Copris lugubris*	206735	AY131687.1	AY131863.1	AY131863.1	-
Scarabaeoidea	Scarabaeidae	Scarabaeinae	Coprini	*Copris sinicus*	206736	AY131687.1	AY131863.1	AY131496.1	-
Scarabaeoidea	Scarabaeidae	Scarabaeinae	Coprini	*Coptodactyla glabricollis*	206834	AY131687.1	AY131863.1	AY131496.1	-
Scarabaeoidea	Scarabaeidae	Scarabaeinae	Coprini	*Metacatharsius opacus*	206748	AY131688.1	AY131864.1	AY131498.1	-
Scarabaeoidea	Scarabaeidae	Scarabaeinae	Coprini	*Metacatharsius sp*. *dgi1*	206714	AY131689.1	AY131865.1	AY131499.1	-
Scarabaeoidea	Scarabaeidae	Scarabaeinae	Dichotomiini	*Bdelyropsis sp*. *BMNH669447*	206801	EF656696.1	EF656745.1	EF656654.1	-
Scarabaeoidea	Scarabaeidae	Scarabaeinae	Dichotomiini	*Coptorhina sp*. *dgi1*	206697	AY131698.1	AY131871.1	AY131509.1	-
Scarabaeoidea	Scarabaeidae	Scarabaeinae	Dichotomiini	*Demarziella mirifica*	206847	AY131701.1	AY131872.1	AY131512.1	-
Scarabaeoidea	Scarabaeidae	Scarabaeinae	Dichotomiini	*Dichotomius boreus*	206737	AY131703.1	AY131874.1	AY131514.1	-
Scarabaeoidea	Scarabaeidae	Scarabaeinae	Dichotomiini	*Dichotomius parcepunctatus*	206738	AY131704.1	AY131875.1	AY131515.1	-
Scarabaeoidea	Scarabaeidae	Scarabaeinae	Dichotomiini	*Dichotomius sp*. *dgi1*	206699	AY131702.1	AY131873.1	AY131513.1	-
Scarabaeoidea	Scarabaeidae	Scarabaeinae	Dichotomiini	*Dichotomius yucatanus*	206739	AY131705.1	AY131876.1	AY131516.1	-
Scarabaeoidea	Scarabaeidae	Scarabaeinae	Dichotomiini	*Gromphas aeruginosa*	206876	AY131706.1	AY131877.1	AY131517.1	-
Scarabaeoidea	Scarabaeidae	Scarabaeinae	Dichotomiini	*Heliocopris andersoni*	206747	AY131707.1	AY131878.1	AY131518.1	-
Scarabaeoidea	Scarabaeidae	Scarabaeinae	Dichotomiini	*Heliocopris hamadryas*	205297	AY131708.1	AY131879.1	AY131519.1	-
Scarabaeoidea	Scarabaeidae	Scarabaeinae	Dichotomiini	*Macroderes sp*. *dgi1*	206712	AY131709.1	AY131880.1	AY131520.1	-
Scarabaeoidea	Scarabaeidae	Scarabaeinae	Dichotomiini	*Ontherus diabolicus*	206904	AY131710.1	AY131881.1	AY131521.1	-
Scarabaeoidea	Scarabaeidae	Scarabaeinae	Dichotomiini	*Sarophorus costatus*	206924	AY131712.1	AY131883.1	GQ289679.1	-
Scarabaeoidea	Scarabaeidae	Scarabaeinae	Dichotomiini	*Sarophorus tuberculatus*	206925	AY131713.1	AY131884.1	AY131524.1	-
Scarabaeoidea	Scarabaeidae	Scarabaeinae	Dichotomiini	*Uroxys micros*	206787	AY131717.1	AY131886.1	AY131528.1	-
Scarabaeoidea	Scarabaeidae	Scarabaeinae	Dichotomiini	*Uroxys pygmaeus*	206788	AY131718.1	EF656761.1	AY131529.1	-
Scarabaeoidea	Scarabaeidae	Scarabaeinae	Dichotomiini	*Uroxys sp*. *BMNH669339*	464786	EF656694.1	EF656743.1	EF656652.1	-
Scarabaeoidea	Scarabaeidae	Scarabaeinae	Dichotomiini	*Uroxys sp*. *dgi1*	206708	AY131715.1	AY131885.1	AY131526.1	-
Scarabaeoidea	Scarabaeidae	Scarabaeinae	Eucraniini	*Anomiopsoides heteroclyta*	205289	AY131720.1	AY131888.1	AY131531.1	-
Scarabaeoidea	Scarabaeidae	Scarabaeinae	Eucraniini	*Ennearabdus lobocephalus*	206861	AY131721.1	AY131889.1	AY131532.1	-
Scarabaeoidea	Scarabaeidae	Scarabaeinae	Eucraniini	*Eucranium arachnoides*	205295	AY131722.1	AY131890.1	AY131533.1	-
Scarabaeoidea	Scarabaeidae	Scarabaeinae	Eurysternini	*Eurysternus angustulus*	206865	AY131722.1	AY131890.1	AY131533.1	-
Scarabaeoidea	Scarabaeidae	Scarabaeinae	Eurysternini	*Eurysternus caribaeus*	206866	AY131725.1	AY131893.1	AY131536.1	-
Scarabaeoidea	Scarabaeidae	Scarabaeinae	Eurysternini	*Eurysternus hamaticollis*	206867	EF656708.1	AY131537.1	AY131894.1	-
Scarabaeoidea	Scarabaeidae	Scarabaeinae	Eurysternini	*Eurysternus plebejus*	206869	AY131727.1	AY131896.1	AY131539.1	-
Scarabaeoidea	Scarabaeidae	Scarabaeinae	Eurysternini	*Eurysternus velutinus*	206870	AY131728.1	AY131897.1	AY131540.1	-
Scarabaeoidea	Scarabaeidae	Scarabaeinae	Gymnopleurini	*Allogymnopleurus thalassinus*	206790	AY131729.1	AY131898.1	AY131541.1	-
Scarabaeoidea	Scarabaeidae	Scarabaeinae	Gymnopleurini	*Garreta nitens*	206872	AY131730.1	AY131899.1	AY131542.1	-
Scarabaeoidea	Scarabaeidae	Scarabaeinae	Gymnopleurini	*Gymnopleurus virens*	206746	AY131731.1	AY131900.1	AY131543.1	-
Scarabaeoidea	Scarabaeidae	Scarabaeinae	Gymnopleurini	*Paragymnopleurus maurus*	206780	AY131733.1	AY131902.1	AY131545.1	-
Scarabaeoidea	Scarabaeidae	Scarabaeinae	Gymnopleurini	*Paragymnopleurus sp*. *dgi1*	206720	AY131732.1	AY131901.1	AY131544.1	-
Scarabaeoidea	Scarabaeidae	Scarabaeinae	Gymnopleurini	*Paragymnopleurus striatus*	206781	AY131734.1	AY131903.1	AY131546.1	-
Scarabaeoidea	Scarabaeidae	Scarabaeinae	Oniticellini	*Cyptochirus ambiguus*	1165001	AY131735.1	AY131904.1	AY131547.1	-
Scarabaeoidea	Scarabaeidae	Scarabaeinae	Oniticellini	***Euoniticellus sp*.**	COL1279	KF802068	KF801901	KF801739	Walpole WA
Scarabaeoidea	Scarabaeidae	Scarabaeinae	Oniticellini	*Helictopleurus quadripunctatus*	206880	EF656698.1	EF656747.1	EF656656.1	-
Scarabaeoidea	Scarabaeidae	Scarabaeinae	Oniticellini	*Helictopleurus rudicollis*	369553	EF656717.1	EF188183.1	EF656675.1	-
Scarabaeoidea	Scarabaeidae	Scarabaeinae	Oniticellini	*Helictopleurus sp*. *10 MNM 2009*	674440	FJ817989.1	EF656748.1	EF656657.1	-
Scarabaeoidea	Scarabaeidae	Scarabaeinae	Oniticellini	*Helictopleurus sp*. *BMNH 669916*	464777	EF656703.1	EF656752.1	EF656661.1	-
Scarabaeoidea	Scarabaeidae	Scarabaeinae	Oniticellini	*Helictopleurus steineri*	422234	EF656765.1	EF188193.1	EF656674.1	-
Scarabaeoidea	Scarabaeidae	Scarabaeinae	Oniticellini	*Liatongus militaris*	206890	AY131739.1	AY131908.1	AY131552.1	-
Scarabaeoidea	Scarabaeidae	Scarabaeinae	Oniticellini	*Oniticellus egregrus*	1165006	AY131740.1	AY131909.1	AY131553.1	-
Scarabaeoidea	Scarabaeidae	Scarabaeinae	Oniticellini	*Oniticellus planatus*	422237	EF188114.1	EF188204.1	-	-
Scarabaeoidea	Scarabaeidae	Scarabaeinae	Oniticellini	*Tiniocellus sarawacus*	206932	AY131742.1	AY131911.1	AY131555.1	-
Scarabaeoidea	Scarabaeidae	Scarabaeinae	Oniticellini	*Tiniocellus sp*. *BMNH676999*	464785	EF656725.1	EF656774.1	EF656683.1	-
Scarabaeoidea	Scarabaeidae	Scarabaeinae	Oniticellini	*Tiniocellus spinipes*	206933	AY131743.1	AY131743.1	AY131556.1	-
Scarabaeoidea	Scarabaeidae	Scarabaeinae	Oniticellini	*Tragiscus dimidiatus*	206935	AY131744.1	AY131913.1	AY131557.1	-
Scarabaeoidea	Scarabaeidae	Scarabaeinae	Onitini	*Bubas bison*	166326	AY131779.1	AY131938.1	AY131595.1	-
Scarabaeoidea	Scarabaeidae	Scarabaeinae	Onitini	*Bubas bubalus*	166340	AY131780.1	AY131939.1	AY131596.1	-
Scarabaeoidea	Scarabaeidae	Scarabaeinae	Onitini	*Cheironitis hoplosternus*	206730	AY131781.1	AY131940.1	AY131597.1	-
Scarabaeoidea	Scarabaeidae	Scarabaeinae	Onitini	*Heteronitis castelnaui*	206882	AY131782.1	AY131941.1	AY131598.1	-
Scarabaeoidea	Scarabaeidae	Scarabaeinae	Onitini	*Onitis alexis*	206751	AY131783.1	AY131942.1	AY131599.1	-
Scarabaeoidea	Scarabaeidae	Scarabaeinae	Onitini	*Onitis caffer*	206752	AY131784.1	EF656762.1	AY131600.1	-
Scarabaeoidea	Scarabaeidae	Scarabaeinae	Onitini	*Onitis falcatus*	206753	AY131785.1	AY131943.1	AY131601.1	-
Scarabaeoidea	Scarabaeidae	Scarabaeinae	Onitini	***Onitis sp*.**	COL069	KF802019	KF801856	KF801688	Tregole NP QLD
Scarabaeoidea	Scarabaeidae	Scarabaeinae	Onthophagini	*Caccobius binodulus*	206938	AY131745.1	AY131914.1	AY131558.1	-
Scarabaeoidea	Scarabaeidae	Scarabaeinae	Onthophagini	*Caccobius nigritulus*	206939	AY131746.1	AY131915.1	AY131559.1	-
Scarabaeoidea	Scarabaeidae	Scarabaeinae	Onthophagini	*Caccobius schreberi*	166341	AY131747.1	AY131916.1	AY131560.1	-
Scarabaeoidea	Scarabaeidae	Scarabaeinae	Onthophagini	*Cleptocaccobius convexifrons*	206826	AY131748.1	AY131917.1	AY131561.1	-
Scarabaeoidea	Scarabaeidae	Scarabaeinae	Onthophagini	*Digitonthophagus gazella*	206854	AY131750.1	Y131918.1	EF187976.1	-
Scarabaeoidea	Scarabaeidae	Scarabaeinae	Onthophagini	*Euonthophagus carbonarius*	206745	AY131751.1	AY131919.1	AY131564.1	-
Scarabaeoidea	Scarabaeidae	Scarabaeinae	Onthophagini	*Hyalonthophagus alcyon*	206884	AY131752.1	AY131920.1	AY131565.1	-
Scarabaeoidea	Scarabaeidae	Scarabaeinae	Onthophagini	*Milichus apicalis*	206894	AY131753.1	AY131921.1	AY131566.1	-
Scarabaeoidea	Scarabaeidae	Scarabaeinae	Onthophagini	*Onthophagus babirussoides*	206756	AY131754.1	AY131922.1	AY131568.1	-
Scarabaeoidea	Scarabaeidae	Scarabaeinae	Onthophagini	*Onthophagus batesi*	206757	EF656689.1	EF656738.1	EF656647.1	-
Scarabaeoidea	Scarabaeidae	Scarabaeinae	Onthophagini	*Onthophagus bidentatus*	206758	AY131755.1	AY131923.1	AY131569.1	-
Scarabaeoidea	Scarabaeidae	Scarabaeinae	Onthophagini	*Onthophagus championi*	206760	EF656693.1	EF656742.1	EF656651.1	-
Scarabaeoidea	Scarabaeidae	Scarabaeinae	Onthophagini	*Onthophagus clypeatus*	206761	AY131757.1	EF656758.1	AY131572.1	-
Scarabaeoidea	Scarabaeidae	Scarabaeinae	Onthophagini	*Onthophagus crinitis*	206763	AY131759.1	AY131924.1	AY131574.1	-
Scarabaeoidea	Scarabaeidae	Scarabaeinae	Onthophagini	*Onthophagus fimetarius*	206765	AY131760.1	AY131925.1	AY131575.1	-
Scarabaeoidea	Scarabaeidae	Scarabaeinae	Onthophagini	*Onthophagus glabratus*	206767	AY131762.1	AY131926.1	AY131577.1	-
Scarabaeoidea	Scarabaeidae	Scarabaeinae	Onthophagini	*Onthophagus haematopus*	206768	AY131763.1	AY131933.1	AY131590.1	-
Scarabaeoidea	Scarabaeidae	Scarabaeinae	Onthophagini	*Onthophagus mulgravei*	206772	AY131766.1	AY131927.1	AY131582.1	-
Scarabaeoidea	Scarabaeidae	Scarabaeinae	Onthophagini	*Onthophagus obscurior*	206774	AY131768.1	AY131928.1	AY131584.1	-
Scarabaeoidea	Scarabaeidae	Scarabaeinae	Onthophagini	*Onthophagus rorarius*	206776	AY131770.1	AY131929.1	AY131586.1	-
Scarabaeoidea	Scarabaeidae	Scarabaeinae	Onthophagini	*Onthophagus rubicundulus*	206777	AY131771.1	AY131930.1	AY131587.1	-
Scarabaeoidea	Scarabaeidae	Scarabaeinae	Onthophagini	*Onthophagus semiareus*	206778	AY131773.1	AY131932.1	AY131589.1	-
Scarabaeoidea	Scarabaeidae	Scarabaeinae	Onthophagini	*Onthophagus similis*	166359	AY131774.1	AY131933.1	AY131590.1	-
Scarabaeoidea	Scarabaeidae	Scarabaeinae	Onthophagini	***Onthophagus sp*.**	COL040	-	KF801847	-	The Crater NP QLD
Scarabaeoidea	Scarabaeidae	Scarabaeinae	Onthophagini	*Onthophagus sp*. *2 CWC-2007*	476086	EU162517.1	EU162470.1	EU162567.1	-
Scarabaeoidea	Scarabaeidae	Scarabaeinae	Onthophagini	*Onthophagus sp*. *3 CWC-2007*	476087	EU162518.1	-	EU162568.1	-
Scarabaeoidea	Scarabaeidae	Scarabaeinae	Onthophagini	*Onthophagus sp*. *4 CWC-2007*	476088	EU162519.1	EU162471.1	EU162569.1	-
Scarabaeoidea	Scarabaeidae	Scarabaeinae	Onthophagini	*Onthophagus sp*. *5 CWC-2007*	476089	EU162520.1	EU162472.1	EU162570.1	-
Scarabaeoidea	Scarabaeidae	Scarabaeinae	Onthophagini	*Onthophagus sp*. *dgi1*	206577	AY131772.1	AY131931.1	AY131588.1	-
Scarabaeoidea	Scarabaeidae	Scarabaeinae	Onthophagini	*Onthophagus vulpes*	206779	AY131775.1	AY131934.1	AY131591.1	-
Scarabaeoidea	Scarabaeidae	Scarabaeinae	Onthophagini	*Phalops ardea*	206912	AY131776.1	AY131935.1	AY131592.1	-
Scarabaeoidea	Scarabaeidae	Scarabaeinae	Onthophagini	*Proagoderus bicallossus*	206918	AY131777.1	AY131936.1	AY131593.1	-
Scarabaeoidea	Scarabaeidae	Scarabaeinae	Onthophagini	*Proagoderus schwaneri*	206919	AY131778.1	AY131937.1	AY131594.1	-
Scarabaeoidea	Scarabaeidae	Scarabaeinae	Phanaeini	*Coprophanaeus ignecinctus*	506441	EU432267.1	EU477353.1	-	-
Scarabaeoidea	Scarabaeidae	Scarabaeinae	Phanaeini	*Coprophanaeus sp*. *dgi1*	206695	AY131787.1	AY131944.1	AY131603.1	-
Scarabaeoidea	Scarabaeidae	Scarabaeinae	Phanaeini	*Coprophanaeus telamon*	206831	AY131789.1	AY131946.1	AY131605.1	-
Scarabaeoidea	Scarabaeidae	Scarabaeinae	Phanaeini	*Dendropaemon bahianum*	206849	AY131790.1	AY131947.1	AY131606.1	-
Scarabaeoidea	Scarabaeidae	Scarabaeinae	Phanaeini	*Oxysternon conspicillatum*	206906	AY131792.1	AY131948.1	AY131608.1	-
Scarabaeoidea	Scarabaeidae	Scarabaeinae	Phanaeini	*Phanaeus sallei*	206916	AY131793.1	AY131951.1	AY131611.1	-
Scarabaeoidea	Scarabaeidae	Scarabaeinae	Scarabaeini	*Drepanocerus bechynei*	206858	AY131736.1	AY131905.1	AY131548.1	-
Scarabaeoidea	Scarabaeidae	Scarabaeinae	Scarabaeini	*Drepanocerus kirbyi*	206859	AY131737.1	AY131906.1	AY131549.1	-
Scarabaeoidea	Scarabaeidae	Scarabaeinae	Scarabaeini	*Drepanopodus costatus*	206740	AY131794.1	AY131952.1	AY131612.1	-
Scarabaeoidea	Scarabaeidae	Scarabaeinae	Scarabaeini	*Drepanopodus proximus*	205293	-	AY965239.1	AF499694.1	-
Scarabaeoidea	Scarabaeidae	Scarabaeinae	Scarabaeini	*Kheper nigroaeneus*	205299	AY131795.1	AY131953.1	AF499695.1	-
Scarabaeoidea	Scarabaeidae	Scarabaeinae	Scarabaeini	*Pachysoma sp*. *dgi1*	206578	AY131797.1	AY131955.1	AY131615.1	-
Scarabaeoidea	Scarabaeidae	Scarabaeinae	Scarabaeini	*Scarabaeus galenus*	205312	AY131798.1	AY131956.1	AY131616.1	-
Scarabaeoidea	Scarabaeidae	Scarabaeinae	Scarabaeini	*Sceliages Scarabaeus brittoni*	205321	AY131800.1	AY131958.1	AY131618.1	-
Scarabaeoidea	Scarabaeidae	Scarabaeinae	Scarabaeini	*Sceliages Scarabaeus hippias*	205322	AY131801.1	AY131959.1	AY131619.1	-
Scarabaeoidea	Scarabaeidae	Scarabaeinae	Sisyphini	*Neosisyphus confrater*	206896	AY131802.1	AY131960.1	AY131620.1	-
Scarabaeoidea	Scarabaeidae	Scarabaeinae	Sisyphini	*Neosisyphus fortuitus*	206897	AY131803.1	AY131961.1	AY131621.1	-
Scarabaeoidea	Scarabaeidae	Scarabaeinae	Sisyphini	*Neosisyphus mirabilis*	206898	AY131804.1	AY131962.1	AY131622.1	-
Scarabaeoidea	Scarabaeidae	Scarabaeinae	Sisyphini	*Sisyphus crispatus*	206783	AY131805.1	AY131963.1	AY131624.1	-
Scarabaeoidea	Scarabaeidae	Scarabaeinae	Sisyphini	*Sisyphus gazanus*	206785	AY131807.1	AY131965.1	AY131626.1	-
Scarabaeoidea	Trogidae	-	-	***Omorgus sp*.**	COL740	-	KF801985	KF801820	Garradunga QLD
Scirtoidea	Scirtidae	-	-	***Accolabass sp*.**	COL497	KF802126	KF801962	KF801798	Mt Field NP TAS
Scirtoidea	Scirtidae	-	-	***Cyphon sp*.**	COL498	KF802127	KF801963	KF801799	Pieman River State Res TAS
Scirtoidea	Scirtidae	-	-	***Macrohelodes sp*.**	COL495	KF802124	KF801960	KF801796	Maydeena TAS
Scirtoidea	Scirtidae	-	-	***Pseudomicrocara sp*.**	COL496	KF802125	KF801961	KF801797	Lake St Clair NP TAS
Scirtoidea	Scirtidae	-	-	***Scirtes sp*.**	COL681	KF802143	KF801979	KF801815	Kuranda QLD
Staphylinoidea	Staphylinidae	-	-	***Actinus sp*.**	COL2262	KF802116	KF801952	KF801788	Garradunga QLD
Staphylinoidea	Staphylinidae	-	-	***Austrolophrum cribriceps***	COL2300	KF802117	KF801953	KF801789	Lake St Clair NP TAS
Staphylinoidea	Staphylinidae	-	-	***Eumecognathus sp*.**	COL2259	KF802113	KF801949	KF801785	Franklin-Gordon Wild Rivers NP TAS
Staphylinoidea	Staphylinidae	-	-	***Hesperus sp*.**	COL2261	KF802115	KF801951	KF801787	Wiangaree NSW
Staphylinoidea	Staphylinidae	-	-	***Leucocraspedum sp*.**	COL2260	KF802114	KF801950	KF801786	Cradle Mt NP TAS

Bold represent newly generated sequences, non-bold represent sequences from GenBank with TaxonomyID numbers. Collection locality is provided for samples collected for this study.

### DNA amplification and gene sequencing

DNA was extracted from the head and thorax of specimens using a QIAGEN DNeasy tissue kit as per manufacturer protocols. Three mitochondrial genes, *16S* rRNA, *12S* rRNA and cytochrome *c* oxidase subunit I (*COI*), and the nuclear gene, *28S* (LSU) rRNA, were amplified using primer pairs and amplification protocols as per Gunter et al. [23]. PCR products were purified using EXOSAP-it (Affymetrix). Cycle sequencing reactions were performed using the BigDye Terminator v1.1 Cycle Sequencing Kit, the products of which were purified by alcohol precipitation and sent to the John Curtin School of Medical Research, Australian National University (ANU-JCSMR) for sequencing. Sequences were edited using Sequencher (v4.5; Gene Codes Corporation, Ann Arbor, MI, U.S.A.). Bidirectional sequences were aligned to form contigs and edited using Sequencher v. 4.5.

### Multiple alignment and phylogenetic analysis

Once additional scarabaeoid sequences were downloaded from GenBank, sequences of each of the three fragments were aligned separately using default parameters of MAFFT [24] and Muscle [25](as implemented in Geneious ver. 5.6 [26]. Genes with limited or no length variability (*28S*, *COI*) were aligned with MAAFT as it produced a more reliable alignment (i.e. higher percentage pairwise identity and fewer inferred indels) than the MUSCLE alignments. The *16S-tRNA-Val-12S* amplicon was aligned as three individual genes, *12S* and *tRNA-Val* using MAFFT and *16S* using MUSCLE as the high degree of length variability in this gene reduced reliability of MAFFT alignments. Each alignment was edited by eye before concatenation using Geneious into a final dataset spanning 4,584nt. Almost all taxa within the analysis contain data from at least three genes (96.8%) and 91.2% of taxa (n = 413) had complete gene coverage.

PartitionFinder [27] was used to determine the best partitioning strategy and nucleotide substitution models for phylogenetic inference. The optimal partitioning scheme divided the data into six partitions, separating each of the RNA genes except *tRNA Val* and *12S*, and further subdividing *COI* by codon position. Bayesian Inference was conducted using MrBayes 3.2.1 [28–29]. Each analysis consisted of 30 million generations with a random starting tree, and two simultaneous runs with four Markov chains sampled every 1000 generations were conducted with unlinked partitions. Stationarity in MCM chains was determined in Tracer [30] and burn-in was set appropriately. A majority-rule consensus tree was obtained from the two combined runs to establish the posterior probabilities of clades. Maximum-likelihood analyses were performed using the RAxML [31] on the CIPRES portal [32] and the same partitioning strategies.

### Fossil Selection (Table 2)

**Table 2:** Calibration points used for the estimation of the divergence times.

Node	Clade	Type	Site	Species	Reference	Age	Analyses						
							CS1	CS2	CS3	CSi	CSii	CSiii	CSiv
A	Polyphaga	Mol.	NA	NA	Hunt et al. [8]	268–273 [8]	min-max	min-max	min-max				min-max
B	Bolboceratinae^1^	Fos.	Lower Cretaceous, Baissa, Zaza formation Russia^#^	*Cretobolbus rohdendorfi*	Nikolajev [60]	>130 [33] (129.4–139.8) [125]		min-max	min-max				
C	Geotrupinae^1^	Fos.	Lower Cretaceous, Baissa, Zaza formation Russia^#^	*Cretogeotrupes convexus*	Nikolajev [116]	>130 [33] (129.4–139.8) [125]		min-max	min-max				
D	Scarabaeidae	Fos.	Jurassic, Karatau, Kazakhstan	*Juraclopus rohdendorfi*	Nikolajev [62]	152–158 [33]			min-max				
E	Pleurosticti	Fos.	Jurassic, Karatau, Kazakhstan	*Juraclopus rohdendorfi*	Nikolajev [62]	152–158 [33]		min-max					
F	Melolonthinae	Fos.	Lower Cretaceous, Baissa, Zaza formation Russia^#^	*Cretomelolontha transbaikalixa*	Nikolajev [60]	>130 [33] (129.4–139.8) [125]		min	min				
G	Scarabaeinae	Fos.	Upper Cretaceous, Lanxi, China	*Prionocephale deplanate*	Krell [61]	83.5–92 [33]		min	min	min-max	min-max	min	min
H	Lucanidae	Fos.	Upper Jurrasic Shara-Teg, Mongolia	*Paralucanus mesozoicus*	Nikolajev, [53]	145.5–150.8 [18]				min-max	min-max	min-max	min-max
I	Trogidae	Fos.	Lower Cretaceous, Baissa, Russia	*Trox sibericus*	Nikolajev, [54]	140.2–145.5 [18]				min-max	min-max	min-max	min-max
J	Hyborsoridae	Fos.	Jurassic, Karatau-Mikhailovka, Kazakhstan	*Protohybosorus grandissimus*	Nikolajev, [55]	155.7–164.7 [18]				min-max	min-max	min-max	min-max
K	Glaphyridae	Fos.	Lower Cretaceous, Baissa, Russia	*Cretoglaphyrus* spp.	Nikolajev, [117]	140.2–145.5 [18]				min-max	min-max	min-max	min-max
L	Aphodiinae	Fos.	Upper Paleocene, Menat, France	*Aphodius charauxi*	Piton, [118]; Vincent et al. [119]	55.8–58.7 [18]				min-max			
M	Aphodius	Fos.	Oligocene, Florissant, USA	*Aphodius aboriginalis*	Wickham et al. [120]	33.9–37.2 [18]				min-max			
N	Nearctic Sericini	Fos.	Oligocene, Florissant, USA	*Serica* spp.	Krell, [60]	33.9–37.2 [18]				min-max			
O	Rhizotrogini	Fos.	Eocene, White River, Green River Formation, USA	*Phyllophaga avus*	Cockerell, [121]	46.2–50.3 [18]				min-max			
P	Cetoniinae	Fos.	Mid Eocene, Eckfelder Maar, Germany	Cetoniinae undescribed	Wappler, [122]	40.4–48.6 [18]				min-max			
Q	Anomalini	Fos.	Oligocene, Florissant, USA	*Anomala scudderi*	Wickham, [123]	33.9–37.2 [18]				min-max			
R	Adoretini	Fos.	Miocene, Shanwang, China	*Adoretus* spp.	Krell [60]	11.6–16.0 [18]				min-max			
S	Dynastinae	Fos.	Mid Eocene, Clarno Formation, Oregon, USA	*Oryctoantiquus borealis*	Ratcliffe et al. [124]	37.2–18.6 [18]				min-max			

Type of calibration refers to the age estimated from either a molecular estimate (Mol.) or from a fossil (Fos.) and listed as Ma. Analyses using calibration points are highlighted, “min-max” represent clades where both minimum and maximum age constraints are applied, “min” represents only minimum age constraints, empty cells represent a constraint is not applied. Calibration scheme as follows CS1: molecular constraint only; CS2-3 molecular constraint plus selected fossil constraints with *Juraclopus rohdendorfi* placed at either the crown (CS2) or stem (CS3) of the Pleurosticti clade; CSi-CSiv: fossil selections based of previous study CSi: fossil constraints only, hard bounds applied to all fossils; CSii: fossil constraints only, but limited to only Cretaceous fossils, hard bounds applied; CSiii: fossil constraints only, limited to only Cretaceous fossils, hard bounds only applied to outgroup taxa while ingroup taxa constrained only by minimum age; Civ: replicate of CSiii with molecular age constraint also applied.

^#^ Baissa formation is noted as being “probably pre-Barremian” and most paleoentomologists date the Zaza formation as Valanginian—Hauterivian

^1^ Bolboceratinae is currently recognized as a subfamily of Geotrupidae, accordingly the fossils listed as “Bolboceratidae” and “Geotrupinae” are placed at these subfamily levels.

For age calibration, fossils and applied age constraints were selected based on recent reviews of the scarabaeoid fossil literature and relied heavily on the reviews of Krell [33–34] that critiqued the reliability of taxonomic assignments made in initial fossil descriptions. The oldest scarabaeoid fossil, *Alloioscarabaeus cheni*, was not used as a fossil calibration point as it represents an extinct family, likely belonging to a primitive stem lineage [13], however, this fossil was used to cross-validate our results. The oldest unequivocal Scarabaeidae fossil, *Juraclopus rohdendorfi* cannot be unequivocally assigned to its proposed subfamily Aclopinae (as discussed in Bai et al. [35]), therefore both crown and stem positions for this fossil were tested. The phylogenetic position of Aclopinae is currently unknown and only *Phaenognatha*, which is currently classified in the Aclopinae (see Ocampo & Mondaca, [36]), is included in this analysis where it falls within the phytophagous clade. It has been suggested Phaenognathini may be a melolonthine tribe [22] but no classification changes have been made. Until *Aclopus*, the type genus, is sequenced the relationship between *Aclopus* and *Phaenognatha* will remain unclear and so we conservatively constrained the fossil within the phytophagous clade, instead of placing it on the *Phaenognatha* node. Accordingly, we tested two fossil calibration strategies for placement of the *Juraclopus* fossil: as either a crown or stem member of the phytophagous clade. For both fossil calibrated schemes (CS2: *Juraclopus* in the phytophagous crown-group, CS3: *Juraclopus* in the phytophagous stem-group), the root age of Polyphaga was constrained at 268–273 Ma on the basis age estimate of Polyphaga from Hunt et al. [8], which was deemed to be more consistent with the scarabaeoid fossil record than the estimate from McKenna et al. [9] (see above).

These fossil calibration points were assessed against the fossil calibrations used by Ahrens et al. [18]. The rationale behind testing alternative calibration schemes was to determine if the differences in age estimates between the present study and Ahrens et al. [18] were due to (i) the dataset and analysis methods, (ii) fossil calibration and priors, (iii) fossil selection, or (iv) influence of a molecular calibration point. The seven calibration schemes are summarized in Table 2 along with the details of the fossils used and the nodes constrained.

### Calibration schemes

To explore the above divergence dating hypotheses, the following calibrations using the fossils selected by Ahrens et al. [18] were applied to our 445 taxon, 4 gene dataset:

1. hard bounds (minimum and maximum age) for all Ahrens fossil calibrations (excluding *Aegialia* which was not present in our taxon sampling) (CSi). Minimum and maximum ages reflect the upper and lower bounds of the dated fossil layer interval respectively (see Table 2). A direct comparison can be made to the results of Ahrens et al. [18] based on their 146 species, 4 gene data set providing a test of effects of dataset or analysis method. Although hard bounds are stricter than the exponential priors used in Ahrens et al. [18], these comparison analyses serve to demonstrate the differences due to analysis calibration and not taxon or gene sampling
2. To test the effect of hard bounds on fossil calibrations, in particularly on Cenozoic fossils, we compare the results of CSi to the following two calibrations: CSii) using only Cretaceous fossils as calibrations with hard bounds (minimum and maximum age); CSiii) Cretaceous fossils only, hard bounds (minimum and maximum age) except for ingroup fossils which were only constrained with a minimum age.
3. To test if fossil selection is responsible for differences in age estimates, the results from the CS2 and CS3 can be compared to CSiii which all do not have Cretaceous or Cenozoic upper bounds applied within the ingroup.
4. The results from the program r8s are known to be influenced by the oldest calibration applied. All analyses of Ahrens et al. [18] only include fossil calibrations, the oldest dated from the Middle Jurassic. To determine if using the molecular age of the Polyphaga as a calibration point significantly influenced the estimated ages in CS2-CS3, we compare results of CSiii and CSiv which are identical except CSiv includes the molecular calibration point for the Polyphaga. Additionally, the tree was calibrated only using the root age of Polyphaga, 268–273 Ma as estimated in Hunt et al. [8], to provide a best guess estimate independent from fossil calibrations (CS1).

### Divergence time estimates

Divergence date estimates were calculated for all seven calibration schemes using a penalized likelihood (PL) method and we further tested four of these schemes (CS2, CS3, CSi and CSiii) under a Bayesian scenario using MCMCTree.

PL divergence estimates were calculated with a truncated Newton algorithm using the software r8s, ver. 1.7.1 [37]. The consensus tree with branch lengths obtained from Bayesian analyses was used as a fixed input tree. The optimal level of rate smoothing was determined by cross-validation and a smoothing parameter was set accordingly. The use of PL in divergence dating analyses has been criticized as not as robust as newer, Bayesian methods. Bayesian divergence dating programs like BEAST [38], Multidivtime [39], and MCMCTree [40] have advantages such as being able to deal with uncertainties in topology and branch length and can allow for different types of prior distributions. Validation of our divergence estimates obtained from r8s was initially attempted in BEAST but given the size of the data set BEAST struggled to identify an appropriate seed tree and the analyses were aborted because the initial state had zero probability. This can occur if “the log likelihood of the tree is -Inf, [which] may be because the initial, random tree is so large that it has an extremely bad likelihood which is being rounded to zero” [38]. Even when a fully bifurcating tree was used as input, BEAST continued to reject the log likelihood of the tree. Given the failure of the analysis to launch in BEAST, validation was next attempted in MCMCTree implemented in the PAML package [40]. MCMCTree has been shown to handle analyses with much larger numbers of taxa than either BEAST or Multidivtime while still producing comparable time estimates [41–42]. An estimation of substitution rates using a GTR model was calculated from the sequence alignment and input topology (the same output tree from the Bayesian analysis used in PL) with BASEML in the PAML package and used to adjust the rgene_gamma parameter accordingly in the control file. The Sigma2_gamma parameter was also set appropriately according to the estimated age between the tip and root of the tree (using the molecular divergence date estimated in Hunt et al. [8]). The gradient and hessian of branch lengths were calculated in MCMCTree and used in the final estimation of divergence times by Bayesian analysis using a modified fully bifurcating version of the original tree. The MCMC parameters were set so the first 50,000 iterations were discarded as burnin, and then sampled every 50 iterations until it had gathered 10,000 samples (burnin = 50,000 sampfreq = 50 nsample = 10,000), in total running for 550,000 iterations. All analyses were made with GTR as the model of substitution, with a flexible birth-death prior used (BDparas = 2 2 0.1). More uniform priors were also tested (BDparas = 1 1 0) but did not affect the posterior estimates significantly. Fine tuning parameters were set at 0.06, 0.5, 0.006, 0.12 and 0.4 for time, substitution rate, mixing, and substitution parameters, respectively. Acceptance proportions were checked after fine-tuning to ensure they ranged around ~30% and were always in the most reliable range of between 20–40% for MCMC analyses.

### Diversification and speciation estimates

The chronograms resulting from analyses were used to generate lineage through time (LTT) plots using the packages gieger, ape, TreeSim and laser [43–46] in R. LTT plots were generated for specific clades through exclusion of the remaining taxa and pruned in Mesquite [47]. For the purpose of the generalist versus specialist angiosperm feeding analysis the tribe Hopliini is included within the Melolonthinae (generalist, leaf feeding) LTT on the basis of current classification and while Hopliini feed on leaves and pollen [10], pollen feeding has been demonstrated to be a derived trait [11]. LTTs were plotted in both linear and log scales as comparison of rates between groups with differing number of taxa is easier to visualize with the linear scales.

Clade-wide rates of diversification were estimated using the method of moments (MoM) detailed by Magallón & Sanderson [48] with three relative extinction rates (ε = 0/0.5/0.9). Species richness data for each clade is taken from Scholtz & Grebennikov [10] and the estimated age of each clade from PL analysis CS3. Relative diversification rates between clades did not differ depending on which of the calibration schemes were used. To examine speciation, extinction, net diversification and net extinction rates in a Bayesian framework, as well as potential mass extinctions, some of the resulting chronograms were assessed using the package TESS in R [49]. Given that two thirds of genera were represented by a single species, we excluded the most recently diverging, species level sampling (45 or 50Ma depending on the analysis) to result in a topology with approximately 120–131 tips (representing ~2/3 tip sampling). A sampling fraction (*rho*) was then calculated based on the number of tips and nodes of each of the topologies at either 45 or 50Ma (depending on the analysis) based on the predicted diversity calculated using MoM detailed above (i.e. with no extinction Scarabaeinae richness was estimated as 1619 or 1457 at 45 and 50 Ma respectively). A summary of the analyses, excluded tips and sampling fractions are listed in S1 Table. A series of preliminary TESS / CoMET analyses was performed for each topology (with diversification set as 0.068635 for Scarabaeinae or 0.066875 for the Pleurosticti, as predicted by MoM), and held constant and extinction set at 0), to estimate the marginal posterior probability densities for the diversification-rate hyperpriors *mu* and *sigma* for both initial speciation and extinction rates for final TESS analyses. All TESS and CoMET analyses were run under the following conditions with unique combinations of prior settings for *mu*, *sigma* and *rho*: number of expected rate changes = log(2), expected survival probability of 25% (beta prior with shape parameters α = 0.25 and β = 0.75) consistent with prior estimated loss of 70–75% of contemporaneous terrestrial species [50–51], number of mass extinctions = 1, maximum number of MCMC iterations = 100000 with 4 chains, burnin = 50000, thinning each chain by sampling every 10^th^ state. MCMC simulations were terminated when the effective sample size reached 500 and we explored the maximum number of iterations and thinning to ensure ESS would reach 500 and the burnin and thinning setting were appropriate.

## Results

### Phylogeny of the Scarabaeoidea (Fig 1 and S1 Fig)

**Fig 1:**
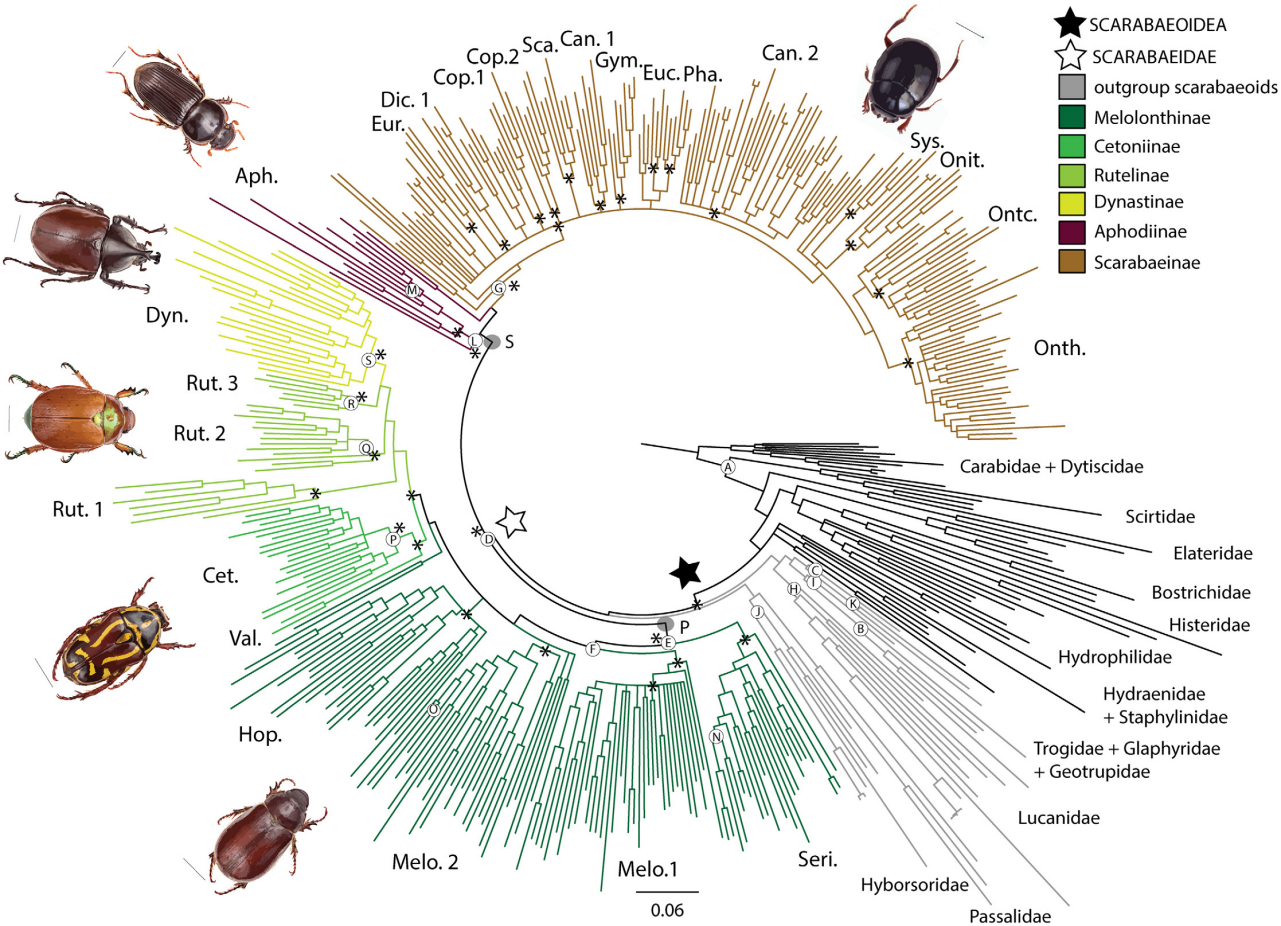
Bayesian Phylogeny of scarab beetles (Scarabaeidae). Taxa color-coded by scarab subfamily, with outgroups in grey (superfamily) and black (other beetles). Grey circles indicate polyphagous (P) and saprophagous (S) lifestyles. White circles represent the node priors A-S as per Table 2.* represents nodes for which divergence dates are inferred. See also S1 Fig for posterior probability and terminal names.

Scarabaeoidea was recovered as a strongly supported monophyletic lineage (BS = 0.97) in a strongly supported lineage containing the Hydrophiloidea, Staphylinoidea and Scarabaeoidea (PP = 1). A weakly supported sister relationship between Staphylinoidea and Scarabaeoidea was also recovered (0.5). Four main lineages were recovered within Scarabaeoidea (i) Geotrupidae + Glaphyridae + Trogidae (PP = 0.76); (ii) Lucanidae (PP = 0.97); (iii) Passalidae + Hyborsoridae (PP = 0.87), (iv) Scarabaeidae (BS = 0.78) (see Fig 1). A sister relationship was recovered between Geotrupidae + Glaphyridae + Trogidae clade and Lucanidae (PP = 0.71) and very weakly supported between Passalidae + Hyborsoridae and Scarabaeidae (PP = 0.5). The family Geotrupidae was not recovered as monophyletic, instead *Geotrupes* (Geotrupidae: Geotrupinae) formed a closer relationship with Glaphyridae (PP = 0.77) than with the monophyletic lineage containing four bolboceratine genera (Geotrupidae: Bolboceratinae) (PP = 1) that formed a sister relationship with Trogidae (PP = 0.98). The families Lucanidae, Passalidae, Hyborsoridae and Scarabaeidae were all recovered as supported monophyletic lineages (PP = 0.71, 1, 0.89 and 0.78 respectively). Within the lucanid clade, only the subfamily Lucaninae was represented by more than one species (n = 9) the majority forming a strongly supported relationship (PP = 1) to the exclusion of *Platycerus kawadai*. Within the family Hyborsoridae, the subfamilies Hyborsorinae and Ceratocanthinae both were represented by multiple species and formed monophyletic lineages; a strongly supported sister relationship was recovered between Ceratocanthinae and Liparochrinae (PP = 1). Scarabaeidae was divided into two lineages the first containing the Pleurosticti or phytophagous scarab subfamilies (Melolonthinae, Cetoniinae, Rutelinae and Dynastinae PP = 0.89) and the second containing the saprophagous subfamilies (Aphodiinae and Scarabaeinae PP = 0.81).

Within the Pleurosticti clade, Melolonthinae formed a grade of early branching lineages while a close relationship was formed between the Cetoniinae, Rutelinae and Dynastinae (referred to as CRD clade) (PP = 0.89) (see Fig 1). A monophyletic Cetoniinae (PP = 0.99) was the earliest branching clade within the CRD clade and was divided into three lineages Valgiini, Cetoniini + Goliathini, and Schizorhini+ Stenotarsiini. Rutelinae was paraphyletic and formed a grade of lineages containing the monophyletic Anoplognathini (PP = 1), Rutelini + Anomalini (PP = 1), and Adoretini (PP = 1). Dynastinae formed a monophyletic lineage (PP = 1), however, the tribes Orycroderini, Pentodontini and Phileurini were paraphyletic while Cyclocephalini and Dynastini were each only represented by a single genus.

Within the Saprophagous clade, the aphodiines formed the earliest branching lineages representing Rhyparini (*Rhyparus* sp. only), Eupariini + Proctophanini + Psammodiini (PP = 0.84), and Aphodiini (PP = 1). Scarabaeinae was recovered as monophyletic (PP = 0.97) with *Sarophorus* + *Coptorhina*, and *Odontoloma+ Dicranocara* forming the earliest branching lineages followed by a comb containing most of the tribal diversity (PP = 0.97). Many monophyletic lineages representing the tribes were recovered while the tribes Coprini and Canthonini were recovered as paraphyletic (see Fig 1). Overall the topology is largely congruent with the traditional relationships based on morphology and recovers the highest molecular support for a monophyletic Scarabaeidae observed to date (PP = 0.78).

### Divergence time estimates

**Fig 2:**
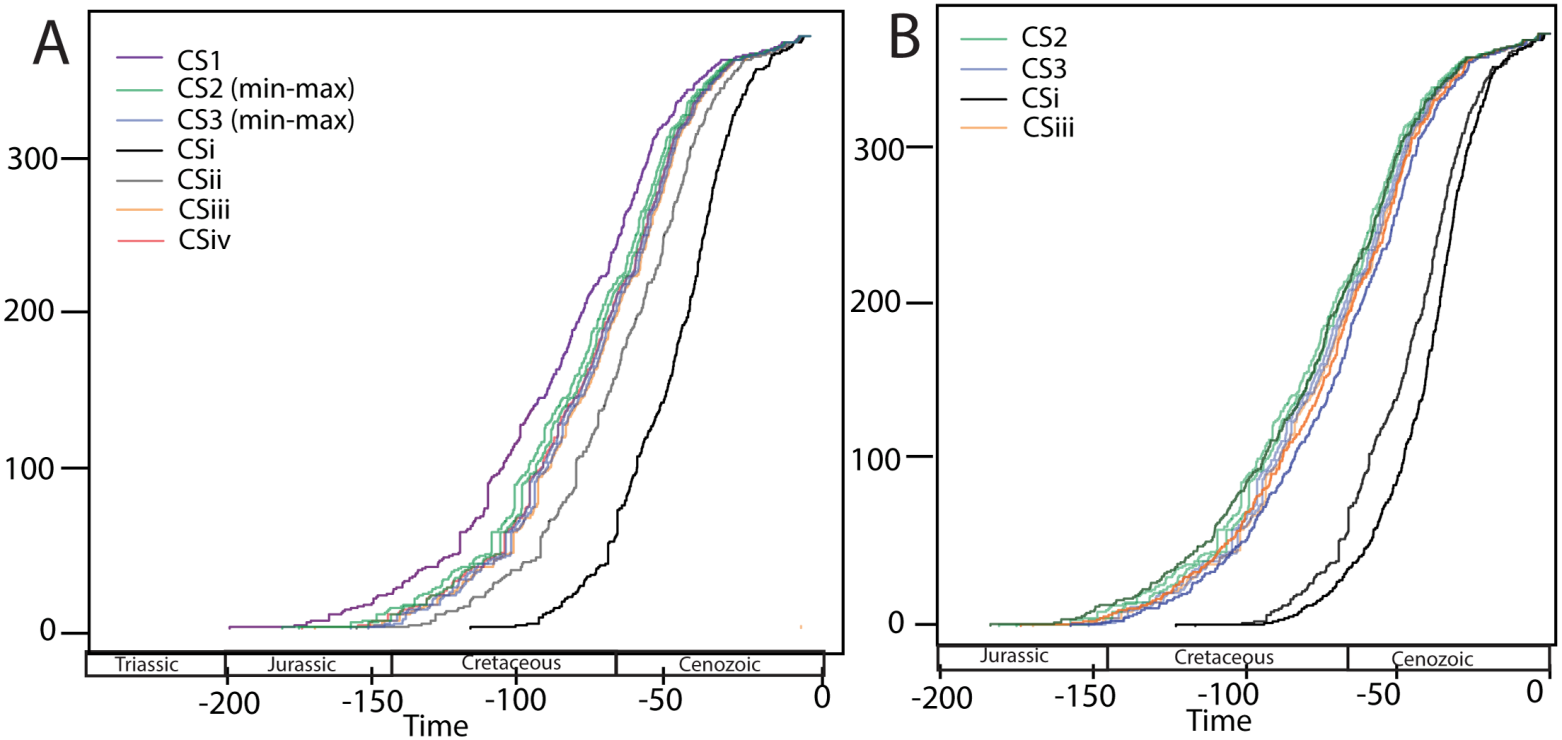
Comparison of accumulation of Scarabaeidae in different divergence dating analyses. (A) Penalized Likelihood (PL) method estimated in r8s using seven different calibration schemes (B) Bayesian methods estimated in MCMCTree using four different calibration schemes (bold) and shadowed by their corresponding PL estimate (pale).

We found a high degree of congruence between divergence dates estimated by penalized likelihood (PL) or Bayesian methods, and between similarly constrained analysis priors, independent of fossil choice or data set (Table 3). Fig 2 plots the accumulation of ingroup taxa (Scarabaeidae) using seven different calibration schemes analysed by PL and four calibration schemes analysed by Bayesian methods and highlights that the results of analyses CS2, CS3, CSiii and CSiv are highly congruent.

**Table 3:** Predicted ages of scarab lineages using different fossil calibrations and divergence dating programs.

	CS1	CS2	CS2	CS3	CS3	CSi	CSi	CSii	CSiii	CSiii	CSiv
Dating method	r8s	r8s	MCMCTree	r8s	MCMCTree	r8s	MCMCTree	r8s	r8s	MCMCTree	r8s
SCARABAOIDEA	216.61	199.43–202.14	202.6 (190.9–215)	191.94–194.38	187.18 (176.3–199.6)	169.26	164.75 (160.2–170.3)	176.4	185.66	195.08 (183.3–209.1)	189.67
SCARABAEIDAE	199.64	176.96–181.84	183.5 (168.5–187)	152–158	156.99 (154.0–158.3)	116.85	122.55 (99.2–156.7)	156.06	170.36	173.35 (162.6–185.7)	174.78
*Phytophagous clade* Pleurosticti	177.38	152–158	156.84 (153.6–158.2)	142.31–146.91	146.84 (141.0–151.8)	101.4	90.86 (83.6–99.4)	138.11	151.25	156.49 (145.8–168.4)	155.56
Sericini	153.56	133.75–138.29	143.79 (140.1–150.8)	125.31–129.32	125.6 (110.3–141.9)	88.39	74.42 (63.4–84.0)	119.19	130.37	130.14 (113.7–148)	134.54
Melolonthinae 1	150.81	131.75–136.06	148.77 (132.9–151.6)	123.8–127.27	133.81 (123.0–142.4)	86.22	81.32 (73.7–88.6)	116.84	128.23	141.47 (127.3–154.9)	132.02
Melolonthinae 1b	120.43	106.32–109.62	124.2 (115.5–132.3)	100.42–102.89	115.44 (106.7–124.0)	69.53	69.96 (62.5–77.4)	92.77	102.04	121.19 (110.5–133.1)	105.16
Melolonthinae 2	160.73	140.33–145.06	146.64 (139.2–152.7)	131.95–135.7	136.55 (128.6–143.7)	90.88	81.42 (75.3–85.6)	125.2	137.16	144.53 (132.8–156.7)	141.12
Hoplini	150.24	131.92–136.96	137.11 (126.2–147.8)	124.18–127.54	127.92 (116.2–138.7)	84.43	76.3 (68.8–83.6)	117.05	128.25	133.84 (121.2–148.1)	131.96
*Cetoniinae+ Rutelinae +Dynastinae*	155.7	136.27–140.74	137.28 (129.2–145)	128.23–131.79	128.1 (119.9–136.1)	84.32	74.6 (68.5–80.9)	121.36	132.93	134.24 (123.9–145.9)	136.76
Cetoniinae + Valgini	142.35	124.86–128.84	126.28 (115.3–136.7)	117.57–120.75	117.73 (107.2–128.3)	73.57	67.45 (57.9–75.6)	110.8	121.42	123.55 (111.5–139.9)	124.95
Cetoniinae- Valgini	115.22	101.42–104.5	104.26 (92.8–115.3)	95.59–98.07	96.8 (86.2–107.8)	48.6	48.01 (45.9–49.3)	89.45	98.12	100.67 (88.5–113.1)	101.02
Rutelinae1- Anoplognathini	115.02	101.35–104.46	102.4 (90.5–113.3)	95.59–98.09	95.04 (83.9–106.0)	46.26	55.29 (47.8–63)	89.99	98.53	99.69 (87.5–112.3)	101.33
Rutelinae 2- Rutelini + Anomalini	115.61	101.9–104.95	105.96 (92.6–118.3)	96.1–98.55	98.3 (85.3–110.5)	45.6	49.97 (40.8–59.8)	90.09	98.54	102.72 (88.1–17.2)	101.43
Rutelinae 2b- Anomalini	110.36	97.28–100.18	100.01 (86.5–112.9)	91.74–94.08	92.81 (97.8–105.3	37.2	36.66 (35.0–37.4)	85.76	94.04	97.02 (82.6–111.4)	96.8
Rutelinae 3-Adoretini	74.6	65.18–67.75	69.77 (53.7–87.1)	62.09–63.65	65.26 (50.1–82.1)	16	15.94 (15.2–16.6)	57.95	63.56	66.89 (51.4–84.3)	65.42
Dynastinae	117.92	104.22–107.28	105.79 (97.1–114.9)	98.4–100.87	98.52 (89.8–107.1)	48.6	48.5 (46.5–50.1)	92.19	100.89	102.62 (92.5–113.6)	103.78
*Saprophagous clade*	173.17	155.09–158.84	154.6 (148.6–166.2)	139.37–143.81	139.48 (129.7–148.6)	91.94	93.63 (87.3–103)	130.54	147.25	147.31 (134.1–161.1)	151.52
Aphodiinae	160.04	143.54–146.95	147.1 (135.3–157.2)	129.92–133.85	132.71 (121.8–142.3)	88.12	89.01 (84.5–96.2)	121.01	136.11	138.53 (124.9–152.9)	140.09
Scarabaeinae	143.3	128.6–131.58	142.49 (130.3–152.4)	118.76–121.85	127.83 (116.5–138.8)	83.5	85.6 (83.6–90.4)	**92**	121.26	130.76 (119.4–146.9)	124.99
Scarabaeinae b	110.77	99.54–101.83	128.82 (120.1–138.4)	93.18–95.3	117.82 (109.0–127.2)	66.31	77.62 (71.4–83.7)	80.54	93.58	122.4 (111.9–134.8)	96.54
Eurysternini	89.29	73.94–75.63	76.52 (60.3–93.2)	69.24–71.69	71.33 (56.3–87.0)	49.3	44.13 (34.9–55.0)	60.1	69.52	73.98 (58.1–90.8)	77.7
Dichotomini	103.82	93.21–95.33	79.24 (64.5–93.4)	87.26–90.35	73.28 (59.6–86.7)	57.82	45.25 (36.2–53.9)	70.46	87.62	76.71 (62.1–91.4)	71.71
Coprini1	97.04	87.1–89.09	88.98 (79.6–110.8)	81.54–84.43	82.7 (67.0–97.6)	58.01	49.43 (45.3–66.0)	70.66	81.87	85.27 (68.7–102)	84.46
Coprini 2	101.83	91.41–93.5	95.94 (89.9–115.9)	85.58–88.61	88.74 (73.4–103)	60.91	55.77 (38.8–59.8)	74.08	85.94	91.87 (75.5–107.4)	88.65
Scarabaeini	78.85	70.79–72.4	82.27 (65.7–99.6)	66.28–68.63	75.77 (60.2–91.4)	47.17	47.82 (37.4–58.8)	57.59	66.54	78.5 (62.3–95.8)	68.64
Canthonini1	102.25	91.27–93.87	85.76 (72.0–99.1)	85.91–88.96	79.56 (66.9–91.9)	61.12	58.83 (45.7–62.7)	74.46	86.26	83.45 (70.3–97.2)	89
Gymnopleurini	89.98	80.55–82.5	70.93 (56.6–85.4)	75.39–78.07	65.37 (52.5–79.1)	53.57	41.04 (33.7–49.2)	65.45	75.68	67.92 (53.0–81.7)	78.09
Eucranini	61.34	54.95–56.24	53.72 (39.8–68.4)	51.43–53.27	49.25 (36.3–63.2)	36.46	32.72 (22.2–41.3)	44.9	51.61	51.92 (38.4–66.7)	53.27
Phaenini	57.93	51.89–53.1	51.03 (37.9–64.6)	48.55–50.3	47.18 (35.6–60.6)	34.43	32.82 (23.4–41.1)	42.32	48.72	48.51 (36.4–62.2)	50.29
Canthonini 2	99.72	89.45–91.5	105.08 (96.2–113.8)	83.74–86.72	96.72 (88.4–105.1)	59.52	60.86 (54.6–67.7)	72.94	84.05	100.62 (91.2–115.3)	86.72
New Caledonian “Epilissini”	52.98	54.45–55.89	55.79 (42.5–70.2)	52.02–52.99	51.75(39.5–65.5)	36.25	35.95 (26.5–44.0)	44.61	51.33	53.41 (41–68)	52.98
“Epilissini”+ *Diorygopyx*	96.40	79.38–81.46	82.97 (68.7–96.9)	75.87–77.27	76.35 (63.2–89.4)	52.96	47.76 (39.7–56.0)	65.03	74.87	79.48 (65.3–93.7)	77.27
Sysiphini	59.59	53.62–54.82	53.58 (38.1–68.6)	50.29–52.03	49.01 (36.0–63.3)	35.98	34.29 (24.0–44.1)	43.87	50.47	50.69 (36.9–66.4)	52.02
Onitini	86.33	77.68–79.41	84.1 (70.9–96.9)	72.82–75.35	77.64 (65.5–89.8)	52.06	48.63 (40.8–56.5)	63.37	73.1	79.57 (66.2–93.4)	75.37
Oniticellini	85.75	76.97–76.68	84.13 (74.4–93.7)	72.2–74.69	77.45 (68.4–86.7)	54.2	48.06 (42.4–54.)	66.02	76.02	80.08 (70.3–90.4)	78.37
Madagascan Oniticellini (“Helictopleurini”)	66.23*	59.43–60.92*	66.17 (54.1–78)	57.91–56.89*	61.16 (50.1–72.1)	40.14*	39.67(33.9–46.6)	48.98*	56.18*	64.31 (53.1–75.7)	57.91*
Onthophagini	81.11	72.97–74.6	78.29 (70.3–87.1)	68.41–70.79	72.72 (64.7–81)	48.91	47.13 (42.4–52.0)	59.75	68.66	75.75 (67.0–85.3)	70.79

Four of these calibration schemes were further examined using Bayesian methods in MCMCTree.

* In part

#### (i) Differences between Penalised Likelihood and Bayesian analyses

MCMCTree and r8s performed slightly differently due to the constrained topology i.e. MCMCTree requires a fully bifurcating tree as final input for age estimation. Fig 2B plots the accumulation of ingroup taxa (Scarabaeidae) of r8s and MCMCTree analyses and highlights the differences due to tree topology with Bayesian methods producing a smoother curve because of the absence of polytomies and diverging earlier yet accumulating more slowly than PL. Differences in the age estimates between r8s and MCMCTree are summarised in Table 3 and S2 and S3 Figs. In general age estimates from MCMCTree analyses were older than those estimated in r8s but not systematically so. The most marked difference was the age of the clade that corresponds to the large radiation of dung feeding tribes, which was unresolved in the underlying phylogenetic analysis. Interestingly the ages predicted for the other significant unresolved comb in the Melolonthine lineage was also significantly older in the MCMCTree analysis. This suggests that forced input of a fully bifurcating tree is not ideal, biasing results towards older age estimates. However, age estimates on resolved clades were in very close congruence between both methods, adding confidence to the overall performance of our PL analysis.

#### (ii) Differences between data set

To test if our dataset, alignment or dating methods significantly influenced the results, age estimates from CSi were directly compared to Ahrens et al. [18] as this calibration scheme was designed to replicate their constraints used in run 1 and 2. Results for clades ages listed in Ahrens et al. [18] are compared in Table 4. Overall results from CSi are similar to run 1 and 2 of Ahrens et al. [18], highlighting that our 450 taxa dataset analysed using r8s and MCMCTree produces comparable age estimates to Ahrens et al. [18] 146 taxa dataset analysed in BEAST, when the same fossil calibrations are used.

**Table 4:** Comparison of age estimates from CSi, CSiii and run1-4 of Ahrens et al. [18] for selected ingroup clades.

Taxon	Csi r8s	CSi MCMC Trees	Run1 BEAST	Run2 BEAST	Run3 BEAST	Run4 BEAST	Csiii r8s	CSiii MCMC Trees
Scarabaeidae	116.85	122.55 (99.2–156.7)	NA	NA	NA	NA	170.36	173.35 (162.6–185.7)
Aphodiinae	**88.12**	**89.01 (84.5–96.2)**	**65.5 (57.3–73.3)**	**69.2 (58.4–79.9)**	111.8 (94.8–129.7)	124.7 (108.0–142.7)	136.11	138.53 (124.9–152.9)
Scarabaeinae	**83.5**	**85.6 (83.6–90.4)**	**86.6 (83.5–93.1)**	**86.6 (83.5–92.2)**	**92.6 (83.5–103.9)**	**100.2 (86.7–114.8)**	121.26	130.76 (119.4–146.9)
Scarabaeinae dung feeding	66.31	77.62 (71.4–83.7)	72.7 (63.1–82.1)	72.6 (63.3–81.1)	78.7 (68.0–90.1)	85.5 (73.5–98.4)	93.58	122.4 (111.9–134.8)
Pleurosticti (crown)	101.4	90.86 (83.6–99.4)	113.6 (97.4–129.6)	112.2 (95.9–128.7)	119.1 (105.2–134.0)	128.1 (113.1–142.1)	151.25	156.49 (145.8–168.4)
Southern world Melolonthinae	86.22	81.32 (73.7–88.6)	77.4 (55.2–98.4)	82.0 (60.5–104.4)	88.7 (68.5–108.7)	95.8 (75.1–115.7)	128.23	141.47 (127.3–154.9)
Sericini	88.39	74.42 (63.4–84.0)	99.9 (85.5–114.4)	96.6 (81.2–111.3)	103.0 (89.9–116.8)	111.3 (97.4–124.7)	130.37	130.14 (113.7–148)
Cetoniinae*	**48.6**	**48.01 (45.9–49.3)**	**58.2 (45.0–71.8)**	**56.8 (42.6–69.9)**	**64.4 (51.1–77.1)**	**71.9 (57.4–86.4)**	98.12	100.67 (88.5–113.1)
Dynastinae	**48.6**	**48.5 (46.5–50.1)**	**47.0 (37.2–56.9)**	**46.0 (37.2–55.2)**	**51.9 (40.9–62.5)**	**57.6 (46.1–69.4)**	100.89	102.62 (92.5–113.6)

Bold cells indicate maximum age constraints were applied to the node for which estimate is listed.

* Members of the cetoniine Valgini are not included in the taxon sampling of comparative study so the fossil calibration was applied to the node representing Cetoniinae- Valgini and corresponding results for the age of Cetoniinae also reflects this node for comparative purposes.

#### (iii) Differences between fossil selection

Results from CSiii can be compared to CS2 and CS3 to examine the influence of fossil selection. These three calibration schemes use only Mesozoic fossil calibration points and only outgroup taxa are constrained with maximum bounds, providing an opportunity to cross-validate fossil selection. Cross-validation of fossils is discussed in further detail below but in general, regardless of fossil set, when analyses are constrained using similar bounds the results are highly congruent for the majority of clades in both PL and Bayesian age estimates, ruling out major conflict between selected fossil sets (see Table 3 and Fig 2).

#### (iv) Use of a molecular age calibration point

As a test independent from fossil calibrations, CS1 was only constrained by the age of the Polyphaga estimated by Hunt et al. [8]; the resulting age estimates were older than those recovered when calibrated with fossils. Although the fossil record provides a direct timescale for the appearance of taxa, it is incomplete particularly for hexapods which are represented by a relatively poor fossil record [52]. Given the poor fossil record of insects, it is likely that additional fossils from the earliest scarab lineages have yet to be discovered or remain unpreserved, therefore the estimates from CS1 are plausible if considering a gap in the fossil record (see Table 5). However, this analysis was conducted only as an upper most prediction from which we could cross-validate our fossil selection.

**Table 5:** Cross validation of divergence dates estimated in r8s.

Calibration node	CS1	CS2	CS3	CSi	CSii	CSiii	CSiv	Oldest fossil (age) (Reference)
Polyphaga	**268**	**258.44**	**258.01**	230.59	217.06	232.06	**268**	
Scarabaeoidea	216.61	199.43/202.14	191.94/194.38	173.18	181.97	192.39	197.89	*Alloioscarabaeus cheni* (165Ma) [13]
Bolboceratinae	137.11	**139**	**139**	105.69	113.87	119.47	121.85	*Cretobolbus rohdendorfi* (129.4–139.8) [60]
Geotrupinae	177.04	**139**	**139**	157.0	161.37	166.13	168.17	*Cretogeotrupes convexus* (129.4–139.8) [116]
Glaphyridae	123.38	99.29/99.50	98.92/99.07	**140**	**140**	**140**	**140**	*Cretoglaphyrus spp*. (145.5–140.2) [117]
Hyborsoridae	192.78	177.05/179.78	172.07/173.84	**128.14**	**131.48**	**139.31**	**139.74**	*Protohybosorus grandissimus* (145.5–140.2) [55]
Ceratocanthinae	100.11	91.88/93.39	89.28/90.38	72.36	80.08	85.72	86.93	*Mesoceratocanthus tuberculifrons* (122.1–129.7) [58]
Hyborsorinae	162.89	149.62/151.96	145.43/146.94	128.14	131.48	139.13	139.74	*Protohybosorus grandissimus* (145.5–140.2) [55]
Lucanidae	195.76	178.32/180.74	173.99/175.55	**150.8**	**150.8**	**150.8**	**150.8**	*Paralucanus mesozoicus* (145.5–150.8) [53]
Lucaninae	178.8	163.19/165.37	159.3/160.64	136.37	138.77	139.64	139.95	*Cretolucanus* spp. (100.5–113.0)[54]
Sister relationship of Lampriminae + Syndesinae	167.7	152.97/155.03	149.3/150.64	128.04	129.93	130.67	130.94	*Prosinodendron krell* (Syndesinae) (122.1–129.7) [126]
Scarabaeidae	199.64	176.96/181.84	**152/158**	116.85	156.02	170.36	174.78	*Juraclopus rohdendorfi* (152–158) [62]
Phytophagous clade/ Pleurosticti	177.38	**152–158**	142.31/146.91	101.4	138.11	151.25	155.56	*Juraclopus rohdendorfi* (152–158) [62]
Sericini	153.56	133.75/138.29	125.31/129.32	88.39	119.19	130.37	134.54	*Cretoserica latitibialis* (129.4–139.8) [60]
Nearctic Sericini	70.44	72.89/74.83	68.83/70.44	**37.2**	63.43	69.67	71.77	
Aphodiinae	160.04	143.54/146.95	129.92/133.85	**58.7**	121.01	136.11	140.09	*Cretaegialia aphodiiformis* (129.4–139.8) [127]
*Aphodius*	91.12	82.01/83.86	76.07/77.94	**42.72**	69.38	77.40	79.74	
Cetoniinae	115.22	101.42/104.5	95.59/98.07	**46.8**	89.45	98.12	101.02	
Anomalini	115.61	101.9/104.95	96.1/98.55	**37.2**	90.09	98.54	101.43	
Adoretini	74.6	65.18/67.75	62.09/63.65	**16**	57.95	63.56	65.42	
Dynastinae	117.92	104.22/107.28	98.4/100.87	**48.6**	92.19	100.89	103.78	
Melolonthinae	171.70	143.16–146.41	148.52–153.95	97.47	133.67	146.43	150.63	*Cretomelolontha transbaikalica* (140.2–145.5Ma) [60]
Rhizotrogini	83.70	73.98/76.12	69.78/71.94	**48.05**	65.35	71.56	73.63	
Scarabaeinae	143.3	**128.6–131.58**	**118.76–121.85**	**83.5**	**92**	**121.26**	**124.99**	*Prionocephale deplanate* (83.5–92) [128]

Analyses CS1-3 and CSi-iv represent different fossil sets for calibration. Bold text represents nodes constrained with minimum bounds only, bold and underlined represent constrained nodes with minimum and maximum bounds. Oldest known fossils only from the Mesozoic are listed.

To test the effect of using the molecular age estimate of the Polyphaga from Hunt et al. [8] as a bound on PL analyses, the calibration scheme CSiv was designed to replicate CSiii with the addition of the Polyphaga being bound by the molecular age estimate above. The results demonstrate minimal differences in age estimates in the ingroup but age estimates were always older with the Polyphaga constrained (see Table 6). For example the estimated ages in analyses CSiii and CSiv for Scarabaeoidea were both in the Sinemurian (192.39Ma or 197.89Ma respectively), the Scarabaeidae in the Aalenian/Toarcian (170.36Ma or 174.78 Ma); the phytophagous clade in the Tithonian/Kimmeridgian (151.25Ma or 155.56 Ma) and the saprophagous clade in the Tithonian (147.25Ma or 151.52Ma). These results suggest that applying this bound at the root of the Polyphaga in CS2 and CS3 would have minimal effect on age estimations, allowing for comparison between calibration schemes.

**Table 6:** Crown group divergence times.

	General adult diet	CS1	CS2	CS3	CSi	CSii	CSiii	Civ
SCARABAOIDEA		216.61	199.43–202.14	191.94–194.38	169.26	176.4	185.66	189.67
SCARABAEIDAE		199.64	176.96–181.84	152–158	116.85	156.06	170.36	174.78
*phytophagous clade* Pleurosticti	*Plant material*	177.38	152–158	142.31–146.91	101.4	138.11	151.25	155.56
Sericini	Leaves	153.56	133.75–138.29	125.31–129.32	88.39	119.19	130.37	134.54
Melolonthinae 1	Leaves	150.81	131.75–136.06	123.8–127.27	86.22	116.84	128.23	132.02
Melolonthinae 2	Leaves	160.73	140.33–145.06	131.95–135.7	90.88	125.2	137.16	141.12
Hopliini	Leaves & pollen	150.24	131.92–136.96	124.18–127.54	84.43	117.05	128.25	131.96
*“CRD clade”*	*Angiosperm tissue*	155.7	136.27–140.74	128.23–131.79	84.32	121.36	132.93	136.76
Cetoniinae (including Valgini)	Sap, fruit, nectar & pollen	142.35	124.86–128.84	117.57–120.75	73.57	110.8	121.42	124.95
Rutelinae1- Anoplognathini	Flowers, floral parts & leaves	115.02	101.35–104.46	95.59–98.09	46.26	89.99	98.53	101.33
Rutelinae 2b- Anomalini	Flowers, floral parts & leaves	110.36	97.28–100.18	91.74–94.08	37.2	85.76	94.04	96.8
Rutelinae 3-Adoretini	Flowers, floral parts & leaves	74.6	65.18–67.75	62.09–63.65	16	57.95	63.56	65.42
Dynastinae	Tubers, corns, fruit & flowers	117.92	104.22–107.28	98.4–100.87	48.6	92.19	100.89	103.78
*Saprophagous clade*	*Detritus*	173.17	155.09–158.84	139.37–143.81	91.94	130.54	147.25	151.52
Aphodiinae	Detritus & dung	160.04	143.54–146.95	129.92–133.85	88.12	121.01	136.11	140.09
Scarabaeinae	Detritus & dung	143.3	128.6–131.58	118.76–121.85	83.5	**92**	121.26	124.99
Scarabaeinae b	Dung	110.77	99.54–101.83	93.18–95.3	66.31	80.54	93.58	96.54

Predicted ages of selected scarab lineages using two different fossil sets for calibration analyzed using penalized likelihood methods. See also Table 4 for additional node ages and Bayesian estimates of selected schemes and Fig 2 for differences in accumulation of ingroup taxa for all analyses. Generalized feeding biology is also listed. Exceptions to these generalized adult diets exist but only represent a small proportion of diversity and derived feeding ecologies

### Cross-validation of estimated ages and fossil record

Table 5 summarises the congruence between the estimated ages of clades from our analyses and fossils used as calibration points. Cross-validation analyses highlight that divergence estimates are strongly influenced by how fossil calibration points are treated. Mean age estimates from CSi (maximum bounds applied to 9 ingroup taxa) suggest that the Scarabaeidae diverged ~116.85Ma (PL) or 122.5Ma (Ba), inconsistent with scarab fossils from the Melolonthinae (>130Ma), Aegialiinae (>130Ma), and “Aclopinae” (152-158Ma) [33–34]. Only when 8 Cenozoic, ingroup calibration points were removed from analyses CSii-iv, the estimated age of the Scarabaeidae became congruent with the fossil record (156.06 (CSii PL), 170.36 (CSiii PL), 173.35 (CSiii Ba) or 174.78Ma (CSiv PL)).

### TESS analyses

No significant changes in speciation or extinction rates were detected in any of the TESS/CoMET analyses conducted, including investigation of scarabaeine dung beetles or phytophagous Pleurosticti clade (see S4 Fig). Within the Scarabaeinae, a potential mass extinction event in the Upper Cretaceous ~85-95Ma was detected in PL analyses of CS2, CS3 and CSiii but never in the MCMCTree analyses. We believe these differences are driven by topologies as the PL tree contained polytomies.

## Discussion

### Systematics and Diversification

#### Divergence time estimates: Scarabaeoidea

The superfamily Scarabaeoidea was recovered as a strongly supported monophyletic lineage consistent with other studies [8–9, 18, 20]. Given that *Allioscarabaeus cheni* represents an extinct family-level lineage within the superfamily, it was not used as a calibration point however is useful to cross-validate the age estimates from our analyses. We tested 7 calibration schemes, five of which (CS2-3, CSii-CSiv) predicted the mean age of origin to be in the Lower Jurassic (~176-203Ma), consistent with the age of *Allioscarabaeus cheni* [13] and inline with the estimates of Hunt et al. [8] (~191.4Ma) and Ahrens et al. [18] (~174.3–190.9). Analysis CS1 was only calibrated with the estimated molecular age of the Polyphaga and predicted an Upper Triassic origin (~216Ma), while the most conservative analysis CSi with maximum bounds on Paleogene and Cretaceous fossils, predicted an Upper to Middle Jurassic origin (~160-170Ma). To date only the analysis of McKenna et al. [9] has predicted a Lower Cretaceous origin of Scarabaeoidea of 141.11Ma (161.0–116.87Ma), however this is in conflict with the Jurassic fossil record, which documents that at least three extant families (i.e. Lucanidae from Shara-Teg formation, Anda-Zhuduk and Daohugou Village Mongolia [53–55], Hyborsoridae from Karatau, Kazakhstan [56] and Alloioscarabaeidae from Jiulongshan formation, Mongolia [13]).

Given that calibration schemes CSi-iv use these lucanid and hyborsorid fossils as constraints we cannot cross-validate these node ages, however CS2-3 did not use these calibration points. The crown age of the Hyborsoridae in CS2-3 is estimated to have originated in Middle to Lower Jurassic (~172-177Ma) while the subfamily Hyborsorinae originated in the Upper Jurassic (~145-149Ma) (see Table 5) congruent with the oldest recorded Hyborsorinae fossils (*Protohybosorus grandissimus* from the Jurassic (155.7–164.7 Ma) [56] and *Fortishybosorus ericeusicus* from the Lower Cretaceous, Yixian formation [57]). Evidence for early hyborsorid diversification may be taken from the record of *Mesoceratocanthus tuberculifrons* (Hyborsoridae: Ceratocanthinae) from the Lower Cretaceous, Yixian formation [58]. Our CS2-3 estimates predict the family Lucanidae originated in the Lower Jurassic (174-180Ma) and subfamilies began to diverge in the Middle to Lower Jurassic (~150–165 Ma) (see Table 5), these results are consistent with ages of known fossils of three lucanid subfamilies: *Paralucanus mesozoicus* (Paralucaninae) from Shara-Teg (145.5–150.8), *Protolucanus jurassicus* (Protolucaninae) from Anda-Zhuduk (145.5–150.8Ma) and *Juraesalus atavus* (Aesalinae) from Daohugou Village (159.8 Ma)[54–55]. Our results are also congruent with the estimates from a molecular phylogeny of the Lucanidae [19] which estimated the origin of the crown group Lucanidae *sensu strictu* at ~160Ma (154-171Ma) and the crown group Lucanidae *sensu lato* (includes Lucanidae *s*. *s*. Aesalinae, Diphyllostomatidae and the extinct lucanid subfamilies) at 167Ma (155-182Ma). Given that the fossil age of *Protohybosorus grandissimus*, *Paralucanus mesozoicus*, *Protolucanus jurassicus* and *Juraesalus atavus* are largely congruent with our CS2-CS3 estimates, this cross-validation highlights that our results do not appear to be grossly inflated. It also suggests the age of the Scarabaeoidea is in the Lower Jurassic as suggested by Hunt et al. [8], Ahrens et al. [18], and here in analyses CS2-3, and CSii-iv.

#### Divergence time estimates: Scarabaeidae

Ahrens et al. [18] did not recover a monophyletic Scarabaeidae so the timing of the origin of the family cannot be compared, however with the exception of CSi which was designed to replicate their analyses, their results consistently estimated much younger ages for major lineages within the Scarabaeidae than in the present study. The major differences between analyses lie in fossil selection and calibration method, i.e. selecting Cenozoic fossil and using exponential priors with “soft” maximum bounds. Exponential priors are best used when “there is a strong expectation that the oldest fossil lies very close to the divergence event being represented by the node, relative to a distant, soft maximum” [59]. Given that the majority of fossils used in Ahrens et al. [18] were relatively young (Cenozoic) and were placed at nodes representing the most conservative interpretation of the taxonomic hierarchy (i.e. subfamily or tribe), which may have significantly underestimated the divergence dates when constrained using exponential priors with soft maximum bounds. To examine the effect of exponential priors with soft maximum bounds, we can compare analyses constrained with (CSi) and without (CSiii) maximum bounds on the ingroup taxa (Tables 3 and 6). The mean age of Scarabaeidae is estimated to have originated in the Aptian (113-125Ma) when maximum bounds are applied (CSi), or in the Aalenian (170.3–174.1Ma) when maximum bounds are excluded (CSiii), even considering the 95% confidence intervals of the Bayesian analyses there is no overlap between estimates (CSi: 99.2–156.7Ma; CSiii: 162.6–185.7Ma). This pattern is consistently recovered across ingroup clades, with lineage ages always estimated as significantly younger if maximum bounds are applied (CSi) than when only a minimum bound is applied (CSiii). Similarly the effect of removing calibrations with maximum bounds can be observed within Ahrens et al. [18], which excluded calibration points in some runs if they were identified as being in conflict with other settings in their BEAST analyses. For example the Aphodiinae calibration point “F” in runs 2–6 and the *Aphodius* calibration point “H” in run 4 were excluded, as a result the estimated age of Aphodiinae differs between run1 = 65.5Ma (57.3–73.3) and run2 = 69.2Ma (58.4–79.9) (includes calibration points F and H), and run3 (F is excluded) = 111.8Ma (94.8–129.7) or run4 (F and H are excluded) = 124.7Ma (108.0–142.7)[18].

Of the 10 ingroup fossils selected by Ahrens et al. [18] used to calibrate CSi, only one is Cretaceous, while nine are Cenozoic (1 Paleocene, 7 Eocene and 1 Miocene). Although these fossils are unequivocally assigned to the corresponding taxa, uncertainty exists as to whether they all represent the earliest members of their respective lineages, thus raising concerns regarding calibration using maximum bounds. Ahrens et al. [18] do not address the mismatch between their age estimates and other known reliable scarab fossils reviewed by Krell [33]. When other known scarab fossils are considered, mismatches between node estimates inferred by Ahrens et al. [18] and in Csi are observed. For example, Ahrens et al. [18] recover an overall mean age of the tribe Sericini as ~93.4–111.3Ma (95% confidence intervals 81.3–124.7Ma) depending on various constraints including stem and crown placement of a Nearctic Sericini fossil from the Oligocene. However, the tribe Sericini is represented by multiple species from two genera in the Lower Cretaceous in the Zazunskaya and Leskovskaya Formations of Russia (~140-145Ma) and next appear 100my later in the fossil record, at Florissant, USA (33.9–37.2Ma) [33]. Our estimate in CSi, also calibrated with this Oligocene fossil, recovers an even younger origin of the Sericini (88.39Ma (PL) or 74.42Ma(63.4–84.0)(Bayesian)) inconsistent with the Cretaceous fossil record. Similarly, the age estimates for Melolonthinae recovered in CSi or Ahrens et al. [18] are not consistent with the fossil record. Given that Melolonthinae is recovered as a paraphyletic lineage within a monophyletic Pleurosticti clade, the crown age for the Pleurosticti can be used as a guide for the maximum age of stem group melolonthines. Ahrens et al. [18] estimate the crown age of the Pleurosticti clade in the Lower Cretaceous (mean age 108.9–128.1Ma; 95% confidence intervals 95.9–142.1Ma) while our CSi estimates are again younger (101.4Ma (PL) or 90.86Ma (83.6–99.4Ma) (Bayesian)). Only when the maximum estimate from the 95% confidence intervals are considered, is one of the six runs of Ahrens et al. [18] congruent with the age of *Cretomelolontha transbaikalica* from the Baissa formation Russia (140.2–145.5Ma)[60]. As is the case for tribe Sericini, a gap in the fossil record for the subfamily Melolonthinae exists, the next oldest melolonthine fossils are known from the Eocene (33.9–56.0Ma) [33]. These extreme gaps in the fossil record highlight that the fossil record is poor for the Scarabaeidae and it is unlikely that the earliest branching members of major lineages have all been discovered. It is interesting to note that only trace fossils and no body fossils of scarabs are known from the Upper Cretaceous (66.0–89.8Ma), despite being represented in the middle Cretaceous by Aphodiinae (*Cretaegialia* spp.) and Scarabaeinae (*Prionocephale deplanate*) and in the Lower Cretaceous by Melolonthinae including, but not limited to, Sericini [33]. Paleocene scarabaeoid fossils are also rare and represented only by Aphodiinae (*Aphodius charauxi*) and possibly Rutelinae (i.e. *Anomalites fugitivus* is reported from the Tertiary without specification [61]). Given the rarity of scarabaeoid fossils, including the absence of body fossils from the Upper Cretaceous, we believe that applying maximum bounds on fossils (particularly those from the Cenozoic) are inappropriate and we therefore consider the results of Ahrens et al. [18] and CSi to be underestimated.

When we consider all the other analyses inferred without maximum bounds applied to Cenozoic fossils (CS2, CS3, CSii, CSiii and CSiv) the age of the Scarabaeidae is consistently estimated to be in the Jurassic (see Table 6). Of these five analyses, only CSii has a maximum bound applied at a subfamily level (at the Scarabaeinae node: 83.5-92Ma); in all other analyses this node is only constrained by the minimum age bound. Consequently, the predicted ages of ingroup nodes are also always younger in CSii than for analyses without the maximum bound on the Scarabaeinae. Given the reported mismatch in estimated ages and the earliest known fossil, we consider the results of CSii are also likely to be an underestimate driven by an inappropriate analysis constraint. For the purpose of examining potential diversification hypotheses we consider the results of CS2, CS3, CSiii and CSiv to be plausible on the basis of cross-validation of molecular estimates and the known fossil record, and because estimates are not driven by maximum bounds applied to ingroup taxa. Although the age of Scarabaeidae is constrained in CS3 (152-158Ma) the results are congruent with the results from other analyses and so we consider them plausible.

We tested two different fossil sets to calibrate the analyses, one based on the literature reviewed by Krell [33] and the second deemed to be unequivocally assignable by Ahrens et al. [18]. Key morphological characters used to identify scarabaeoids are often poorly preserved in fossils so their exact systematic placement remains unclear, with various researchers taking more or less conservative views on their classification. One such example is the fossil of *Juraclopus rohdendorfi* Nikolajev, which was originally assigned to the tribe Aclopini (Scarabaeidae: Aclopinae) [62]. Krell [33–34] accepts this placement and as such considers this the oldest known fossil from the family Scarabaeidae. Although Ahrens et al. [18] consider many of the fossils reviewed by Krell [33–34] to be unequivocally assignable, they provide no reasoning as to why *Juraclopus* is excluded from their fossil constraints. This fossil may have been excluded on the basis of uncertain placement within the phylogeny (Scarabaeidae is not monophyletic and no aclopines were included in their taxon sampling) or doubts over the identification of the fossil. Our analyses recover a monophyletic Scarabaeidae and include an aclopine that is recovered in the Pleurosticti clade, as such we test the placement of *Juraclopus rohdendorfi* at both the crown (CS2) and the stem (CS3) of the Pleurosticti clade. Interestingly, the estimated age for the origin of Scarabaeidae when not constrained with *Juraclopus rohdendorfi* (CSiii-CSiv) is highly congruent with the estimate obtained from CS2 suggesting that the fossil represents a member of the crown group Pleurosticti and also highlights that analyses are coming to a consensus for divergence estimates that predict the origin of the Scarabaeidae in the Aalenian-Toarcian (170.3–182.7Ma).

Another such fossil with debated identification is *Prionocephale deplanate* Lin, while Krell [33–34] and Ahrens et al. [18] list the fossil as assignable to Scarabaeinae, Tarasov & Génier [63] are less convinced. Tarasov & Génier [63] comment that the original description does not list any unambiguous characters of the subfamily, however, no specific explanation for or against the original identification is provided. It is clear further review of such Mesozoic scarabaeoid fossils is warranted; as such it is important to examine evolutionary hypotheses beyond the known and limited fossil record. Here we compare the timing of well-documented ecological events such as angiosperm and mammal evolution, continental drift and the Cretaceous-Paleogene mass extinction to explore our hypotheses on scarab beetle diversification.

#### Scarab diversification

The Scarabaeidae is divided into two strongly supported lineages representing major ecological groupings of phytophagous and saprophagous scarabs. Within the phytophagous lineage (the Pleurosticti), Melolonthinae is recovered as a paraphyletic grade with subclades representing major tribes, while Rutelinae, Cetoniinae and Dynastinae stem from a common ancestor (the CRD-clade) that diversified into the currently recognized subfamilies and tribes. Within the saprophagous clade, Aphodiinae form the most-early branching lineages and a monophyletic Scarabaeinae is supported. These results suggest both specialist phytophages and saprophages evolved from generalist ancestors, providing a compelling framework to examine feeding biology and the response to major biotic and abiotic evolutionary events. The divergence dates estimated in CS2-3 and CSiii-iv infer that the crown group Pleurosticti originated in the Upper Cretaceous. While the crown group of the saprophagous clade originated around the Jurassic-Cretaceous boundary, specialist adaptations to both of these feeding biologies originating in the middle of the Cretaceous. These age estimates significantly predate the mid-Cretaceous crown group origins of both clades predicted by Ahrens et al. [18], which as discussed above, may be an artifact of inappropriate calibration. Ahrens et al. [18] proposed that the evolution of scarab beetles tracks the sequential rise of angiosperms and mammals, however our results suggest an origin of the phytophagous lineage predating the ecological dominance of angiosperms and that specialist saprophages vastly predate mammal dominance.

### Influence of Angiosperms on diversification

**Fig 3:**
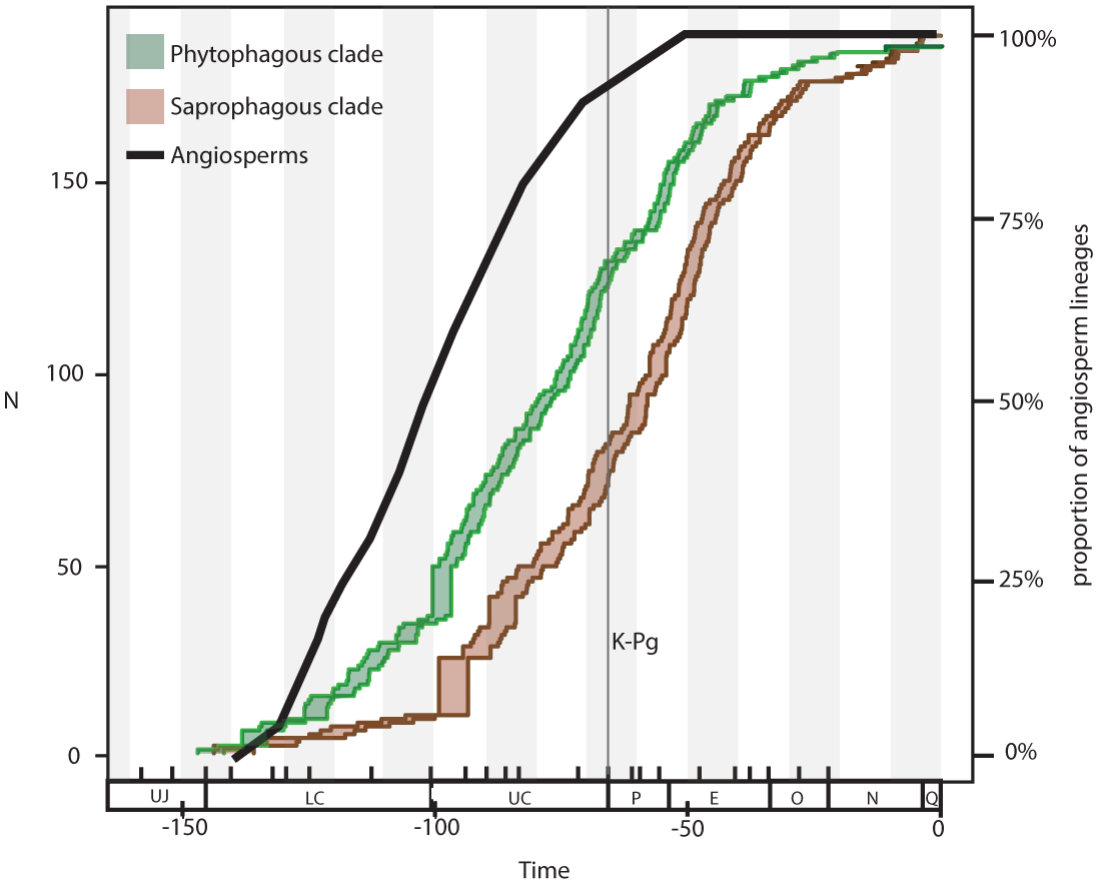
Lineage through time (LTT) plots of scarab lineages. LTTs are compared to the proportion of extant crown group angiosperm lineages as per Schneider et al. [115]. Shaded areas represent congruent estimates from analyses CS2, CS3, CSiii and CSiv estimated by Penalized Likelihood methods with divergence maxima plotted from CS2 and minima from CSiii.

**Fig 4:**
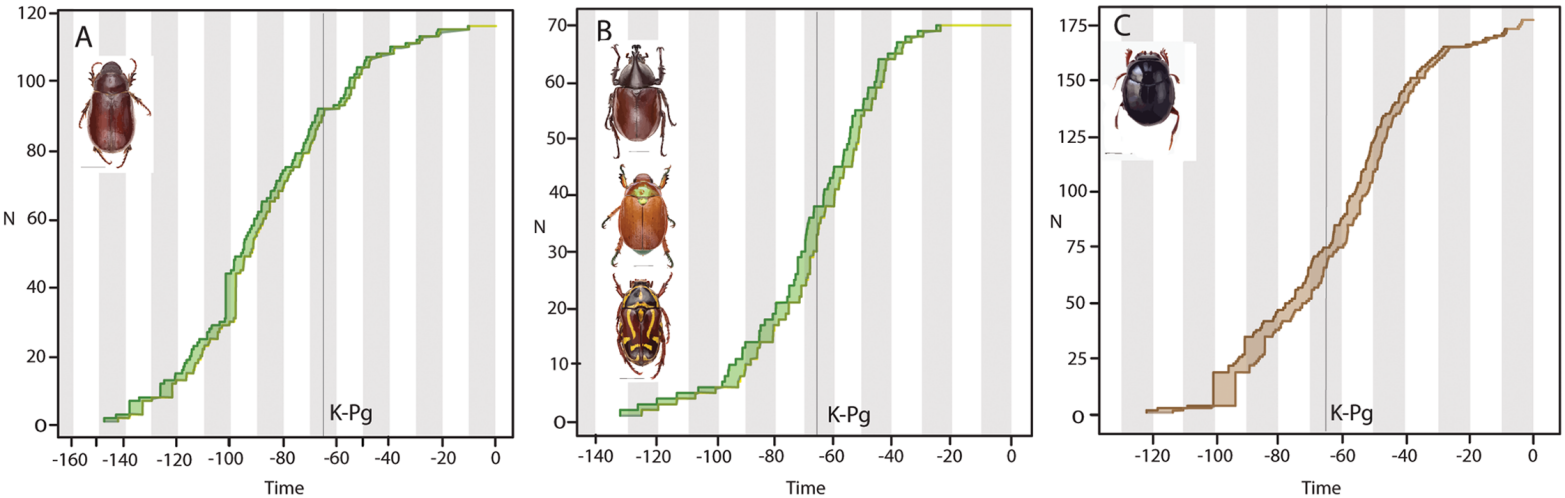
Lineage through time plots of scarab clades. A) Melolonthinae (leaf feeders) B) CRD clade (specialist angiosperm feeders) C) Scarabaeinae (dung feeders). Shaded areas represent congruent estimates from analyses CS2, CS3, CSiii and CSiv estimated by Penalized Likelihood methods with divergence maxima plotted from CS2 and minima from CSiii.

While estimates for the age of angiosperms vary, placing origins in the Jurassic or Triassic: 141-199Myr [64], 182-270Myr [65], 225-240Myr [66], or 221-275Myr [67], agreement exists that by the Albian-Turonian (90–110 Ma), angiosperms dominated the environment [68]. Our estimates (CS2-3, CSiii-iv) predict the origin of the crown group Scarabaeidae in Middle to Lower Jurassic, with initial diversification of phytophagous scarabs at the Jurassic/Cretaceous boundary (see Fig 3). The oldest melolonthine lineages diverged ~130–150 Ma, suggesting that their initial evolution was either with the very earliest angiosperms or non-angiosperms as food plants. Their peak radiation was 95–115 Ma (Fig 4A), lagging the origin of crown group angiosperms as has been reported in other leaf feeding beetles [5, 69], including scarabs [18]. In contrast, the CRD-clade (Rutelinae+ Cetoniinae + Dynastinae) that feed on angiosperm-specific plant tissues originated 128-140Myr and split into the recognized subfamilies 92-127Myr. The CRD-clade diversified at an accelerated rate from 90-105Myr (Fig 4B), then accumulated at a similar rate to the leaf feeders until 70-80Myr, after which point they accumulated more rapidly than melolonthines. Ahrens et al. [18] estimate the mean age crown group origin of the Pleurosticti ~110-130Myr, with the specialist CRD clade originating ~90Ma (interpreted from Figure 1 [18] as not explicitly listed) and diverging into the recognized subfamilies ~46-72Ma. Although both our analyses and those of Ahrens et al. [18] suggest a co-radiation with angiosperms, our results predict a much smaller time lag, over 20Myr earlier than previously hypothesised with the timing of the first inferred peak of diversification of phytophagous scarabs corresponding with the ecological dominance of angiosperms ~90–110 Ma [68] (see Fig 3).

Interestingly, saprophagous scarabs underwent a similar diversification pattern to that observed in the phytophagous subfamilies, originating prior to or with the earliest angiosperms, and underwent a significant radiation 90-110Ma, corresponding to the major expansion of angiosperm diversity (Fig 3). This pattern is largely driven by the dung-specialist Scarabaeinae due to our limited aphodiine sampling. Ecologically, the main terrestrial vertebrate fauna of the Cretaceous were dinosaurs. Given that angiosperms only represented a small proportion of the floral diversity prior to the Albian (>113Ma), it is likely that angiosperms formed only a minor component of Lower Cretaceous dinosaur diet [70]. The oldest direct evidence of angiosperm feeding is from the fossilized gut content of an Albian ankylosaur containing various angiosperm fruit [71], however coprolites and enterolites are rare [70]. The incorporation of angiosperm tissue in to the diet of dinosaurs is supported by evidence from teeth and postulated jaw function of herbivorous dinosaurs, however it was likely that gymnosperms remained a significant component of their diet until their extinction [70,72]. While faeces of herbivorous dinosaurs were certainly available before angiosperms originated, it may have been unsuitable for scarab feeding. Studies on scarabaeine dung beetle feeding show particle size is a critical determinant of dung selectivity [73]. The incorporation of less fibrous angiosperms into the diet of herbivorous dinosaurs with grinding gastroliths [74] could have produced sufficiently fragmented dung suitable for scarab feeding. Non-angiosperm plants such as gymnosperms, ferns and allies are also of lower nutritional quality [75] so changes in floral composition were likely reflected in the available dung. We hypothesize that even if angiosperms were only a minor component of dinosaur diets, the less fibrous, more nutritious dung provided a new niche in which dung beetles first evolved. It also provides a model whereby the radiation of a non-phytophagous beetle group is driven by rise of angiosperms despite their ecological link being at one remove, i.e. through a dinosaur’s digestive system.

### Origin of dung feeding

The co-evolution of scarabaeine dung beetles with dinosaurs is controversial. Trace fossils within herbivorous dinosaur coprolites attributed to tunneling dung beetles appear in the Upper Cretaceous, the oldest from the Campanian [72, 76], younger than the oldest Scarabaeinae fossil (*Prionocephale deplanate* Lin)[33]. Conversely, it has been argued that the mixing of uric acid with feces common to all non-mammals would have made dinosaur dung unsuitable [77], a hypothesis supported by the rarity of extant dung beetles utilizing reptile or avian dung. Molecular-clock based estimates for the crown age of marsupials and placental mammals are as old as 82 and 101Ma, with peak mammal diversification between 75-85Ma [78]. Fossil evidence however is inconsistent with the molecular-clocks, suggesting a mammal crown group age of 65Ma and peak diversification over the next 10Ma [79]. Even if fossil evidence underestimates the age of mammals, they were not a significant component of the Cretaceous fauna and our evidence shows that both the origin and crown group radiation of most dung beetle tribes (~119-130Ma and 85-98Ma respectively) predates the major mammal radiation. Furthermore, it is unlikely that coprophagy by scarabaeines evolved in association with the small insectivorous stem-group mammals present in the Mesozoic [78] as very few extant scarabaeines feed on small dry dung pellets with only specialists using insectivore dung as a resource [73].

A strong mismatch between the inferred divergence times of both mammals and dung beetles provides the first molecular evidence that the initial evolution of coprophagy in scarabaeines was associated with dinosaurs and not mammals. Ahrens et al. [18] estimated the mean age of dung feeding Scarabaeinae to be ~72-85Ma, thus attributing their origin to co-evolution with mammals alone. However our reanalysis suggests that inappropriate fossil calibrations with maximum bounds are responsible for these younger ages. Historical biogeographical scenarios also support our significantly older age of scarabaeine dung beetles than the past molecular estimates. Two of the oldest dung feeding tribes, Dichotomini and Canthonini, have strong Gondwanan distributions [63, 80–82], resulting from either vicariance or dispersal. Our results suggest that with the origin of dung feeding Scarabaeinae is congruent with the continental breakup of Gondwanaland at ~95-100Ma with the Gondwanan tribes diverging ~85-95Ma (Table 3). The Gondwanan distribution of Dichotomini and Canthonini is improbable with the origin of dung feeding Scarabaeinae at ~76Ma.

### Biogeography of Scarabaeinae

The geographic origin of dung beetles remains controversial with many reviews of biogeographic evidence suggesting a Mesozoic origin of scarabaeines from Gondwanan ancestors [80, 82–83]. Alternatively, Sole & Scholtz [84] proposed an African origin of dung beetles based on a 3-gene phylogeny of 25 African genera from the tribes Dichotomini and Canthonini. Using molecular clock methods, the age of the origin of the two tribes was estimated at around 56Ma. Sole & Scholtz [84] concluded that given this inferred age and as representatives of these early branching scarabaeines are found in Africa, the fauna therefore originated and radiated on Africa and that dispersal is responsible for their current distribution. A similar study of African Scarabaeinae that sampled 4 genes from 55 of 105 African genera suggested the earliest split in the subfamily in Africa occurred between 42 and 27 Ma based on molecular clock estimates of COI data only [85] and tested two differing substitution rates used in previous analyses of scarabaeine divergence [84,86]. Mlambo et al. [85] question the validity of their older estimates due to poor resolution of a number of parameters, and favour the higher arthropod substitution rate of 0.012 mutations per million years to suggest an origin of the subfamily at 33.9Ma (as listed in Fig 6 of [85]). To date the most extensively sampled molecular phylogeny is that of Monaghan et al. [87] which sampled 3 genes from 214 species representing all continents (excluding Antarctica) to explore systematic relationships of the Scarabaeinae, evolution of nesting strategies, and biogeographic scenarios. The Dispersal-Vicariance (DIVA) analysis supported an out of Africa hypothesis, with long distance dispersal to all other continents with little back migration [87], however no timeline was given for the origin or dispersal of scarabaeine lineages. Phillips et al. [81] suggested a Mesozoic/Cretaceous origin of scarabaeine dung beetles with subsequent diversification in the Tertiary, and hypothesized that a combination of vicariance and dispersal (predominately from Africa) accounted for the present distribution of dung beetle tribes. While long distance dispersal is certainly likely for the distribution of some tribes (e.g. Onthophagini, Oniticellini and Sisyphini), long distance dispersal is unlikely for clades containing both Old World and New World taxa due to their limited flight abilities [63]. The biogeographic distribution of scarabaeine tribes was discussed in detail by Tarasov & Génier [63], under two evolutionary scenarios, Cenozoic vs. Mesozoic origins, who suggested that the distribution of dung beetles better fit a vicariance hypothesis. Tarasov & Génier [63] hypothesized that if the origin of the Scarabaeinae coincided with the separation of Gondwana from Africa (~160Ma) and clades containing both Old World and New World taxa originated while Africa and South America remained connected (~110-95Ma) then the phylogenetic pattern recovered within their morphological phylogeny would support a vicariant pattern.

Previous studies of African dung beetles [84–86] rely on molecular clock methods due to the limited fossil record. The use of molecular clocks remains controversial as ‘standard’ substitution rates are often calculated from small samples of closely related species and given the influence of calibration choices on calculated rates [88] ‘standard’ substitution rates may not be realistic due to rate variation rates across time scales [89] or among invertebrate species [90]. For example, Wirta et al [86] and Mlambo et al. [85] both use a standard range of rates of 0.0075 and 0.012 substitutions/site/Ma, yet the results from these two studies are incongruent with each other. Wirta et al. [86] hypothesises that Malagasy Oniticellini/‘Helictopleurini’ diverged from the African Oniticellini either 44(29/64) or 28(18–39) Ma for the rates 0.0075 and 0.012 substitutions/site/Ma, but Mlambo et al. [85] in replicating Wirta et al.’s [86] methods, estimated the earliest split in the Scarabaeinae at 42(32/53) or 27(20/35) Ma and origin of Africa Oniticellini, a derived tribe, at ~13.2Ma. Such inconsistencies highlight the limitations of molecular clock methods. To date, only the divergence dating analyses of Ahrens et al. [18] and those presented here are calibrated using fossil data and samples from beyond the Scarabaeinae and Aphodiinae to allow the incorporation of more distant calibration points. The origin of Scarabaeinae inferred by these later studies [present study, 18], significantly predate the molecular clock based estimates [84–85], which may be attributed to fossil calibration, or the broader taxonomic and geographic sampling. Our various calibration schemes recovered the origin of scarabaeine dung beetles in the Lower Cretaceous (mean age Aptian-Berriasian, minimum to maximum 95% confidence 116.5–152.4Ma), roughly consistent with the split of Africa from Gondwana. The opening of the South Atlantic Ocean and the split from Africa and Gondwana is poorly understood, however the general consensus based evidence from plate tectonics is that the intercontinental rift was initiated ~140Ma and that complete separation of the two continents occurred just prior to the Aptian, ~126Ma [91–93]. This timing is consistent with hypotheses derived on the similarities in Vertebrate fauna, that South America, Africa, Madagascar, India and Australia remained partially connected until the Aptian [94–98]. It has also been proposed that faunal exchange between Africa and South America may have persisted until the Albian-Cenomanian through a connecting land bridge [99], a chain of islands or through Antarctica and Australia [96–97]. Given the inferred age of Scarabaeinae in the Lower Cretaceous, the major radiation of dung feeders prior to the Cenomanian, and early divergence of both African and Gondwanan lineages, we hypothesis that the faunal exchange between Africa and Gondwanaland occurred during the earliest evolution of the Scarabaeinae. Therefore we propose that both Gondwanan vicariance and dispersal of African lineages is responsible for present day distribution of the Scarabaeinae.

Support for a vicariance pattern can be observed in the Canthonini clade 2 representing Australian, Malagasy and New Caledonian fauna, estimated to have originated ~85-105Ma with the new Caledonian genera diverging ~50-55Ma and splitting from a clade containing the Australian genera *Demarziella* and *Diorygopyx* ~ 76-82Ma (Table 3). These ages are broadly consistent with the break-up of the Gondwana landmass with New Caledonia (+ New Zealand) splitting from Gondwana ~80Ma. Unfortunately no New Zealand dung beetles were included in our analysis to test the subsequent splitting of New Caledonia and New Zealand ~30–40 Ma. Further investigation is needed to confirm our hypothesis that the origin of Scarabaeinae occurred while Africa and South America were still connected but our results indicate that biogeographic distribution and the estimated origin of Canthonini fits a vicariance scenario.

In terms of endemic scarabaeine radiations, the origin of the Malagasy Oniticellini (often classified as Helictopleurini) is interesting to consider in a biogeographic context. The lineage was paraphyletic in our original Bayesian analysis but the 5 *Helictopleuris* spp. were recovered as monophyletic in the fully-bifurcating tree used in MCMCTree analyses. The mean age of crown group “Helictopleurini” in MCMCTree analyses was estimated at 61.16–66.17Ma while PL estimated a minimum age of 56.18–60.92Ma in PL for the clade containing 3 of 5 *Helictopleuris* species sampled. Given that Madagascar split from India ~80Ma [100], long distance dispersal best explains the biogeographic pattern of Malagasy Oniticellini. This dispersal hypothesis is in agreement with the findings of a comprehensive molecular phylogeny of “Helictopleurini” that dated the origin of the lineage ~23–37 Ma using molecular clock methods [86]. Wirta et al. [86] explored a scenario that radiation of “Helictopleurini” was triggered by the arrival and radiation of lemurs and other Malagasy mammals, stating that their estimates broadly corresponded to this hypothesis. Interestingly our estimates are remarkably congruent with the arrival of lemurs in Madagascar ~55-65Ma [101–103]. While our sampling is too limited to examine Malagasy radiations in detail, we can compare mechanisms for successful long distance dispersal to Madagascar from Africa in the Paleocene. The detailed study of spatial and temporal arrival patterns of Madagascar’s vertebrate fauna, tectonic history and oceanic currents demonstrated that Early Cenozoic surface currents were periodically conducive to rafting from Africa [104]. Successful transoceanic dispersal by rafters was highest in the Paleogene, with success decreasing over time, reaching its lowest levels in the mid-Miocene [104]. Although we do not rule out flight as the method of colonization, if Oniticelli arrived in Madagascar in the Paleocene, then transoceanic dispersal via rafting was possible. As such, we propose an alternative method of long distance dispersal to Madagascar given the limited flight ability of scarabaeine dung beetles and rarity of medium distance dispersal events [63], although rafting is less probable with an Oligocene origin as suggested by Wirta et al. [86].

### The impact of the K-Pg event and radiation in the Paleogene

The mass-extinction of non-avian dinosaurs in the Cretaceous-Paleogene (K-Pg) event, 65-66Ma would likely have had an impact on dung beetle diversity given the loss of an important resource, dinosaur dung. A rate shift is observed in the Scarabaeinae LTT plots around this period and provides evidence for co-extinction (Fig 4C). In contrast to the LTT analyses, TESS and CoMET analyses could neither identify a mass extinction, nor any significant rates of diversification or extinction both within the Scarabaeinae and phytophagous clades. This failure to identify an increase in diversification within the phytophagous lineage coinciding or with a sequential lag with the origin of angiosperms is surprising. Unfortunately TESS and CoMET methods are reliant on sampling fraction and with the diversity of these scarab lineages (~5000 Scarabaeinae and ~19800 Pleurosticti), our sampling fractions were always between 2–8%, raising questions as to whether these methods are suitable for such limited taxon sampling. Accordingly, the following discussion will focus on the LTT results as they are more robust to the sampling used in this study.

The composition of extant scarabaeine dung beetle guilds provides insight into their survival through the K-Pg extinction. Most extant dung beetles display clear preferences for dung based on host diet, digestive processes and dropping size [73], although a few extant species are extreme generalists exist which may feed on herbivore, carnivore and omnivore dung, bird and reptile droppings, insect frass, carcasses and dead insects (e.g. [105]). It is unknown if Cretaceous dung beetles were generalists or specialists, however if particle size is a critical determinant to dung feeding, it is unlikely Cretaceous scarabaeines were able to feed on both large droppings of mega-herbivore dinosaurs and small, dry pellets of the primitive insectivorous mammals. However many small (<100kg) herbivorous and omnivorous dinosaurs [74] existed during the Cretaceous and it is possible that dung from these animals could have been utilized by small, generalist dung beetles that could also feed on dung from primitive mammals and so survive the loss of dinosaur hosts.

The shape of our LTT plots (Fig 4C) is consistent with a partial extinction of Scarabaeinae around the K-Pg event [106], likely the loss of those species that were dependent on dung of large dinosaurs such as those recorded in coprolite trace fossils. The extant dung beetle fauna is likely to be descended from species either already adapted to Cretaceous mammal dung or generalists capable of utilizing the dung of both small dinosaurs and early mammals, or alternative resources. For ~20 Ma after the plateau in LTT plots, dung beetle diversity accumulates at its most rapid rate (Fig 4C). The radiations during the Paleogene could be attributed to the diversification of mammals, or indirectly to the rise of grasses ~70-60Ma [107]. The evolution of grasslands in the mid-Eocene and in-turn grazing mammals [79], may have provided a second important ecological opportunity for scarabaeine radiation, but at present, species-level sampling is not sufficient to disentangle the causes of the most recent radiations. However, the role that mammals play on species-level diversification and specialization of feeding in scarabaeines can be explored through richness and abundance of native mammals compared to dung beetle food resources. The islands of Mauritius, New Caledonia and New Zealand lack indigenous mammals (with the exception of native bats) and the species richness of their dung beetle fauna is also low (Mauritius n = 5 spp.; New Caledonia = 13 spp.; New Zealand n = 14 spp. [80]). These faunas are all hypothesized to have east Gondwanan origins, being closely related to Malagasy, or Australian and New Guinean faunas respectively [80], which in comparison exhibit more diverse mammalian and dung beetle faunas (Madagascar: 88 mammals excluding 29 bats [108], ~200 scarabaeines [80]; Australia + New Guinea: ~315 mammals excluding ~120 bats [109], ~430 scarabaeines [80]). A detailed study of food resources of the native dung beetles of New Zealand demonstrated that they have evolved a generalist diet of dung (native reptile, bat, bird and insect, and non-native mammals) and carrion [105]. Similarly, derived feeding biologies of Scarabaeinae have been connected to the abundance of large mammals in an ecosystem. Halffter & Matthews [12], noted that copro-necrophagous and necrophagous dung beetles were most common in the Neotropics, proposing a connection between the limited abundance of large mammals compared to open areas with large mammals (e.g. the Afrotropics) where there is almost a complete absence of necrophagous species. Necrophagy in Scarabaeinae is a derived trait, with the Neotropical tribes being either exclusively coprophagous, or predominately coprophagous and occasionally necrophagous [12]. Furthermore, almost all genera that have necrophagous species are predominately coprophagous or copro-necrophagous with the exception of *Deltochilum* which is fundamentally necrophagous but occasionally copro-necrophagous, coprophagous [12], and even predatory [110]. Unfortunately, species-level sampling is not sufficient to explore evolution of derived feeding ecologies in Scarabaeinae, however it is evident that the local and regional ecological factors potentially driving such specialization warrant further investigation.

**Fig 5:**
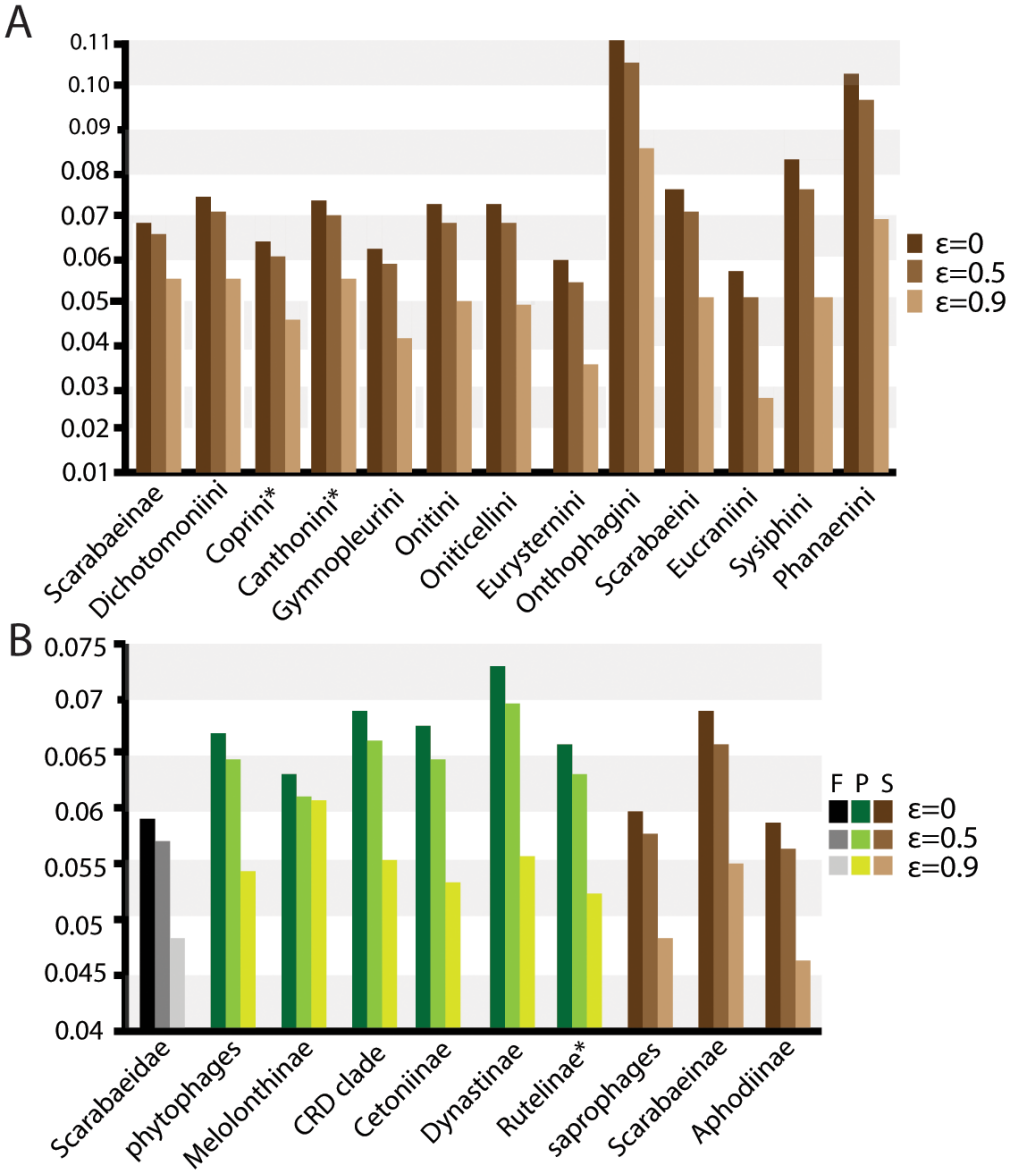
Scarab beetle diversification rates. Diversification rates calculated using the method of moments [48] and three extinction rates (ε = 0, 0.5, 0.9) of A) dung beetle tribes * The tribes Coprini and Canthonini were not recovered as monophyletic so the node age of Scarabaeinae B was used to estimate diversification rate; B) scarab clades and subfamilies. * The subfamily Rutelinae was not recovered as monophyletic so the node age of Rutelinae+ Dynastinae was used to estimate diversification rate. F = Family clade, P = Phytophagous clade, S = Saprophagous clade.

Comparison of diversification rates in scarabaeine dung beetle tribes that originate during the mid-Cretaceous versus end-Cretaceous/early-Paleogene provides further evidence for our hypothesis of co-extinction with dinosaurs (Fig 5). The Onthophagini represent ~40% of scarabaeine richness, yet originated approximately 20Ma after the oldest tribes Coprini, Dichotomiini and Canthonini (which represent 8%, 15% and 20% of richness respectively) (Table 7). The high diversification rate of Onthophagini (0.1102 lineages My^-1^) provides strong evidence of a rapid radiation in the Paleogene. Additionally, two of the three tribes that originated in the Paleogene display higher diversification rates than the average rate of the subfamily (Sysiphini = 0.083382, Phanaenini = 0.10314, vs. Scarabaeinae = 0.068635). Extant dung beetles from all tribes are intimately tied to feeding on mammalian dung and there is no reason to suggest that only these younger tribes underwent a rapid radiation with mammals in the Paleogene. Our LTT plots indicate that Scarabaeinae were accumulating quickly throughout the Upper Cretaceous, as such, the lower species richness and inferred diversification rates for the early-diverging tribes is best explained by extinction at the K-Pg boundary.

**Table 7:** Whole diversification rates estimated using the methods of moments [48].

	Estimated age	Number of species	r (0)	r (0.5)	r (0.9)
Scarabaeidae	180	27000	0.058939	0.057013	0.048193
Phytophagous clade	154	19800	0.066875	0.064625	0.054316
Saprophagous clade	157	8300	0.060060	0.057853	0.048674
Melolonthinae	154	11000	0.063059	0.061313	0.060808
“CRD clade”	13 8	8800	0.068753	0.066242	0.055800
Cetoniinae	126	3300	0.067516	0.064766	0.053332
Dynastinae	106	1500	0.07281	0.06955	0.05596
Rutelinae*	132	4000	0.06591	0.06328	0.05236
Aphodiinae*	145	3300	0.05867	0.05628	0.04634
Scarabaeinae	130	5000	0.068635	0.06590	0.054886
Dichotomini	94	750	0.074737	0.071055	0.05574
Coprini*	100	400	0.063965	0.060508	0.04613
Canthonini*	100	1000	0.07313	0.06973	0.0554303
Gymnopleurini	81	110	0.063019	0.05878	0.0411747
Onitini	78.5	200	0.0726499	0.068258	0.050002
Oniticellini	76	165	0.072506	0.067975	0.0491427
Eurysternini	74	53	0.05909	0.054502	0.03546
Onthophagini	73.5	2200	0.11022	0.10551	0.085914
Scarabaeini	71	150	0.076268	0.071421	0.051227
Eucranini	55.5	16	0.05709	0.051257	0.027437
Sysiphini	54	60	0.083382	0.075977	0.050812
Phaenini	52.5	150	0.10314	0.096589	0.069349

Clade age is the midpoint taken from molecular age estimates when calibrated using the minimum and maximum fossil age of *Juraclopus rohdendorfi* placed at the crown of the polyphagous scarab clade (E in Fig 1).

* represents taxon that were not recovered as a monophylum so the age of the node separating the oldest split was used to estimate diversification rates.

The impact of the K-Pg event on insect diversity is considered to be relatively minor with no family-level extinctions, rather species-level turnover whose intensity varied significantly between different regions [111]. Recently, K-Pg mass extinctions have been inferred for butterflies [112–113] and xylocopine bees [114] using similar methods to those used here. In each case the mass extinction of herbivorous insects is attributed to a loss of angiosperm diversity at the K-Pg. However, angiosperms only suffered a modest impact from the K-Pg event: negligible loss of family-level diversity globally and only moderate (18–30%) loss of families/genera at regional scales [50]. As such the cause of these mass extinctions of butterflies and bees is harder to directly infer given the minimal loss of angiosperm diversity. Within scarab beetles the significance of the K-Pg event may not be confined only to dung beetles (Scarabaeinae). The accumulation of Melolonthinae appears to halt from 55-65Ma before continued diversification at the same rate to those prior to the interruption (Fig 4A). Interestingly this pattern is not observed in the “CRD clade” of scarabs that are specialised to feed on angiosperm-specific tissues, thus highlighting that perhaps more complex interactions may be the cause of the loss of diversity of herbivores and pollinators at the K-Pg rather than direct loss of angiosperm diversity.

## Conclusions

In the absence of a clear and rich fossil record, divergence dating analyses of invertebrates will remain a complex task. We are no closer to reaching a consensus on the evolutionary history of scarab beetles in particularly scarabaeine dung beetles. Past studies have estimated the mean age of Scarabaeinae at 33.9Ma[86], 56Ma [85], 86.6–100.2Ma [18] and 118.8–131.6Ma [PL methods presented here]. The differences in estimates can be attributed to divergence dating methods (including calibration points, priors and programs), taxon or geographic sampling. We present the first evidence of the mid-Cretaceous origin, and Upper Cretaceous radiation of dung beetles, the timing of which is consistent with major global events including rise of the angiosperms and continental breakup of Gondwana.

The complex associations between plants and animals make it difficult to determine if single or multiple factors drove the evolution of contemporary biotas. This study of phytophagous and saprophagous scarabs revealed that angiosperms played a major role in the diversification of scarabs as both a direct and indirect food source. We hypothesise that a change in host diet, to incorporate more nutritious and less fibrous angiosperm foliage, provided a palatable dung source for dung beetle feeding thus creating a new niche for diversification. This indirect influence of angiosperms on dung beetle diversification opens up our understanding of how non-herbivorous insects (approx. 50% of species) evolved following the Cretaceous terrestrial revolution. The confirmation that dung-feeding scarabaeines evolved in the mid-cretaceous and rapidly diversified prior to the availability of a suitable mammalian dung source, provides the first inference of initial origin of any insect group to novel ecological interactions with dinosaurs and radiation due to specialization on a dinosaur resource, i.e. their dung. Our results reveal the complex evolutionary forces shaping these lineages, particularly scarabaeine dung beetles, which have undergone co-radiations with angiosperms and mammals plus potential co-extinction with dinosaurs. The reconstructed history of the Scarabaeidae highlights the complexity of evolutionary diversification and ecological adaptation and provides evidence for an extinction event not captured in the fossil record. As the loss of dung beetle diversity at the K-Pg can be readily associated with the extinction of dinosaurs, this is the first insect mass extinction for which we can confidently infer the cause.

## Supporting Information

S1 Fig The phylogeny of Scarabaeoidea. The phylogenetic tree is based on a partitioned 5 gene, 450- taxon Bayesian analysis. Posterior probability clade support values indicated at nodes >0.5.
(PDF)

S2 Fig Penalized Likelihood chronograms. Comparison of dated chronograms for seven calibration schemes analyzed in r8s.
(PDF)

S3 Fig Bayesian chronograms. Comparison of dated chronograms for four calibration schemes analyzed in MCMCTree.
(PDF)

S4 Fig TESS outputs. TESS outputs identifying rates and shifts in speciation or extinction, and significant mass-extinction events through time for (A) Pleurosticti (B) Scarabaeinae. Analyses CS2, CS3 and CSiii were examined using output trees from penalized likelihood and Bayesian divergence dating analyses. Posterior mean is represented by a solid bold line and 95% credible interval shadows. Solid vertical bars indicate posterior probability of a rate shift within the interval, with dashed line representing significance thresholds of 2lnBF = 2, 6, or 10 (BF = Bayes Factor). Time scale excludes most recent 45 or 50 Ma depending on analysis that corresponds to species level sampling (see S1 Table for parameters).
(PDF)

S1 Table TESS analysis settings. Penalized likelihood (PL) output from r8s, Bayesian output from MCMCTree. Sampling fraction (*rho*) is calculated from the number tips at the corresponding cut time and compared to the predicted diversity calculated using Method of Moments [48]. The most recent 45 or 50 Ma were excluded to minimize effect of limited species level sampling.
(DOCX)
