# Supplementary figures and images for: If Dung Beetles (Scarabaeidae: Scarabaeinae) Arose in Association with Dinosaurs, Did They Also Suffer a Mass Co-Extinction at the K-Pg Boundary?
Source: PLoS One. 2016 May 4;11(5):e0153570. doi: 10.1371/journal.pone.0153570 (PMC4856399; doi:10.1371/journal.pone.0153570)

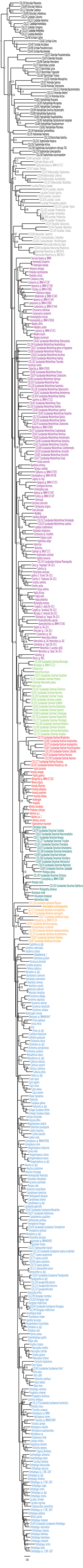

Supplement: S1 Fig — The phylogenetic tree is based on a partitioned 5 gene, 450- taxon Bayesian analysis. Posterior probability clade support values indicated at nodes >0.5. (PDF) [file pone.0153570.s001.pdf]

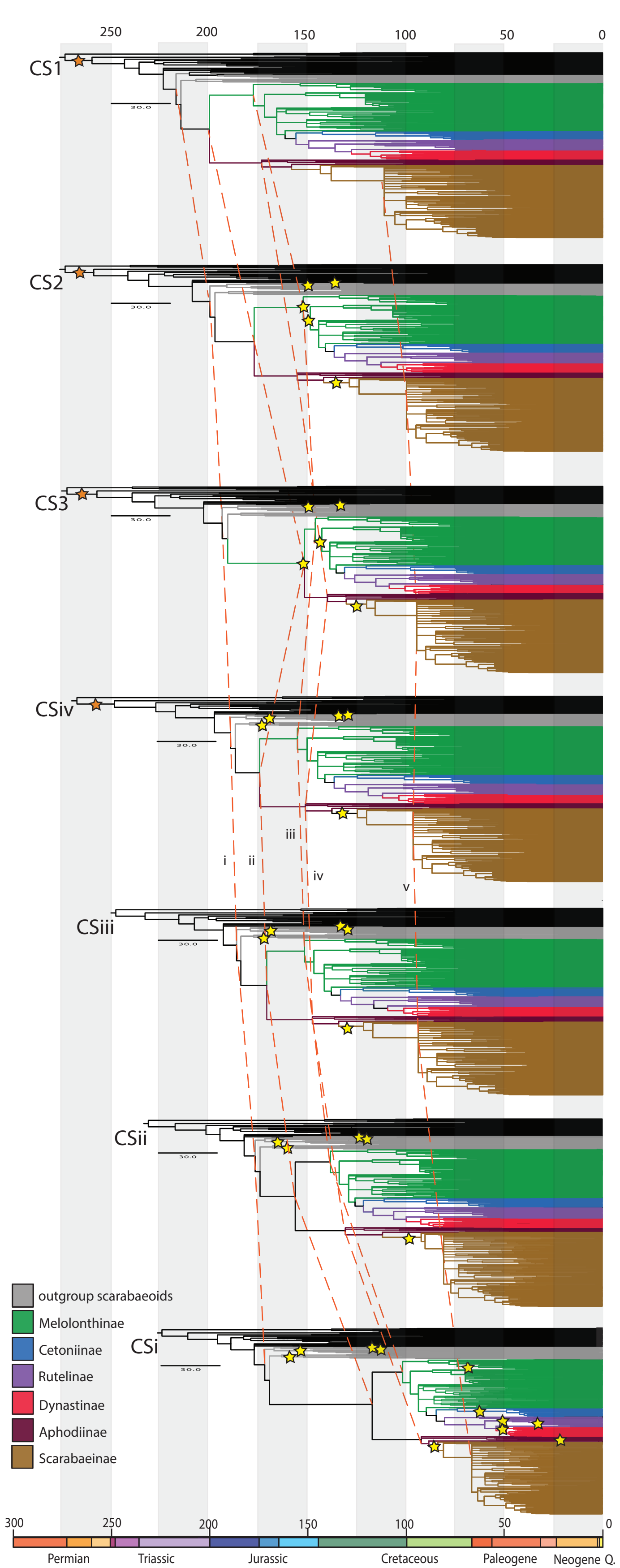

Supplement: S2 Fig — Comparison of dated chronograms for seven calibration schemes analyzed in r8s. (PDF) [file pone.0153570.s002.pdf]

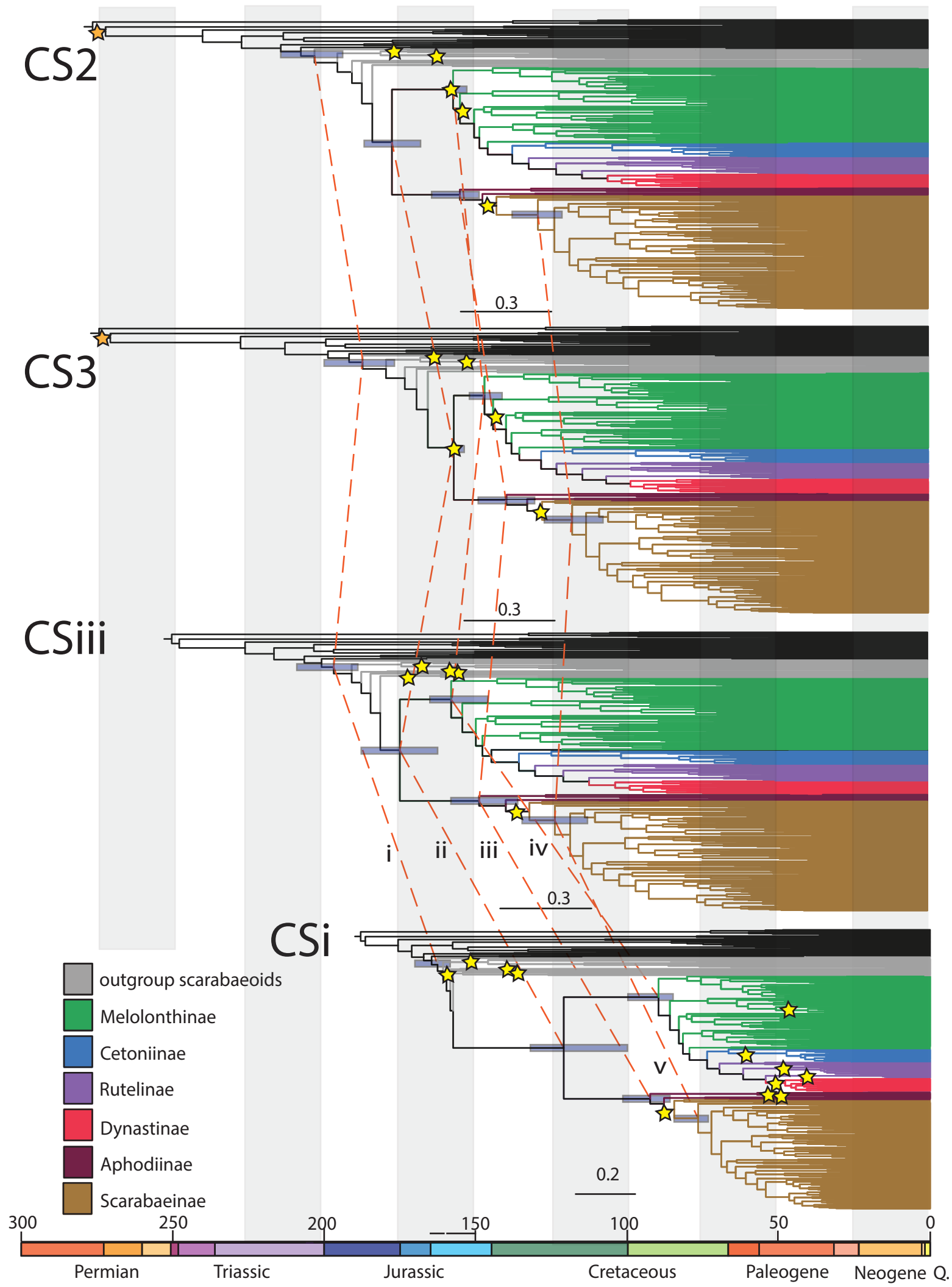

Supplement: S3 Fig — Comparison of dated chronograms for four calibration schemes analyzed in MCMCTree. (PDF) [file pone.0153570.s003.pdf]

A

CS2

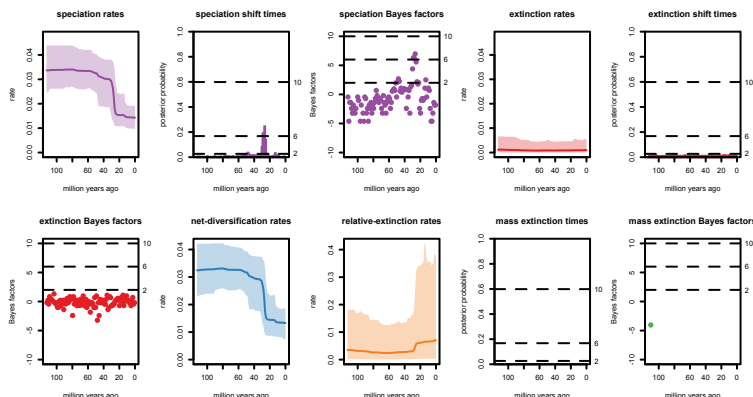

CS3

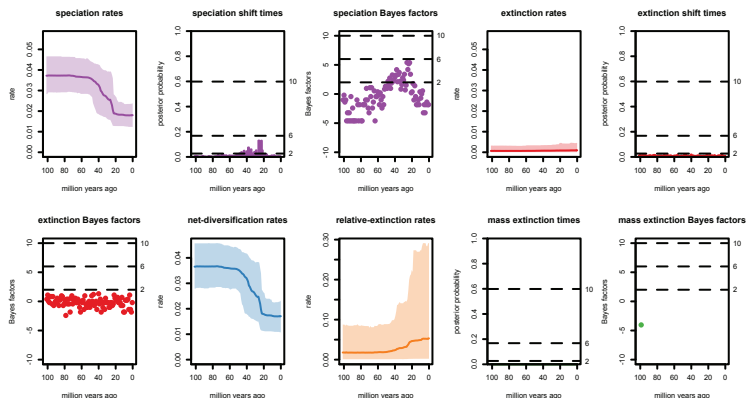

CSiii

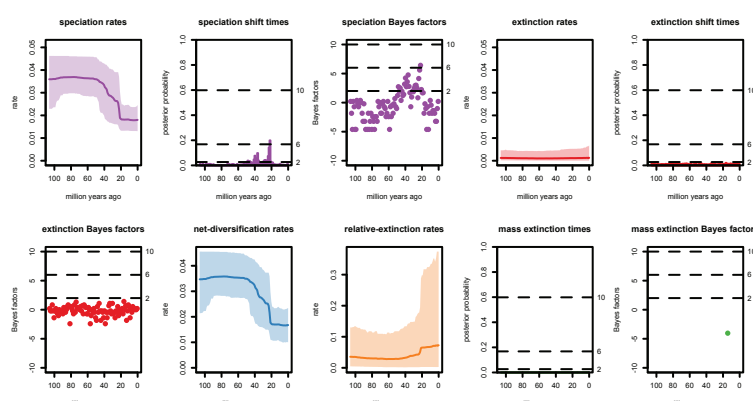

MCMCTree

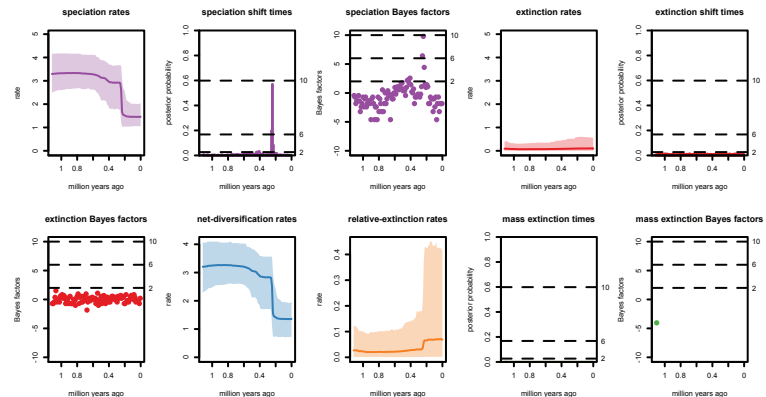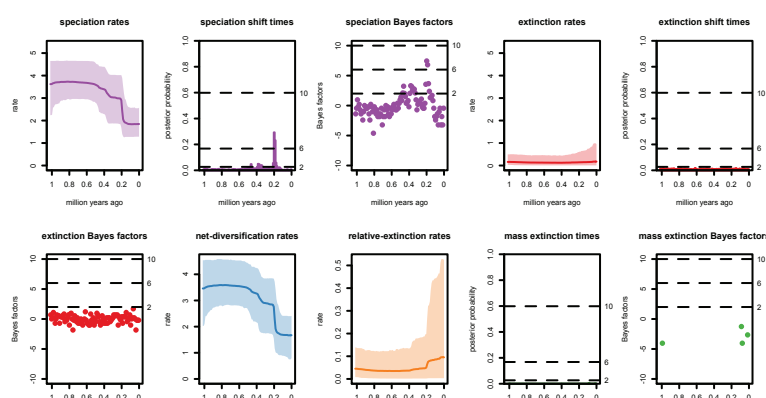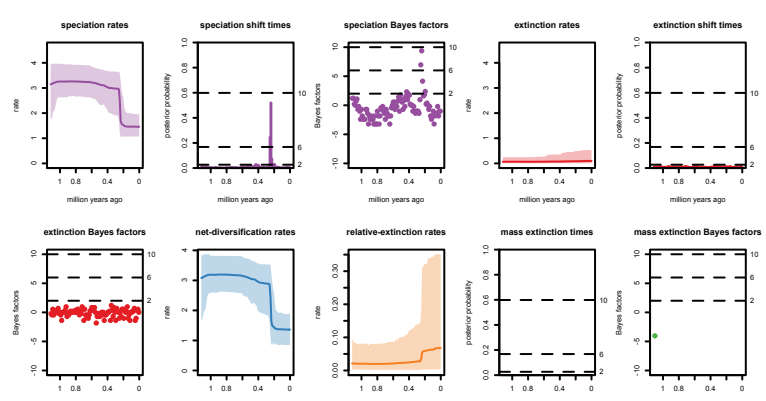

B

CS2

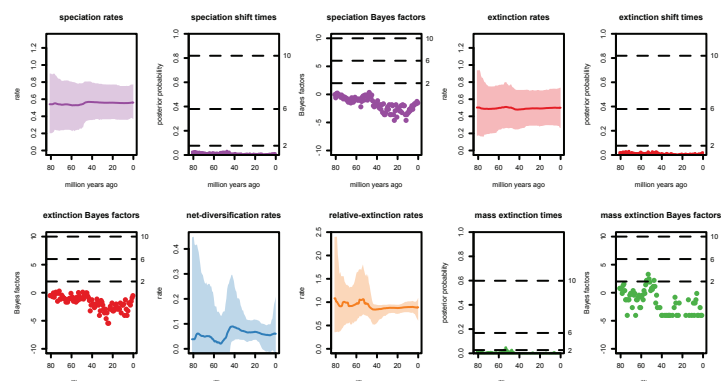

CS3

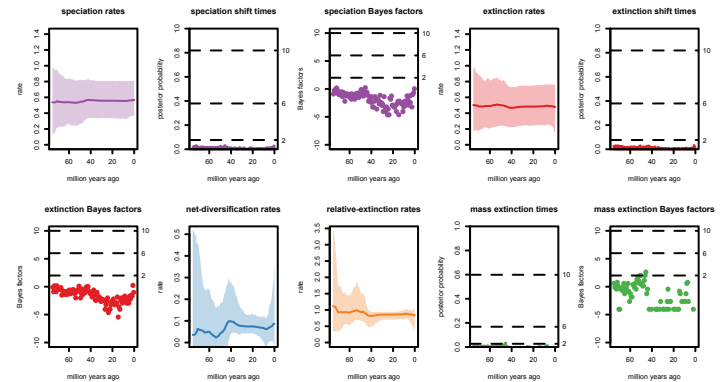

CSiii

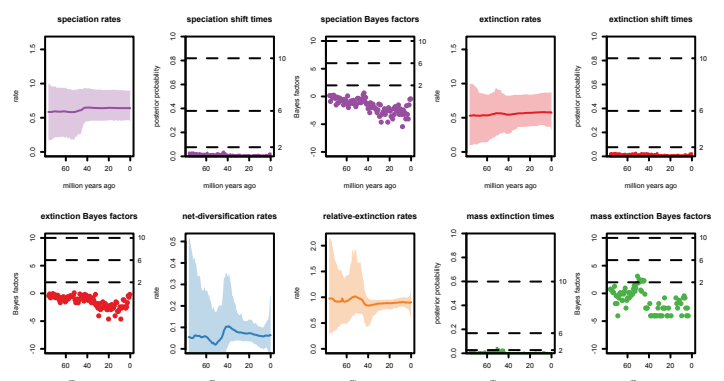

MCMCTree

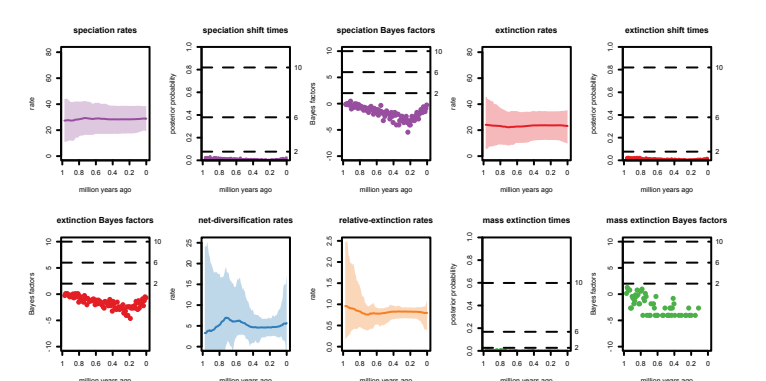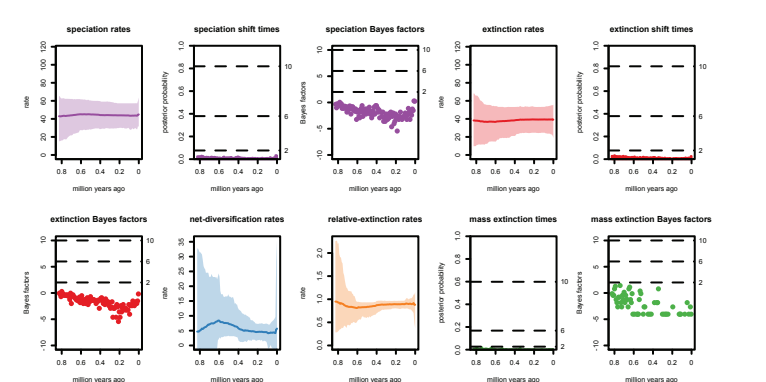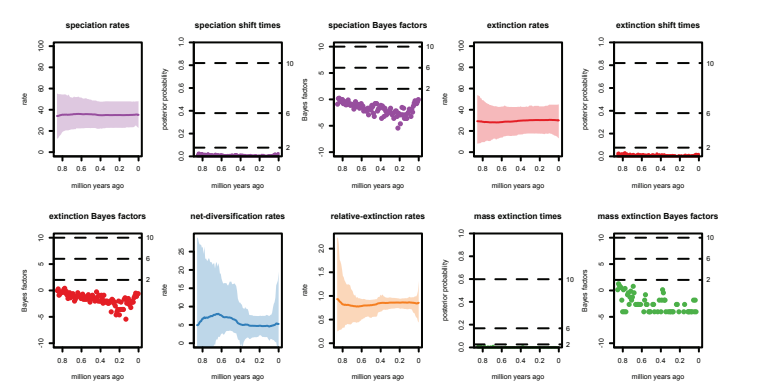

Supplement: S4 Fig — TESS outputs identifying rates and shifts in speciation or extinction, and significant mass-extinction events through time for (A) Pleurosticti (B) Scarabaeinae. Analyses CS2, CS3 and CSiii were examined using output trees from penalized likelihood and Bayesian divergence dating analyses. Posterior mean is represented by a solid bold line and 95% credible interval shadows. Solid vertical bars indicate posterior probability of a rate shift within the interval, with dashed line representing significance thresholds of 2lnBF = 2, 6, or 10 (BF = Bayes Factor). Time scale excludes most recent 45 or 50 Ma depending on analysis that corresponds to species level sampling (see S1 Table for parameters). (PDF) [file pone.0153570.s004.pdf]
