# Supplementary material for: If Dung Beetles (Scarabaeidae: Scarabaeinae) Arose in Association with Dinosaurs, Did They Also Suffer a Mass Co-Extinction at the K-Pg Boundary?
Source: PLoS One. 2016 May 4;11(5):e0153570. doi: 10.1371/journal.pone.0153570 (PMC4856399; doi:10.1371/journal.pone.0153570)
Supplement: S1 Table — Penalized likelihood (PL) output from r8s, Bayesian output from MCMCTree. Sampling fraction (rho) is calculated from the number tips at the corresponding cut time and compared to the predicted diversity calculated using Method of Moments [48]. The most recent 45 or 50 Ma were excluded to minimize effect of limited species level sampling. (DOCX) [file pone.0153570.s005.docx]

Table S1: **Tess analysis settings.** Penalized likelihood (PL) output from r8s, Bayesian output from MCMCTree. Sampling fraction (rho) is calculated from the number tips at the corresponding cut time and compared to the predicted diversity calculated using Method of Moments [48]. The most recent 45 or 50 Ma were excluded to minimize effect of limited species level sampling.

| Clade | Partition | Analysis | Cut time | Tips/nodes | Rho | Diversification (variance/acc. Prob) | Turnover  (variance/acc. Prob) |
| --- | --- | --- | --- | --- | --- | --- | --- |
| Scarabaeinae | CS2 | PL | 50Ma | 125/92 | 0.0638 | 0.208/0.437 | 0.067 /0.458 |
|  | CS2 | Bayesian | 45Ma | 129/128 | 0.0803 | 0.171/ 0.438 | 0.077 /0.492 |
|  | CS3 | PL | 45Ma | 131/98 | 0.0612 | 0.212/ 0.416 | 0.058 /0.501 |
|  | CS3 | Bayesian | 45Ma | 120/191 | 0.0747 | 0.163 / 0.477 | 0.077 /0.423 |
|  | CSiii | PL | 45Ma | 129/96 | 0.0611 | 0.222/0.411 | 0.071 /0.420 |
|  | CSiii | Bayesian | 45Ma | 125/124 | 0.0778 | 0.173/ 0.439 | 0.088 /0.421 |
| Pleurosticti | CS2 | PL | 45Ma | 173/150 | 0.0204 | 0.206/ 0.409 | 1.833/ 0.447 |
|  | CS2 | Bayesian | 45Ma | 171/170 | 0.0231 | 0.192/ 0.435 | 2.043/ 0.437 |
|  | CS3 | PL | 45Ma | 167/144 | 0.0196 | 0.222/ 0.422 | 1.725/ 0.465 |
|  | CS3 | Bayesian | 45Ma | 167/166 | 0.0225 | 0.190/ 0.444 | 1.779/ 0.446 |
|  | CSiii | PL | 45Ma | 173/150 | 0.0204 | 0.205/ 0.437 | 2.102/ 0.402 |
|  | CSiii | Bayesian | 45Ma | 171/170 | 0.0231 | 0.211/ 0.404 | 1.776/ 0.458 |
